# One-pot multistep mechanochemical synthesis of fluorinated pyrazolones

**DOI:** 10.3762/bjoc.13.189

**Published:** 2017-09-14

**Authors:** Joseph L Howard, William Nicholson, Yerbol Sagatov, Duncan L Browne

**Affiliations:** 1 School of Chemistry, Cardiff University, Main Building, Park Place, Cardiff, CF10 3AT, UK

**Keywords:** fluorination, heterocycles, mechanochemistry, multistep, solid-state synthesis

## Abstract

Solventless mechanochemical synthesis represents a technique with improved sustainability metrics compared to solvent-based processes. Herein, we describe a methodical process to run one solventless reaction directly into another through multistep mechanochemistry, effectively amplifying the solvent savings. The approach has to consider the solid form of the materials and compatibility of any auxiliary used. This has culminated in the development of a two-step, one-jar protocol for heterocycle formation and subsequent fluorination that has been successfully applied across a range of substrates, resulting in 12 difluorinated pyrazolones in moderate to excellent yields.

## Introduction

Mechanochemical methods are emerging as an alternative approach to traditional solvent-based reactions for chemical synthesis. Under mechanochemical conditions reactions are performed between neat reagents and do not require a solvent. Processing chemical reactions in such a manner is desirable as reactions are consequently less wasteful and more environmentally benign than the analogous solution-based approaches, especially if the work-up and purification processes can also be made solventless or solvent minimised [1–2]. As such, there is now a significant number of mechanochemical synthetic transformations reported [3–6]. However, for the synthetic community, perhaps the most interesting examples of mechanochemical reactions are not those that are merely solventless but those in which different reactivity or selectivity arises, as well as those that are significantly shorter in reaction time than those conducted in solution. Indeed, there are several examples where reactions are clearly significantly faster under mechanochemical conditions [7–8].

One of several challenges to be overcome for the further development of mechanochemistry as an up to date tool for synthesis is to gain a better insight into the ability to run multistep procedures. One-pot multistep procedures are particularly efficient, in that the same reaction vessel is used for each step, additional reagents are simply added to the reaction mixture at each stage with no isolation of intermediates or removal of side products [9]. One-pot procedures require the conditions for each step to be compatible with succeeding steps. Typical problems encountered when attempting to multistep reactions include solvent compatibility, or, issues with side products that can inhibit future steps, e.g., by providing access to alternative reaction pathways, poisoning catalysts or altering the pH unfavourably [9]. With regards to mechanochemistry such processing serves to amplify the sustainability metrics by running back-to-back solventless reactions. Multistep mechanochemical procedures have been successfully applied to the synthesis of *O*-glycosides [10], bioactive hydantoins [11], extended iptycenes [12] and organometallics [13] where problems can occur using solution-based synthesis due to limited solubility. Whilst mechanochemical one-pot procedures offer the inherent ability to overcome the issue of identifying a solvent compatible with several consecutive steps, we envisaged alternative hurdles not previously described with regard to compatibility of chemical form. The state of reagents or chemical form is significant to reactions conducted under mechanochemical conditions, where liquids and solids behave differently. For instance, when liquid components are used it may be critical to add a solid auxiliary that helps the transfer of energy and mass (adequate mixing) throughout the mixture. In many cases, leaving out such an auxiliary material can result in a gum or a paste that does not mix well and results in low reaction conversions. Clearly the presence of such a material may have a knock-on effect on any multistep process. Liquid-assisted grinding (LAG) is another phenomenon that can provide enhancement to the reaction outcome and again should be considered for use in a multistep format [14–16].

**Scheme 1:**
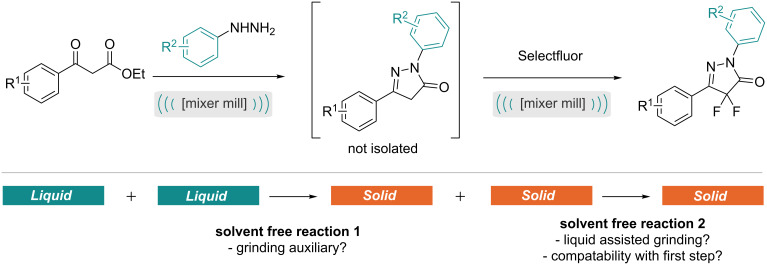
Factors to be considered regarding the physical form in the one-pot two-step mechanochemical procedure.

Having recently begun our research programme in the area of mechanochemistry, we were particularly intrigued by the compatibility of differing chemical forms and additives across a two-step, one-grinding jar solventless process. To investigate this we designed a 2-step reaction related to our recent work on liquid assisted grinding effects of the fluorination of 1,3-dicarbonyl compounds, in which the dicarbonyl will initially form a pyrazolone in the first reaction prior to undergoing difluorination in the second step (Scheme 1) [17].

Notably this approach will likely require a grinding auxiliary in the first step where two liquid phases react and will be catalysed by an acid to afford a solid pyrazolone material. This will then be followed by a difluorination reaction between solid–solid reactants, this reaction may perform better in the presence of base in the second step. In this report, we present a systematic approach to finding the optimal conditions, which are most compatible with both steps. Notably, fluorinated pyrazolones have the potential to be useful pharmaceutical or agrochemical products, given the desirable properties that can be obtained on introduction of fluorine to a molecule [18–25]. However, there have been limited reports on the synthesis of fluorinated pyrazoles, but fluorinated pyrazolones remain poorly studied [26–30].

## Results and Discussion

Initially the mechanochemical pyrazolone formation was investigated as the first step of the two step process, we opted to keep the ball size, ball number, jar size and jar and ball material as in our previous studies to reduce the number of variables for this analysis [17]. In the first instance, simply milling the two liquids in the absence of an auxiliary material resulted in a poor yield (Table 1, entry 1). Pleasingly, treatment of ethyl benzoylacetate with one equivalent of phenylhydrazine in the presence of sodium chloride afforded the desired pyrazolone product in 66% yield after milling for 10 minutes (Table 1, entry 2). The addition of a grinding auxiliary could play several roles. We propose that the key benefits are related to improved mixing, and aiding in energy transfer, specifically in mechanochemical reactions where the reaction mixture could be described as a gum, paste or liquid. Notably, the comparable reaction under solvent-based conditions (in toluene, under reflux) required 24 hours to achieve a similar yield (Table 1, entry 3).

**Table 1:** Optimisation of pyrazolone formation.

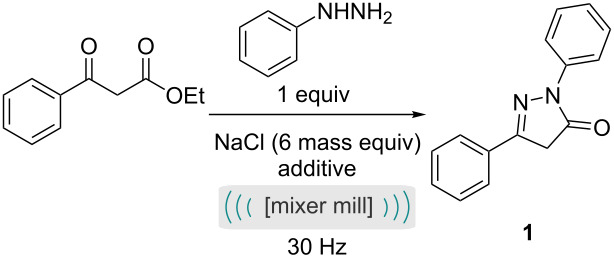			

Entry	Additive (equiv)	Time [min]	Yield^a^

1^b^	–	10	20%
2	–	10	66%
3^c^	–	1440	58%
4	HCl (0.5)	10	43%
5	tosic acid (0.5)	10	37%
6	oxalic acid (0.5)	10	22%
7	citric acid (0.5)	10	38%
8	benzoic acid (0.5)	10	88%
9	acetic acid (0.5, 30 μL)	10	88%
10	acetic acid (0.08, 5 μL)	10	75%
11	acetic acid (1.7, 100 μL)	10	97%
12	acetic acid (4.2, 250 μL)	10	73%
13	acetic acid (0.5)	20	86%
**14**	**acetic acid (0.5)**	**40**	**97% (92%^d^)**
15	acetic acid (0.5)	60	97%
16	acetic acid (0.5)	120	97%
17^b^	acetic acid (0.5)	1440	80%

^a^Determined by ^1^H NMR using mesitylene as an internal standard. ^b^Mechanochemical reaction with no NaCl. ^c^Solvent based reaction: heating under reflux in toluene, no NaCl. ^d^Isolated yield.

As pyrazolone formation can be catalysed by acid, a screen of both solid and liquid acids was next performed (Table 1, entries 4–9). In general, the weaker carboxylate acids performed better than mineral acids, with the highest yield obtained using acetic acid (Table 1, entry 9). The quantity of acid used was then varied. In general, the yield increased with an increase in the amount of acid used (Table 1, entries 9–12), this was with the exception of 250 μL or 4.2 equivalents (Table 1, entry 12), where the yield dropped. The latter observation may be due to the larger amount of liquid altering the texture of the reaction mixture and thus reducing effective mixing. An alternative justification is that at higher acid equivalents in the solid state the ‘on–off’ protonation of the hydrazine is slow, meaning that the nucleophilicity is greatly retarded compared to lower acid loadings. Nonetheless, considering that the subsequent fluorination step should proceed optimally under basic conditions [17], the lowest amount of acid which also provided a good yield was thus chosen; 30 μL (Table 1, entry 9). Finally, the reaction time with this quantity of acid was then optimised, whereupon the reaction was found to be complete after 40 minutes producing 92% isolated yield of pyrazolone **1** (Table 1, entry 14). For comparison, these optimal conditions have been applied to a solution-based reaction, resulting in a poorer yield after 24 hours at reflux in toluene (Table 1, entry 17). Having achieved optimal conditions for the first step of the reaction, our attention turned to the second step.

Initial investigation of the fluorination of the pyrazolone focused on finding the optimum reaction time for the isolated step rather than two-step, i.e., the pyrazolone material was isolated from step one and purified before subjecting to this second reaction optimisation. With no additives, the fluorination was complete after 2 hours (Table 2, entry 4), notably an extra hour returned no further improvement (Table 2, entry 5). The fluorination reaction studied here proceeds via an enolate which is aromatic and therefore is relatively facile (compared to the fluorination of other heterocyclic systems). Introduction of a mild base, such as sodium carbonate to the reaction vessel served to enhance the rate of reaction, providing complete conversion after 1 hour (Table 2, entry 6).

**Table 2:** Optimisation of pyrazolone fluorination.

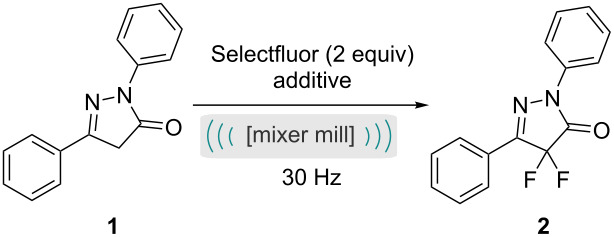			

Entry	Additive (equiv)	Time [min]	Yield^a^

**1**	–	10	11%
**2**	–	30	41%
**3**	–	60	83%
**4**	–	120	95%
**5**	–	180	94%
**6**	Na_2_CO_3_ (1.0)	60	100%
**7**	NaCl (6.0)^b^	120	68%
**8**	acetic acid (0.5)	120	75%
**9**	NaCl (6.0)^b^, Na_2_CO_3_ (1.0)	60	100%

^a^Determined by ^19^F NMR. ^b^Mass equivalents NaCl.

With an understanding of the second step we then assessed the reaction whilst mimicking aspects of the first reaction in order to look for compatibility of a two-step one-jar process. The most important difference between the two steps is the physical state of the reactants. For the first step (Table 1), both reagents are liquids, and a grinding auxiliary was required to aid mixing and energy transfer. However, for the second step (Table 2), the reagents are solids, and the presence of a grinding agent could have a diluting effect. Indeed, addition of sodium chloride does slow down the fluorination, giving a poorer yield (Table 2, entry 7). Another factor to be explored was the effect of acetic acid on the second step. Again, this resulted in a decrease in yield of the fluorination reaction achievable within a two hour reaction time (Table 2, entry 8).

**Scheme 2:**
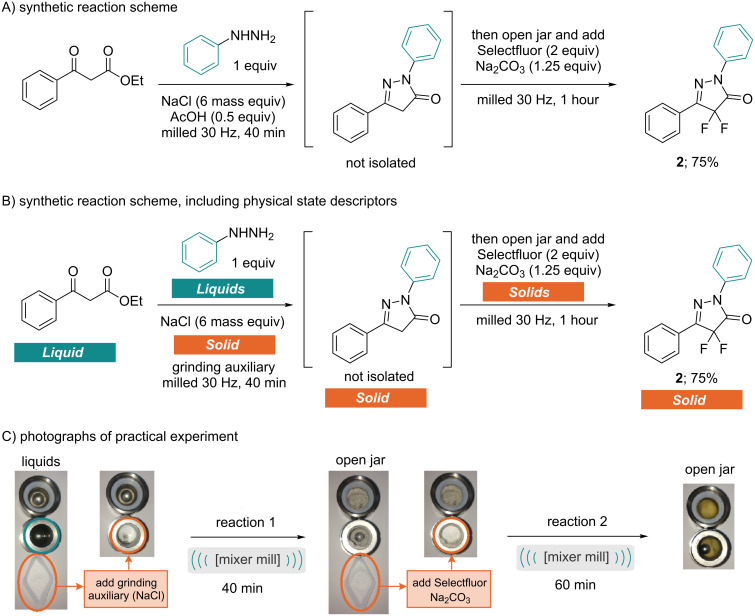
Optimised conditions for the one-pot synthesis.

Pleasingly a combination of sodium carbonate with the sodium chloride grinding auxiliary resulted in complete reaction after one hour (Table 2, entry 9). The only compatibility issue remaining was the acid present from the first step. However, as a base improved the reactivity of the fluorination, the final conditions make use of enough sodium carbonate both to neutralise the remaining acid and accelerate the second step. By applying these compatible conditions to the one-pot procedure, the desired fluorinated pyrazolone was isolated in 75% yield (Scheme 2). Scheme 2 also shows the physical state descriptors and photographs of the practical experiment.

**Scheme 3:**
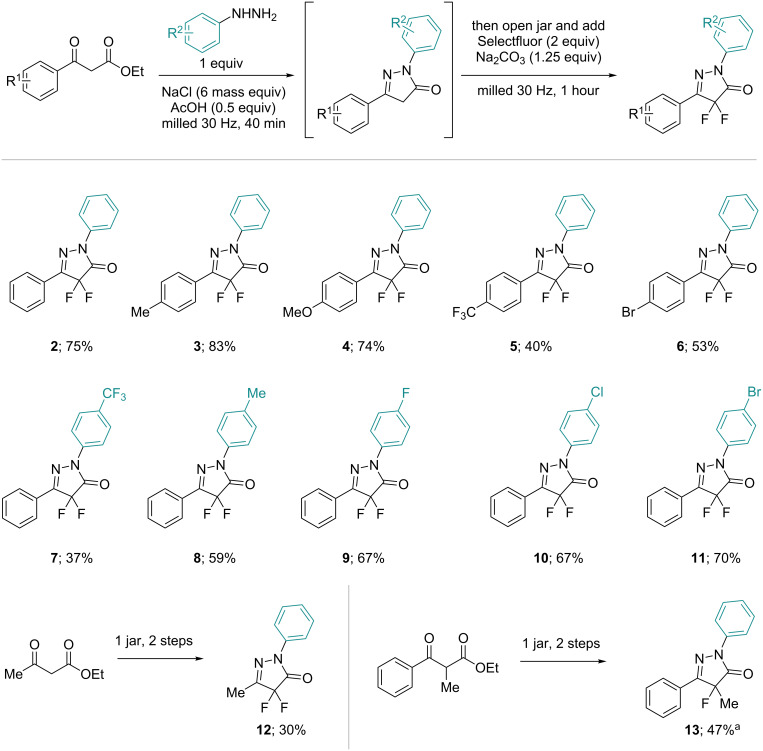
Substrate scope of the one-pot, 2 step mechanochemical synthesis (isolated yields). ^a^1 equiv Selectfluor used.

With suitable conditions in hand, the scope of this one-pot mechanochemical process was explored (Scheme 3). Initially, the scope of β-ketoesters was assessed and the procedure was found to be compatible with both the electron-withdrawing and electron-donating groups. However, a poorer yield was obtained for the electron-withdrawing trifluoromethyl substituent (**5**). The scope of phenylhydrazines was also briefly investigated, with several examples demonstrating good isolated yields, again an electron-withdrawing trifluoromethyl substituent was an exception to this (**7**) [31]. For this case, crude ^19^F NMR after the first step shows a 41% conversion, suggesting that the pyrazolone formation is the limiting factor in this example. An alkyl β-ketoester (ethyl acetoacetate) was also used, affording methyl substituted difluoropyrazolone **12** in modest yield. Finally, an α-substituted β-ketoester was successfully converted to the pyrazolone before monofluorination using one equivalent of Selectfluor to prepare pyrazolone **13**, also in moderate yield. In general the optimised approach seems to apply to a small range of compounds.

## Conclusion

In summary, we have developed a one-pot, two-step mechanochemical synthesis of fluorinated pyrazolones. The experiments provide a logical approach to multistep solventless synthesis under milling conditions and more broadly will assist in the conversion of other processes to such a system. After careful consideration of physical form and additive compatibility the final protocol has been successfully applied to the preparation of a small library of 12 difluorinated pyrazolones, several of which are hitherto unreported.

## Supporting Information

Information about the data that underpins the results presented in this article, including how to access them, can be found in the Cardiff University data catalogue at doi.org/10.17035/d.2017.0038572887.

File 1 Experimental part.
