# Supplementary material for: One-pot multistep mechanochemical synthesis of fluorinated pyrazolones
Source: Beilstein J Org Chem. 2017 Sep 14;13:1950–6. doi: 10.3762/bjoc.13.189 (PMC5629391; doi:10.3762/bjoc.13.189)
Supplement: File 1 — Experimental part. [file Beilstein_J_Org_Chem-13-1950-s001.pdf]

Supporting Information

for

One-pot multistep mechanochemical synthesis of fluorinated pyrazolones

Joseph L. Howard[1], William Nicholson[1], Yerbol Sagatov[1] and Duncan L. Browne*[1]

Address: [1]School of Chemistry, Cardiff University, Main Building, Park Place, Cardiff, CF10 3AT, UK

Email: d Duncan L. Browne - lbrowne@cardiff.ac.uk

* Corresponding author

Experimental part

General methods **S2**

Synthesis of β-ketoesters **S3**

Multistep one pot mechanochemical synthesis of fluorinated pyrazolones **S6**

Spectroscopic data **S13**

- **β-ketoesters** **S13**
- **Fluorinated pyrazolones** **S19**

References **S43**

General methods

^{1}H, ^{19}F and ^{13}C NMR spectra were obtained on Bruker 400 Ultrashield™ and Bruker Ascend™ 500 MHz spectrometers with chloroform-*d* as deuterated solvent. The obtained chemical shifts; δ, are reported in ppm and are referenced to the residual solvent signal. Spin-spin coupling constants; *J,* are given in Hz.

High resolution mass spectral (HRMS) data were obtained on a Thermo Scientific LTQ Orbitrap XL by the EPSRC UK National Mass Spectrometry Facility at Swansea University or on a Waters MALDI-TOF mx in Cardiff University.

Infrared spectra were recorded on a Shimadzu IR-Affinity-1S FTIR spectrometer.

Melting points were measured using a Gallenkamp apparatus and are reported uncorrected.

The ball mill used was a Retsch MM 400 mixer mill. Unless otherwise stated, mechanochemical reactions were performed in 10 mL stainless steel jars with one stainless steel ball of mass 4 g.

All chemicals were obtained from commercial sources and used without further purification unless stated otherwise. Dry THF was obtained from a solvent purification system. Petroleum ether referes to the 40–60 °C fraction.

Synthesis of β-ketoesters

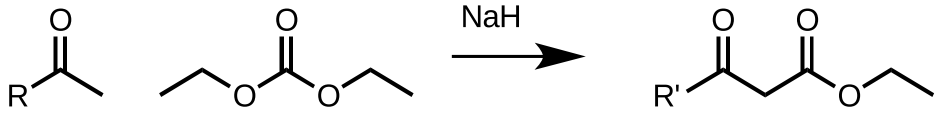

General procedure (GP1)

Following a literature procedure[1]: to a suspension of NaH (1.2 g, 30 mmol, 60% in mineral oil) in dry THF (10 mL) was added diethyl carbonate (4.85 mL, 40 mmol) in oven-dried glassware under N_2. A solution of the corresponding ketone (10 mmol) in dry THF (5 mL) was added slowly and the reaction mixture heated under reflux for 6 hours. The reaction mixture was quenched with glacial acetic acid (1 mL) and HCl (10%, 20 mL). The aqueous phase was extracted with ethyl acetate (3 × 10 mL) and the combined organic phase washed with saturated sodium hydrogen carbonate (10 mL), water (10 mL) and brine (10 mL). The combined organic phase was dried ($MgSO_4$), filtered and the solvent removed under reduced pressure to yield the crude product. This was purified by flash column chromatography on silica gel (gradient elution EtOAc in petroleum ether (0–25%)).

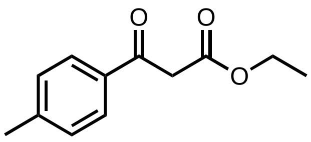

Ethyl 3-oxo-3-(*p*-tolyl)propanoate[2]

Prepared according to **GP1** with further purification by short-path distillation (190 °C, 7 mbar); 0.583 g, 2.8 mmol, 28%, yellow oil. 1:3.7 enol:keto.

^{1}H NMR (400 MHz; $CDCl_3$): δ 12.60 (s, enol 1H), 7.86 (d, *J* = 8.2 Hz, 2H), 7.69 (d, *J* = 8.1, enol 2H), 7.29 (d, *J* = 8.1 Hz, 2H), 7.24 (d, *J* = 7.9 Hz, enol 2H), 5.65 (s, enol 1H), 4.27 (q, *J* = 7.1 Hz, enol 2H), 4.23 (q, *J* = 7.1 Hz, 2H), 3.98 (s, 2H), 2.44 (s, 3H), 2.41 (s, enol 3H), 1.35 (t, *J* = 7.1 Hz, enol 3H), 1.28 (t, *J* = 7.1 Hz, 3H).

^{13}C NMR (101 MHz; $CDCl_3$): δ 192.2, 173.4, 171.7, 167.8, 144.8, 141.8, 133.7, 130.8, 129.6, 129.4, 128.8, 126.1, 86.8, 61.6, 60.4, 46.1, 21.8, 21.6, 14.5, 14.2.

IR: 2980, 1736, 1682, 1265, 1182, 1144, 808 cm^{-1}.

HRMS (AP+): $[C_{12}H_{14}O_3 + H]^+$ calc. 207.1021, found 207.1024.

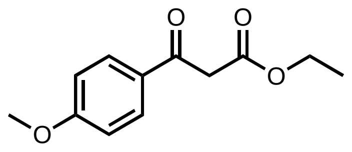

Ethyl 3-(4-methoxyphenyl)-3-oxopropanoate[3]

Prepared according to **GP1** with further purification by short-path distillation (200 °C, 8 mbar); 0.434 g, 2.0 mmol, 20%, colourless oil. 1:18 enol:keto.

^{1}H NMR (400 MHz; $CDCl_3$): δ 12.63 (s, enol 1H), 7.93 (d, *J* = 9.1 Hz, 2H), 7.74 (d, *J* = 9.1 Hz, enol 2H), 6.95 (m, 2H), 5.58 (s, enol 1H), 4.25 (q, *J* = 7.1 Hz, enol 2H), 4.22 (q, *J* = 7.1 Hz, 2H) 3.94 (s, 2H), 3.88 (s, 3H), 3.85 (s, enol 3H), 1.33 (t, *J* = 7.1 Hz, enol 3H), 1.26 (t, *J* = 7.1 Hz, 3H).

^{13}C NMR (101 MHz; $CDCl_3$): δ 191.1, 167.9, 164.1, 131.0, 129.3, 114.1, 61.6, 55.7, 46.0, 14.2.

IR: 2980, 1734, 1674, 1597, 1256, 1024, 843 cm^{-1}.

HRMS (pNSI+): $[C_{12}H_{14}O_4 + H]^+$ calc. 223.0965, found 223.0964.

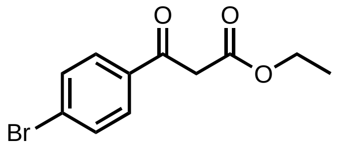

Ethyl 3-(4-bromophenyl)-3-oxopropanoate[3]

Prepared according to **GP1**; 2.395 g, 8.8 mmol, 88%, yellow oil. 1:2.2 enol:keto.

^{1}H NMR (400 MHz; $CDCl_3$): δ 12.56 (s, enol 1H), 7.81 (d, *J* = 8.5 Hz, 2H), 7.63 (m, 2H), 7.55 (d, *J* = 8.6 Hz, enol 2H), 5.64 (s, enol 1H), 4.30 (q, *J* = 7.3 Hz, enol 2H), 4.18 (q, *J* = 7.3 Hz, 2H), 3.95 (s, 2H), 1.33 (t, *J* = 7.1 Hz, enol 3H), 1.25 (t, *J* = 7.1 Hz, 3H).

^{13}C NMR (101 MHz; $CDCl_3$): δ 191.6, 173.2, 170.3, 167.3, 134.9, 132.5, 132.3, 131.9, 130.1, 129.2, 127.7, 125.9, 87.9, 61.8, 60.6, 46.1, 14.4, 14.2.

IR: 2980, 1734, 1686, 1585, 1260, 1194, 995, 800 cm^{-1}.

HRMS (pNSI+): $[C_{11}H_{11}O_3Br + H]^+$ calc. 270.9964, found 270.9957.

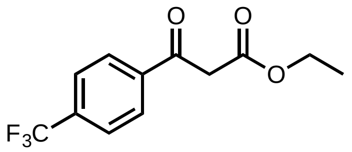

Ethyl 3-oxo-3-(4-(trifluoromethyl)phenyl)propanoate[4]

Prepared according to **GP1**; 1.701 g, 6.5 mmol, 65%, yellow oil. 3:2 enol:keto.

^{1}H NMR (400 MHz; $CDCl_3$): δ 12.57 (s, enol 1H), 8.06 (m, 2H), 7.88 (m, enol 2H), 7.76 (m, 2H), 7.68 (m, enol 2H), 5.72 (s, enol 1H), 4.31 (q, *J* = 7.1 Hz, enol 2H), 4.19 (q, *J* = 7.1 Hz, 2H) 4.01 (s, 2H), 1.39 (t, *J* = 7.1 Hz, enol 3H), 1.21 (t, *J* = 7.1 Hz, 3H).

^{19}F NMR (376 MHz; $CDCl_3$): δ -62.9 (enol 3F), -63.2 (3F).

^{13}C NMR (101 MHz; $CDCl_3$): δ 191.7, 173.0, 169.5, 167.1, 138.7, 136.9, 135.0 (q, *J* = 32.7 Hz), 132.8 (q, *J* = 32.7 Hz), 129.0, 126.5, 125.9 (q, *J* = 3.7 Hz), 125.6 (q, *J* = 3.7 Hz), 123.9 (q, *J* = 272.7 Hz), 123.6 (q, *J* = 272.7 Hz), 89.1, 61.8, 60.7, 46.2, 14.30, 14.10.
IR: 2980, 1744, 1695, 1616, 1319, 1260, 1111, 1065, 853, 802 cm^{-1}.
HRMS (pNSI+): $[C_{12}H_{11}F_3O_3 + H]^+$ calc. 261.0733, found 261.0736.

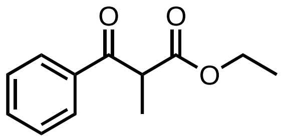

Ethyl 2-methyl-3-oxo-3-phenylpropanoate[2]

Following a literature procedure[5]: To a suspension of sodium hydride (0.788 g, 19.5 mmol, 60% in mineral oil) in dry THF (20 mL) was added dropwise ethyl benzoylacetate (3.75 g, 19.5 mmol). When the gas evolution stopped, methyl iodide (1.2 mL, 19.5 mmol) was added slowly. The reaction mixture was stirred for a further 20 hours at room temperature then quenched with a saturated aqueous solution of ammonium chloride (30 mL). The phases were separated and the aqueous phase further extracted with ethyl acetate (3 × 20 mL). The combined organic layers were dried ($MgSO_4$), filtered and the solvent removed to yield the product as a yellow oil (4.01 g, 99%).
^{1}H NMR (400 MHz; $CDCl_3$): δ 7.98 (d, *J* = 7.1 Hz, 2H), 7.58 (t, *J* = 7.4 Hz, 1H), 7.49-7.45 (m, 2H), 4.37 (q, *J* = 7.1 Hz, 1H), 4.14 (q, *J* = 7.1 Hz, 2H), 1.49 (d, *J* = 7.1 Hz, 3H), 1.16 (t, *J* = 7.1 Hz, 3H).
^{13}C NMR (101 MHz; $CDCl_3$): δ 196.0, 171.0, 136.0, 133.6, 128.84, 128.70, 61.5, 48.5, 14.1, 13.9.
IR: 2980, 1734, 1684, 1375, 957 cm^{-1}.
HRMS (pNSI+): $[C_{12}H_{14}O_3 + H]^+$ calc. 207.1016, found 207.1014.

Multistep one pot mechanochemical synthesis of fluorinated pyrazolones

General procedure (GP2)

To a 10 mL stainless steel milling jar was added the β-ketoester (1 mmol), the hydrazine (1 mmol), sodium chloride (six times the total mass of reagents) and glacial acetic acid (30 μL, 0.5 mmol). The ball was added and the mixture milled at 30 Hz for 40 minutes. Following this initial grinding period, Selectfluor (0.708 g, 2 mmol) and sodium carbonate (0.133 g, 1.25 mmol) were added to the reaction mixture. The jar was hand sealed and milled for a further 60 minutes at 30 Hz (Scheme S1). The resulting powder was transferred into a flask, washing the residue with dichloromethane (approximately 40 mL). The insoluble material was removed by filtration. The solvent was removed under reduced pressure to yield the crude product. This was purified by flash column chromatography on silica gel (gradient elution EtOAc (0–5%) in petroleum ether).

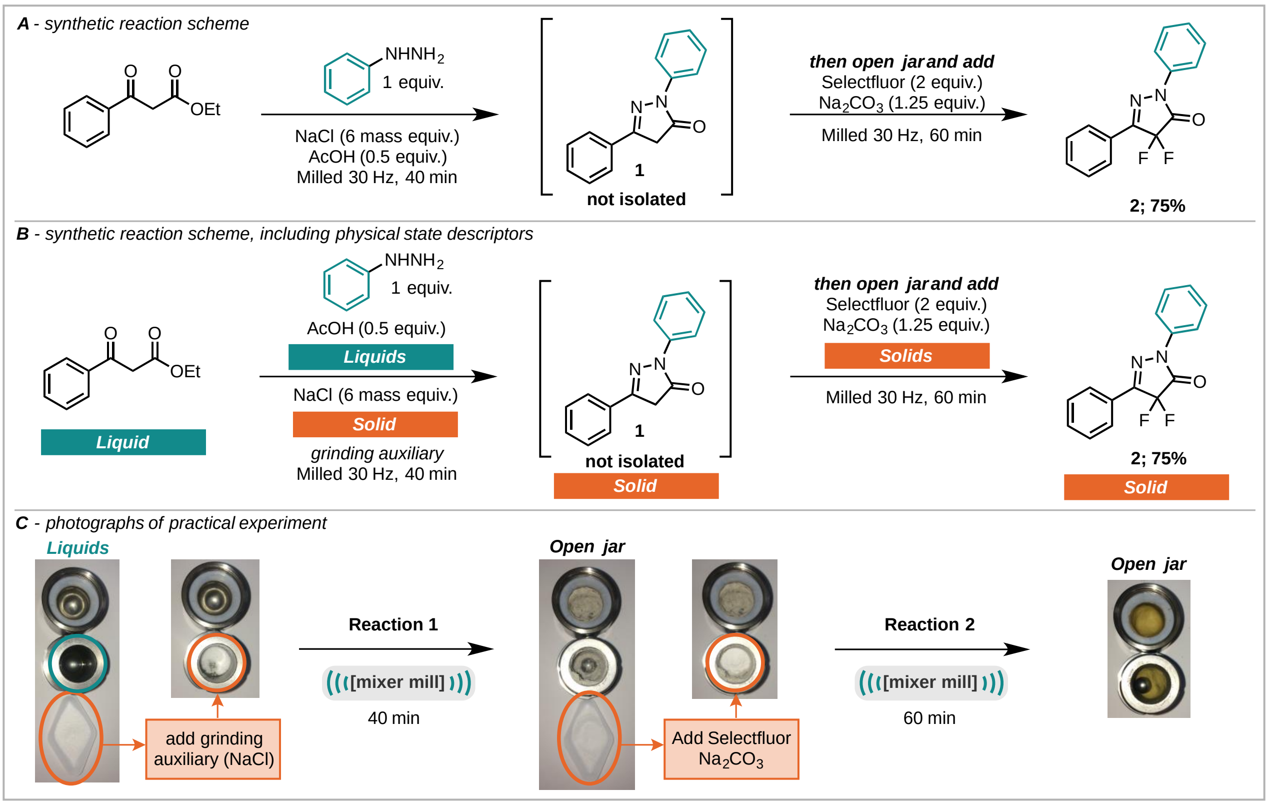

Scheme S1: One-pot procedure for mechanochemical synthesis of fluorinated pyrazolones.

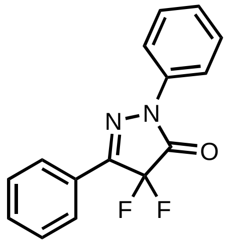

4,4-Difluoro-2,5-diphenyl-2,4-dihydro-3*H*-pyrazol-3-one, 2[6]

Prepared according to **GP2**; 0.205 g, 0.75 mmol, 75%, orange powder.

^{1}H NMR (400 MHz; $CDCl_3$): δ 7.98 (m, 4H), 7.52 (m, 5H), 7.30 (t, *J* = 7.4 Hz, 1H).

^{19}F NMR (376 MHz; $CDCl_3$): δ -115.7.

^{13}C NMR (126 MHz; $CDCl_3$): δ 159.3 (t, *J* = 29 Hz), 149.8 (t, *J* = 25 Hz), 136.8, 132.3, 129.33, 129.25, 126.8, 126.52, 126.37, 118.8, 109.2 (t, *J* = 265 Hz).

IR: 1736, 1491, 1410, 1179, 1101, 756, 737, 664, 631 cm^{-1}.

HRMS (AP+): $[C_{15}H_{10}N_2F_2O + H]^+$ calc. 273.0839, found 273.0842.

mp: 77-78 °C (ethyl acetate).

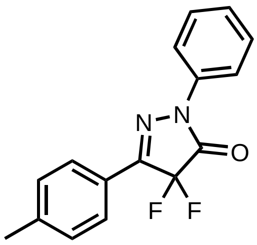

4,4-Difluoro-2-phenyl-5-(*p*-tolyl)-2,4-dihydro-3*H*-pyrazol-3-one, 3

Prepared according to **GP2**; 0.238 g, 0.83 mmol, 83%, Yellow powder.

^{1}H NMR (400 MHz; $CDCl_3$): δ 7.95 (d, *J* = 7.8 Hz, 2H), 7.87 (d, *J* = 7.9 Hz, 2H), 7.48 (t, *J* = 8.0 Hz, 2H), 7.33 (m, 3H), 2.45 (s, 3H).

^{19}F NMR (471 MHz; $CDCl_3$): δ -115.4.

^{13}C NMR (126 MHz; $CDCl_3$): δ 159.3 (t, *J* = 30 Hz), 149.9 (t, *J* = 21 Hz), 143.2, 136.9, 130.1, 129.2, 126.8, 126.4, 123.7, 118.8, 109.3 (t, *J* = 258 Hz), 21.8.

IR: 1738, 1493, 1261, 1103, 743, 689 cm^{-1}.

HRMS (AP+): $[C_{16}H_{12}N_2F_2O + H]^+$ calc. 287.0996, found 287.0999.

mp: 112-113 °C (ethyl acetate).

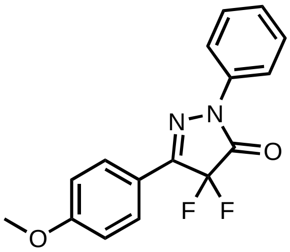

4,4-Difluoro-5-(4-methoxyphenyl)-2-phenyl-2,4-dihydro-3*H*-pyrazol-3-one, 4

Prepared according to **GP2**; 0.224 g, 0.74 mmol, 74%, Yellow powder.

^{1}H NMR (500 MHz; $CDCl_3$): δ 7.94 (m, 4H), 7.47 (t, *J* = 8.0 Hz, 2H), 7.29 (t, *J* = 7.4 Hz, 1H), 7.02 (d, *J* = 8.9 Hz, 2H), 3.89 (s, 3H).

^{19}F NMR (471 MHz; $CDCl_3$): δ -115.1.

^{13}C NMR (126 MHz; $CDCl_3$): δ 162.9, 159.2 (t, *J* = 31 Hz), 149.7 (t, *J* = 21 Hz), 137.0, 129.2, 128.8, 126.4, 119.01, 118.86, 114.9, 109.5 (t, *J* = 258 Hz), 55.7.

IR: 1734, 1520, 1497, 1180, 1099, 833, 687 cm^{-1}.

HRMS (AP+): $[C_{16}H_{12}F_2N_2O_2 + H]^+$ calc. 303.0945, found 303.0941.

mp: 105-106 °C (ethyl acetate).

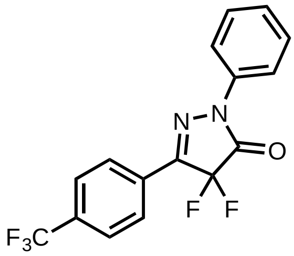

4,4-Difluoro-2-phenyl-5-(4-(trifluoromethyl)phenyl)-2,4-dihydro-3*H*-pyrazol-3-one, 5

Prepared according to **GP2**; 0.137 g, 0.40 mmol, 40%, Orange powder.

^{1}H NMR (400 MHz; $CDCl_3$): δ 8.10 (d, *J* = 8.4 Hz, 2H), 7.93 (d, *J* = 8.0 Hz, 2H), 7.79 (d, *J* = 8.4 Hz, 2H), 7.50 (t, *J* = 8.4 Hz, 2H), 7.32 (m, 1H).

^{19}F NMR (376 MHz; $CDCl_3$): δ -63.2 (3F), -116.2 (2F).

^{13}C NMR (101 MHz; $CDCl_3$): δ 159.1 (t, *J* = 30 Hz), 148.5 (t, *J* = 22 Hz), 136.6, 133.7 (q, *J* = 33 Hz), 129.6, 129.4, 127.1, 126.9, 126.4 (q, *J* = 4 Hz), 123.6 (q, *J* = 274 Hz), 119.0, 108.7 (t, *J* = 259 Hz).

IR: 1742, 1493, 1400, 1321, 1167, 1065, 1015, 934, 825, 733 cm^{-1}.

HRMS (ASAP+): $[C_{16}H_9N_2F_5O + H]^+$ calc.341.0713, found 341.0718.

mp: 112-113 °C (ethyl acetate).

5-(4-Bromophenyl)-4,4-difluoro-2-phenyl-2,4-dihydro-3*H*-pyrazol-3-one, 6

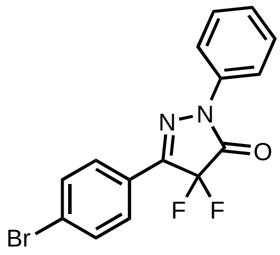

Prepared according to **GP2**; 0.187 g, 0.53 mmol, 53%, Yellow powder.

^{1}H NMR (400 MHz; $CDCl_3$): δ 7.92 (d, *J* = 8.8 Hz, 2H), 7.83 (d, *J* = 8.8 Hz, 2H), 7.67 (d, *J* = 8.8 Hz, 2H), 7.48 (m, 2H), 7.31 (t, *J* = 7.2 Hz, 1H).

^{19}F NMR (471 MHz; $CDCl_3$): δ -115.8.

^{13}C NMR (126 MHz; $CDCl_3$): δ 159.1 (t, *J* = 30 Hz), 149.0 (t, *J* = 21 Hz), 136.6, 132.7, 129.3, 128.1, 127.2, 126.7, 125.2, 118.9, 108.9 (t, *J* = 259 Hz).

IR: 2980, 1740, 1587, 1487, 1256, 1393, 1098, 1069, 934, 745, 685 cm^{-1}.

HRMS (AP+): $[C_{15}H_9N_2F_2OBr + H]^+$ calc. 350.9945, found 350.9945.

mp: 123-124 °C (ethyl acetate).

General Procedure 3 (GP3)

Diethyl ether (10 mL) and sodium hydroxide solution (10 mL, 0.5 M) were added to the hydrazine hydrochloride (2 mmol) and shaken until dissolved. The layers were separated and the aqueous layer further extracted with diethyl ether (2 × 10 mL). The organic phase was dried ($MgSO_4$), filtered and the solvent removed to yield the hydrazine. To a 10 mL stainless steel milling jar was added ethyl benzoylacetate (0.192 g, 1 mmol), the hydrazine (1 mmol), sodium chloride (six times the total mass of reagents) and glacial acetic acid (30 μL, 0.5 mmol). The ball was added and the mixture milled at 30 Hz for 40 minutes. Following this intial grinding period, Selectfluor (0.708 g, 2 mmol) and sodium carbonate (0.133 g, 1.25 mmol) were directly added to the reaction mixture. The jar was hand sealed and milled for a further 60 minutes at 30 Hz. The resulting powder was transferred into a flask, washing the residue with dichloromethane (about 40 mL). The insoluble material was removed by filtration. The solvent was removed under reduced pressure to yield the crude product. This was purified by flash column chromatography on silica gel (gradient elution EtOAc (0–5%) in petroleum ether).

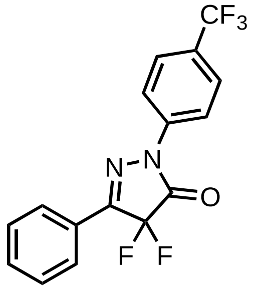

4,4-Difluoro-5-phenyl-2-(4-(trifluoromethyl)phenyl)-2,4-dihydro-3*H*-pyrazol-3-one, 7

Prepared according to **GP2;** 0.127 g, 0.37 mmol, 37%. Yellow powder.

^{1}H NMR (400 MHz; $CDCl_3$): δ 8.13 (d, *J* = 6.8 Hz, 2H), 8.00 (d, *J* = 6.0 Hz, 2H), 7.74 (d, *J* = 6.8 Hz, 2H), 7.58 (m, 3H).

^{19}F NMR (471 MHz; $CDCl_3$): δ -62.3 (3F), -115.0 (2F).

^{13}C NMR (126 MHz; $CDCl_3$): δ 159.5 (t, *J* = 30 Hz), 150.3 (t, *J* = 21 Hz), 139.5, 132.8, 129.5, 128.2 (q, *J* = 33 Hz), 127.0, 126.6 (q, *J* = 4 Hz), 126.0, 123.0 (q, *J* = 272 Hz), 118.4, 109.0 (t, *J* = 259 Hz).

IR: 1751, 1618, 1518, 1406, 1319, 1159, 1109, 1061, 841, 739, 687, 635, 592 cm^{-1}.

HRMS (ASAP+): $[C_{16}H_9N_2F_5O + H]^+$ calc. 341.0713, found 341.0706.

mp: 84-85 °C (ethyl acetate).

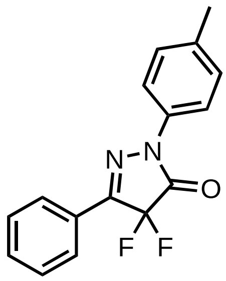

4,4-Difluoro-5-phenyl-2-(*p*-tolyl)-2,4-dihydro-3*H*-pyrazol-3-one, 8

Prepared according to **GP3**; 0.169 g, 0.59 mmol, 59%. Brown powder.

^{1}H NMR (400 MHz; $CDCl_3$): δ 8.00 (d, *J* = 6.0 Hz, 2H), 7.84 (d, *J* = 6.4 Hz, 2H), 7.56 (m, 3H), 7.29 (m, 2H), 2.42 (s, 3H).

^{19}F NMR (471 MHz; $CDCl_3$): δ -115.8.

^{13}C NMR (126 MHz; $CDCl_3$): δ 159.2 (t, *J* = 30 Hz), 149.7 (t, *J* = 21 Hz), 136.5, 135.2, 134.4, 132.3, 129.8, 129.3, 126.8, 118.9, 109.2 (t, *J* = 259 Hz), 21.2.

IR: 2980, 1754, 1512, 1402, 1256, 1067, 937, 816, 739, 664, 640, 598, 513 cm^{-1}.

HRMS (pNSI+): $[C_{16}H_{12}F_2N_2O + H]^+$ calc. 287.0990, found 287.0992.

mp: 80-81 °C (ethyl acetate).

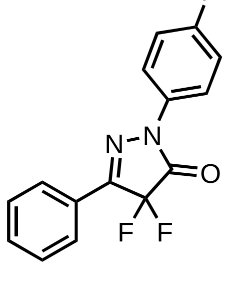

4,4-Difluoro-2-(4-fluorophenyl)-5-phenyl-2,4-dihydro-3*H*-pyrazol-3-one, 9

Prepared according to **GP3;** 0.193 g, 0.67 mmol., 67%. Orange powder.

^{1}H NMR (400 MHz; $CDCl_3$): δ 7.97 - 7.92 (m, 4H), 7.56-7.51 (m, 3H), 7.19-7.14 (m, 2H).

^{19}F NMR (376 MHz; $CDCl_3$): δ -114.8 (1F), -115.6 (2F).

^{13}C NMR (101 MHz; $CDCl_3$): δ 160.8 (d, *J* = 248 Hz), 159.1 (t, *J* = 30 Hz), 150.0 (t, *J* = 21 Hz), 132.9, 132.5, 129.4, 126.9, 126.3 (t, *J* = 2 Hz), 120.7 (d, *J* = 8 Hz), 116.1 (d, *J* = 23 Hz), 109.2 (t, *J* = 260 Hz).

IR: 2980, 1742, 1508, 1225, 1107, 1067, 831, 737, 687 cm^{-1}.

HRMS (ASAP+): $[C_{15}H_9N_2OF_3 + H]^+$ calc. 291.0745, found 291.0745.

mp: 92-93 °C (ethyl acetate).

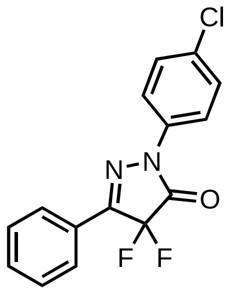

2-(4-Chlorophenyl)-4,4-difluoro-5-phenyl-2,4-dihydro-3*H*-pyrazol-3-one, 10

Prepared according to **GP3;**

0.205 g, 0.67 mmol, 67%. Yellow powder.

^{1}H NMR (400 MHz; $CDCl_3$): δ 7.97 (d, *J* = 7.2 Hz, 2H), 7.91 (m, 2H), 7.53 (m, 3H), 7.44 (m, 2H).

^{19}F NMR (376 MHz; $CDCl_3$): δ -115.3.

^{13}C NMR (101 MHz; $CDCl_3$): δ 159.2 (t, *J* = 30 Hz), 150.1 (t, *J* = 21 Hz), 135.4, 132.6, 131.8, 129.40, 129.37, 126.9, 126.2 (t, *J* = 2 Hz), 119.9, 109.1 (t, *J* = 260 Hz).

IR: 1742, 1493, 1402, 1016, 1111, 737, 687 cm^{-1}.

HRMS (ASAP+): $[C_{15}H_9N_2F_2OCl + H]^+$ calc. 307.0450, found 307.0459.

mp: 79-80 °C (ethyl acetate).

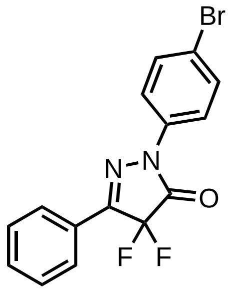

2-(4-Bromophenyl)-4,4-difluoro-5-phenyl-2,4-dihydro-3*H*-pyrazol-3-one, 11

Prepared according to **GP3**; 0.246 g, 0.70 mmol, 70%. Orange powder.

^{1}H NMR (400 MHz; $CDCl_3$): δ 7.98 (d, *J* = 6.8 Hz, 2H), 7.87 (d, *J* = 9.2 Hz, 2H), 7.58-7.52 (m, 5H).

^{19}F NMR (376 MHz; $CDCl_3$): δ -115.3.

^{13}C NMR (101 MHz; $CDCl_3$): δ 159.1 (t, *J* = 30 Hz), 150.1 (t, *J* = 21 Hz), 135.8, 132.6, 132.3, 129.4, 126.9, 126.1 (t, *J* = 2 Hz), 120.1, 119.6, 109.1 (t, *J* = 260 Hz).

IR: 1745, 1489, 1400, 1254, 1103, 739, 683 cm^{-1}.

HRMS (ASAP+): $[C_{15}H_9N_2F_2OBr + H]^+$ calc. 350.9945, found 350.9930.

mp: 79-80 °C (ethyl acetate)

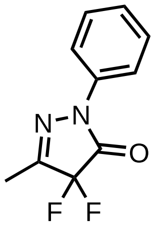

4,4-Difluoro-5-methyl-2-phenyl-2,4-dihydro-3*H*-pyrazol-3-one, 12[7]

Prepared according to **GP2**. 0.064 g, 0.30 mmol, 30%. Red oil.

^{1}H NMR (400 MHz; $CDCl_3$): δ 7.86 (d, *J* = 7.8 Hz, 2H), 7.48-7.44 (m, 2H), 7.28 (t, *J* = 7.4 Hz, 1H), 2.32 (t, *J* = 1.3 Hz, 3H).

^{19}F NMR (376 MHz; $CDCl_3$): δ -122.4.

^{13}C NMR (101 MHz; $CDCl_3$): δ 159.2 (t, *J* = 30 Hz), 152.0 (t, *J* = 23 Hz), 136.7, 129.2, 126.3, 118.6, 108.1 (t, *J* = 258 Hz), 11.9.

IR: 2980, 1748, 1501, 1373, 1254, 1146, 1111, 754, 731, 689, 648 cm^{-1}.

HRMS (ASAP+): $[C_{10}H_8N_2OF_2 + H]^+$ calc. 211.0683, found 211.0687.

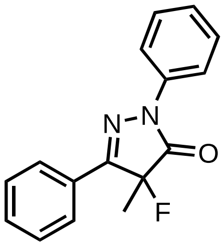

4-Fluoro-4-methyl-2,5-diphenyl-2,4-dihydro-3*H*-pyrazol-3-one, 13

Prepared according to modified **GP2** using 1 equivalent of Selectfluor. 0.126 g, 0.47 mmol, 47%. Orange powder.

^{1}H NMR (400 MHz; $CDCl_3$): δ 8.02-7.99 (m, 4H), 7.54-7.53 (m, 3H), 7.52-7.48 (m, 2H), 7.28 (t, *J* = 7.6 Hz, 1H), 1.91 (d, *J* = 22.4 Hz, 3H).

^{19}F NMR (471 MHz; $CDCl_3$): δ -163.8 (q, *J* = 23.1 Hz).

^{13}C NMR (126 MHz; $CDCl_3$): δ 168.9 (d, *J* = 22 Hz), 155.8 (d, *J* = 14 Hz), 137.6, 131.4, 129.20, 129.16, 128.7 (d, *J* = 2 Hz), 126.8 (d, *J* = 2 Hz), 125.9, 119.0, 93.7 (d, *J* = 195 Hz), 21.6 (d, *J* = 27 Hz).

IR: 1724, 1593, 1395, 1177, 1117, 745, 683 cm^{-1}.

HRMS (pNSI+): $[C_{16}H_{13}ON_2F + H]^+$ calc. 269.1085, found 269.1086.

mp: 73-74 °C (ethyl acetate).

Spectroscopic data

β-ketoesters

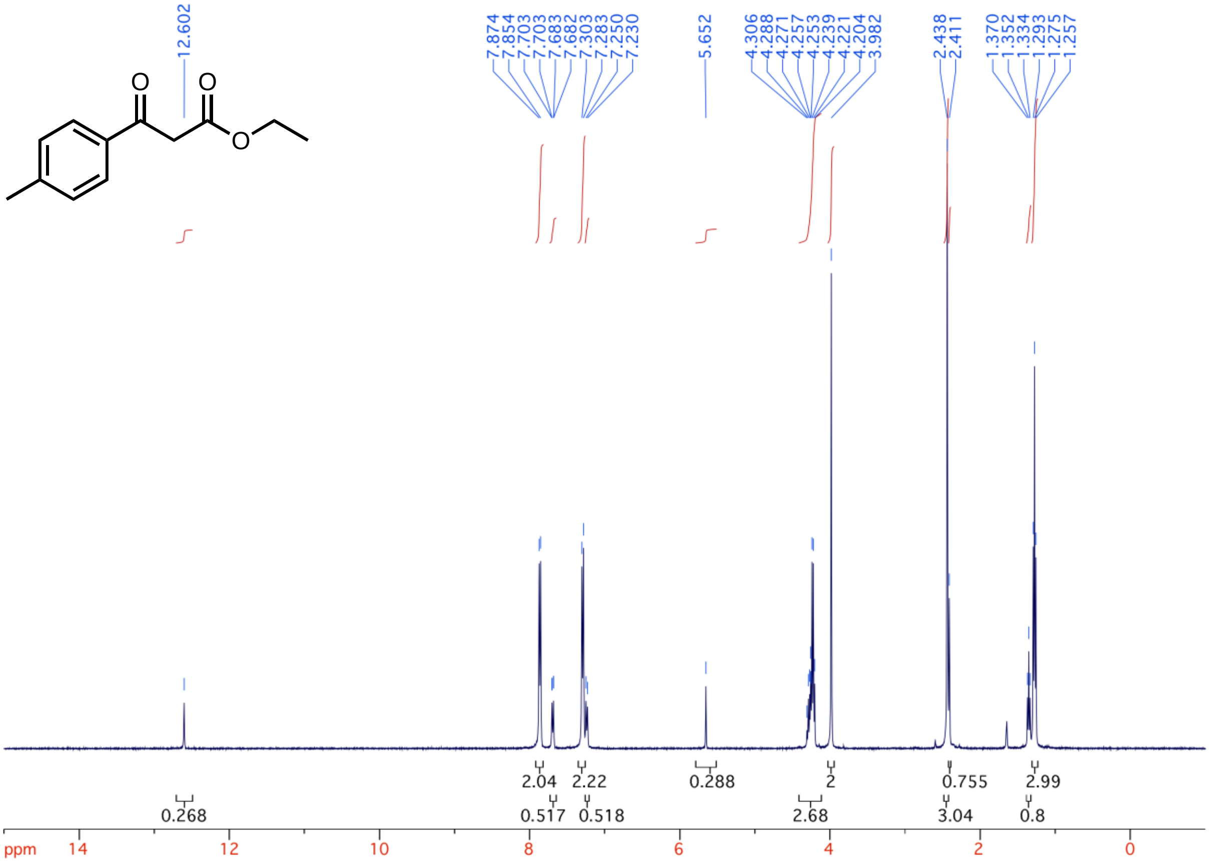

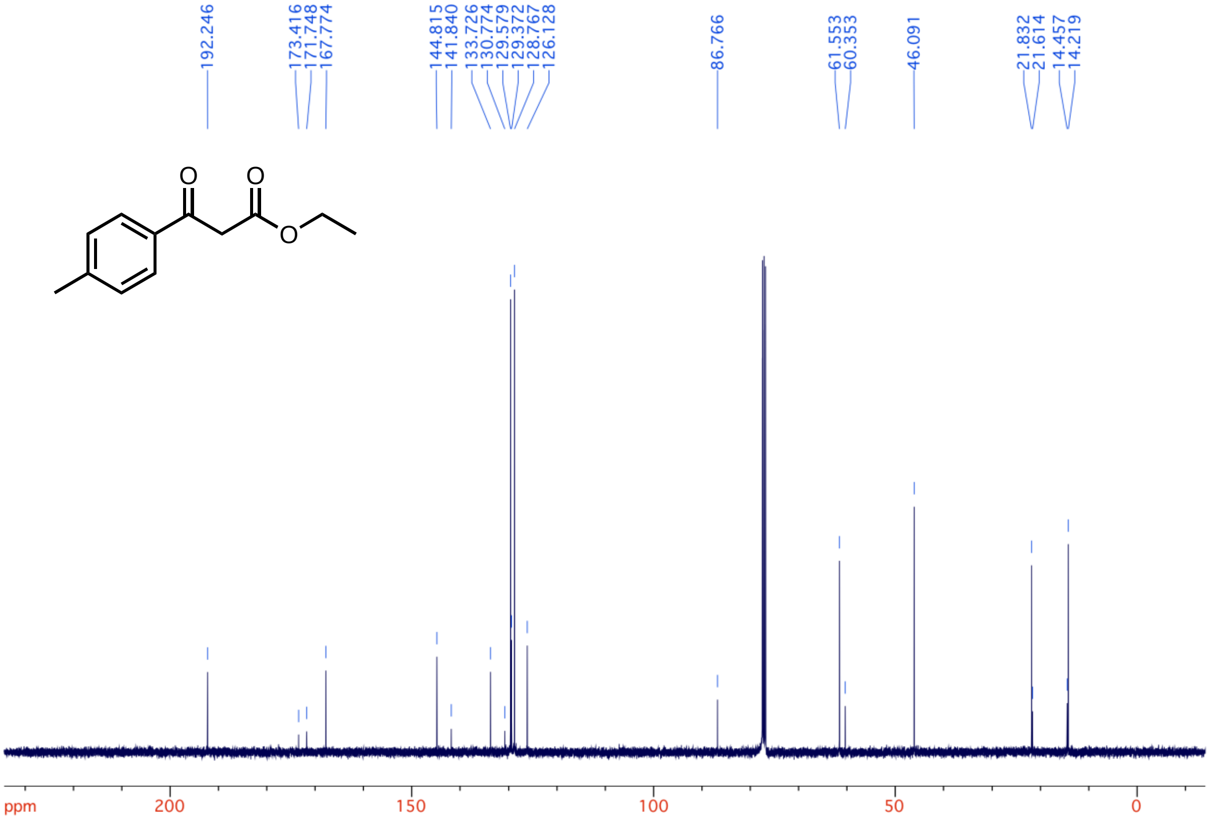

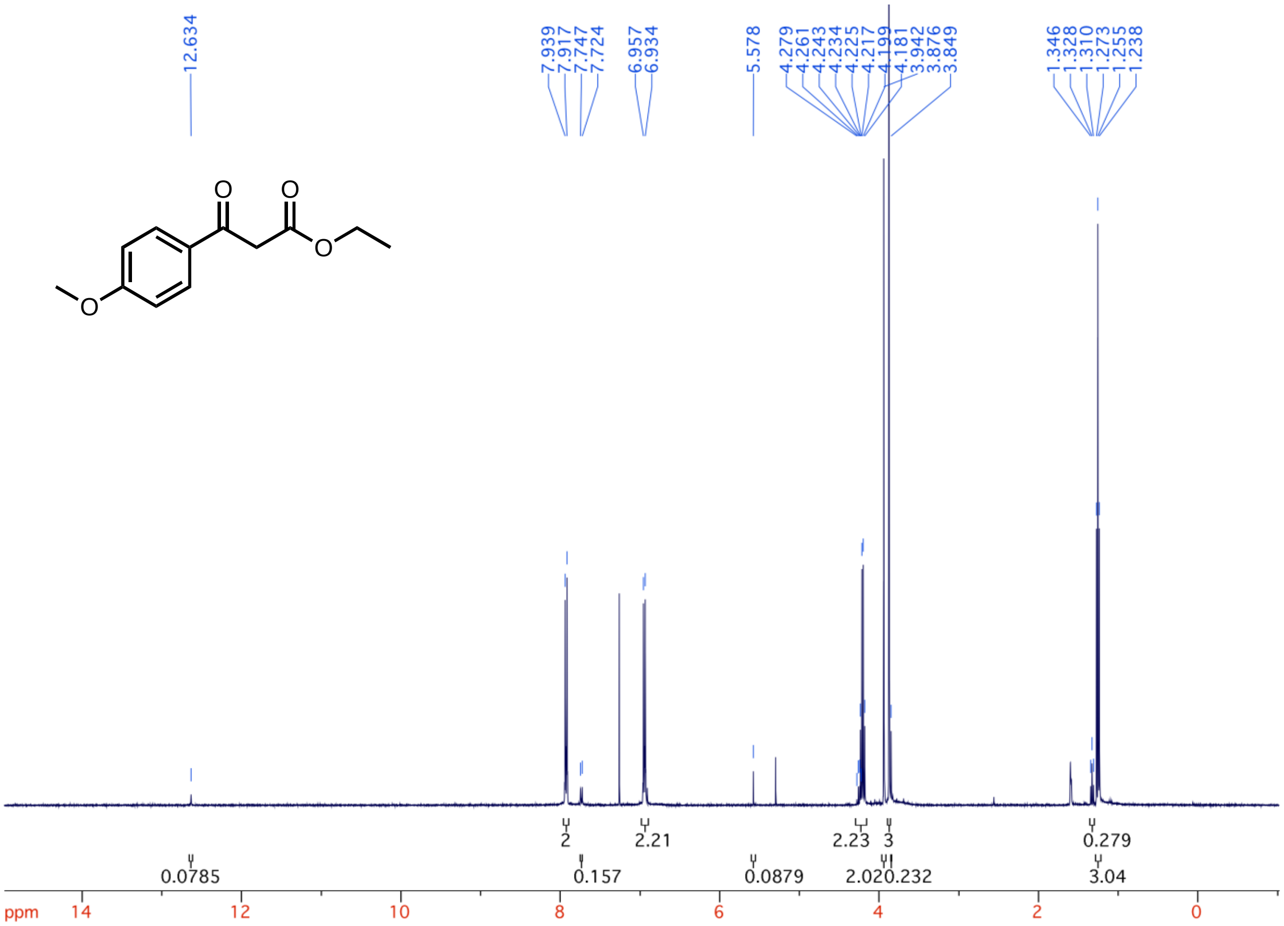
12.634
7.939
7.917
7.747
7.724
6.957
6.934
5.578
4.279
4.261
4.243
4.234
4.225
4.217
4.199
4.181
3.942
3.876
3.849
1.346
1.328
1.310
1.273
1.255
1.238
O
O
O
O
2
2.21
2.23
3
0.279
0.0785
0.157
0.0879
2.02
0.232
3.04
ppm
14
12
10
8
6
4
2
0

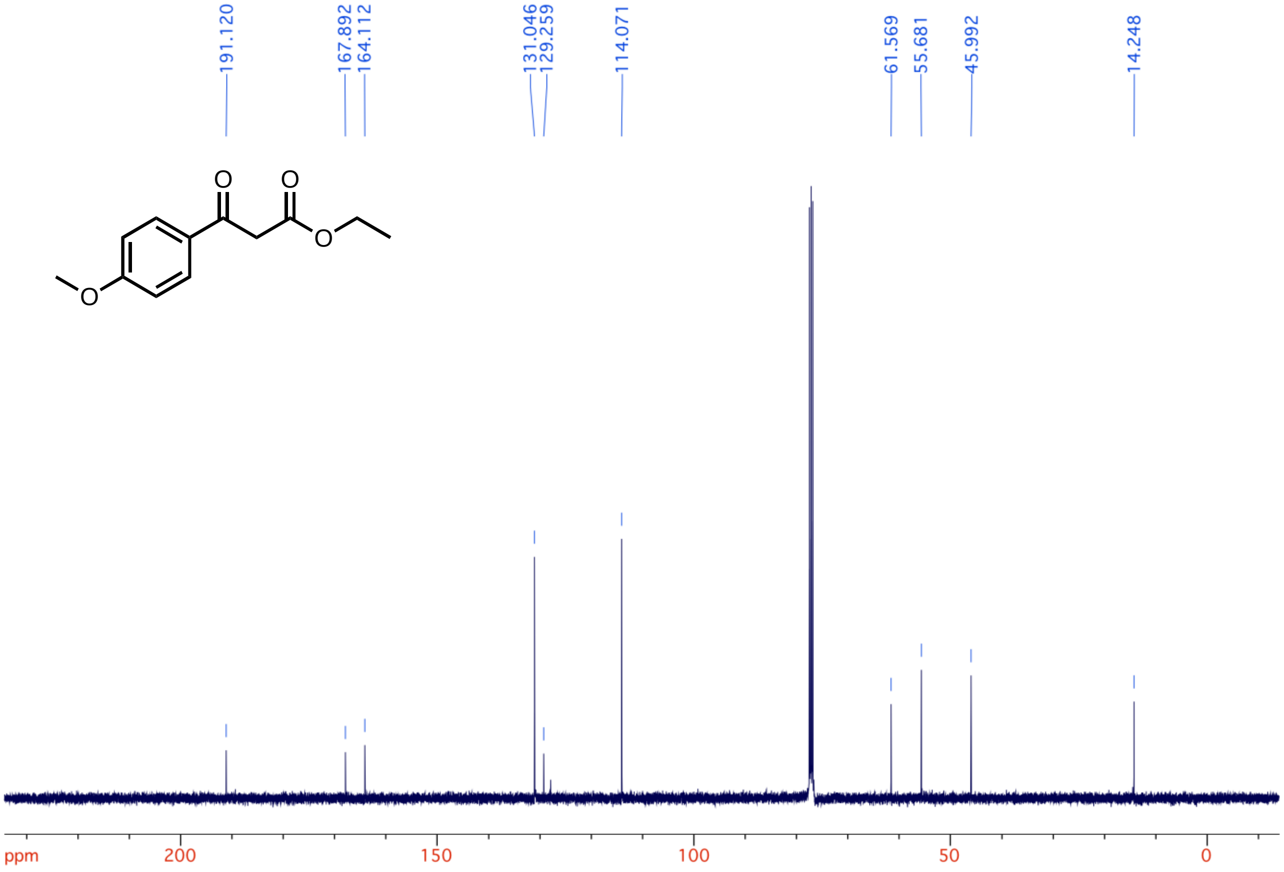
191.120
167.892
164.112
131.046
129.259
114.071
61.569
55.681
45.992
14.248
O
O
O
O
ppm
200
150
100
50
0

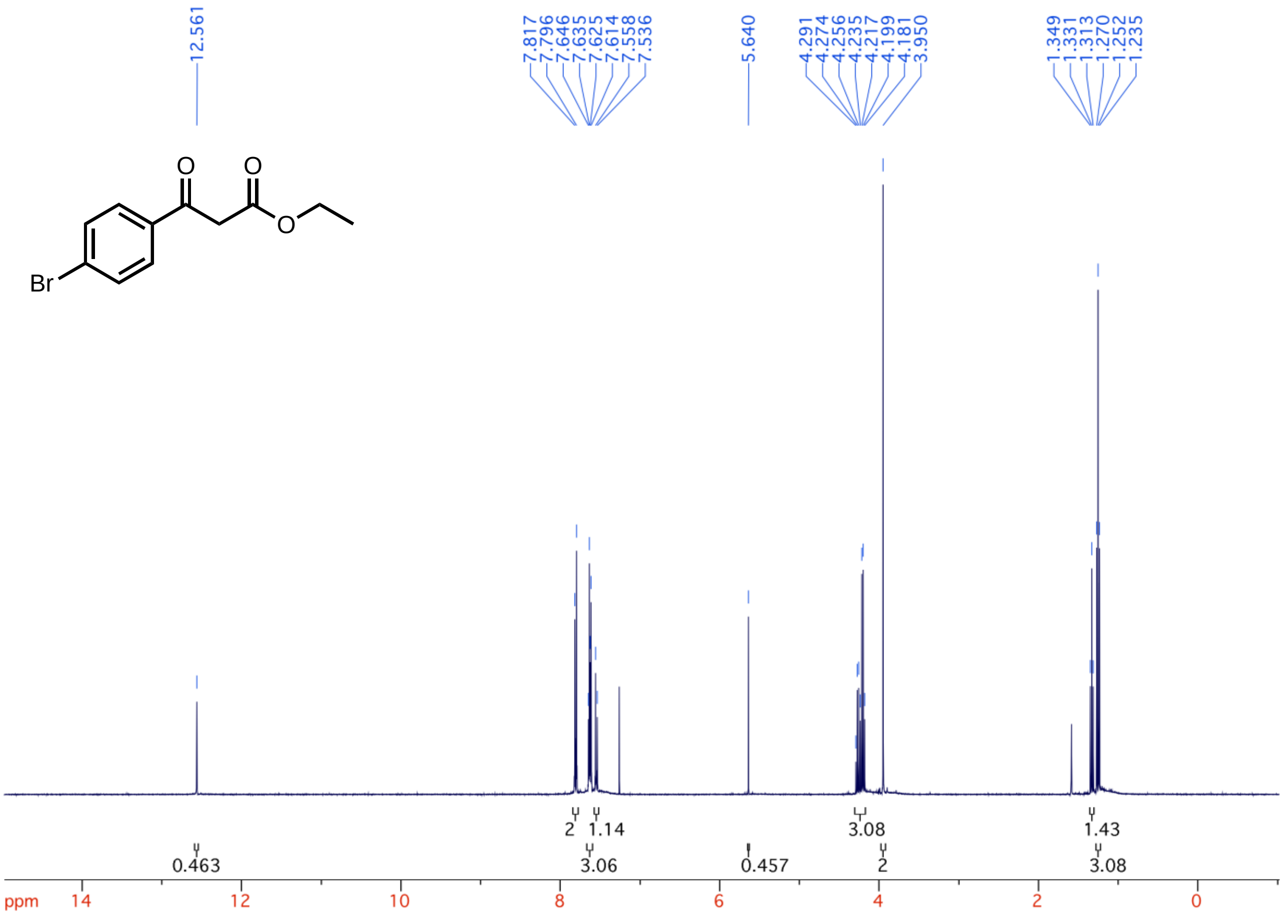
12.561
7.817
7.796
7.646
7.635
7.625
7.614
7.558
7.536
5.640
4.291
4.274
4.256
4.235
4.217
4.199
4.181
3.950
1.349
1.331
1.313
1.270
1.252
1.235
O
O
O
Br
2
1.14
0.463
3.06
0.457
3.08
2
1.43
3.08
ppm
14
12
10
8
6
4
2
0

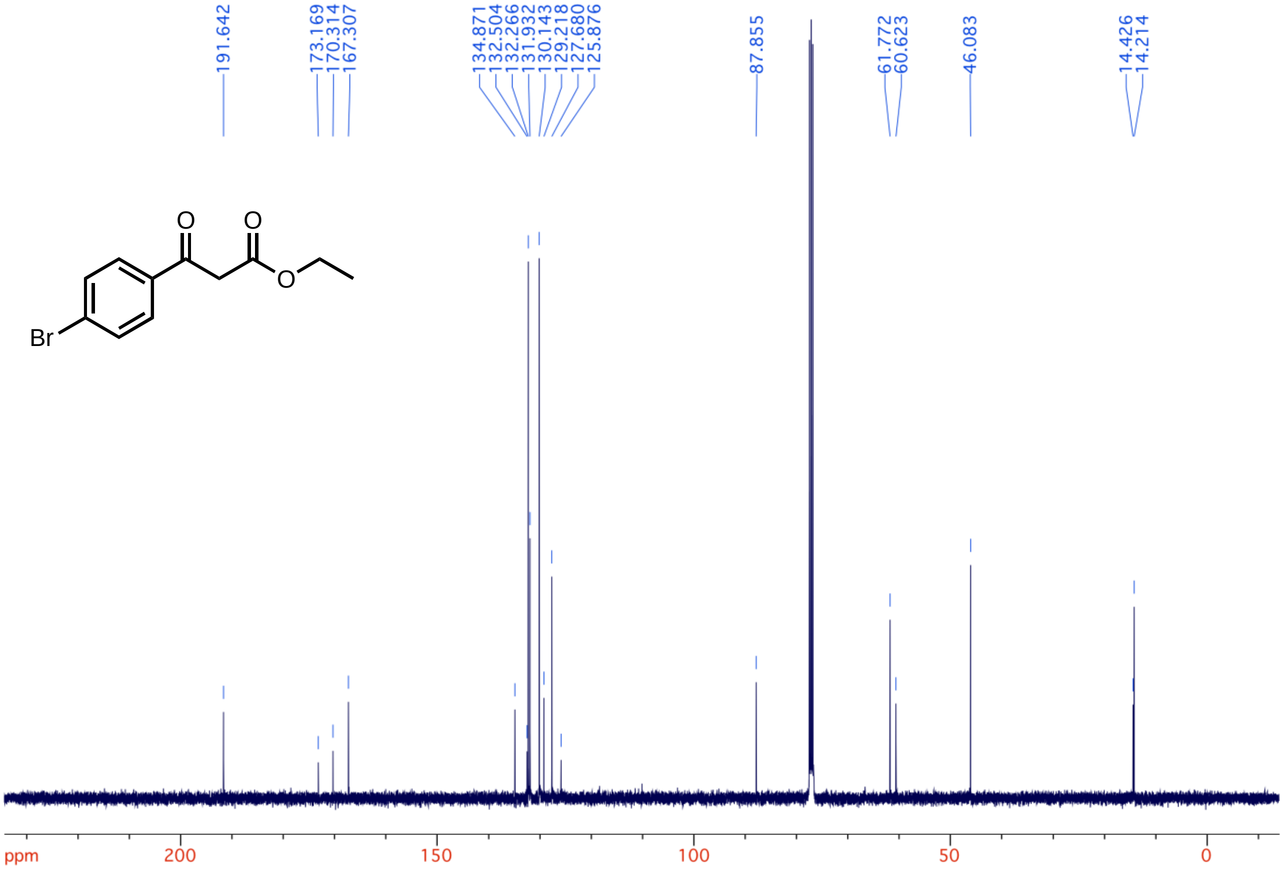
191.642
173.169
170.314
167.307
134.871
132.504
132.266
131.932
130.143
129.218
127.680
125.876
87.855
61.772
60.623
46.083
14.426
14.214
O
O
O
Br
ppm
200
150
100
50
0

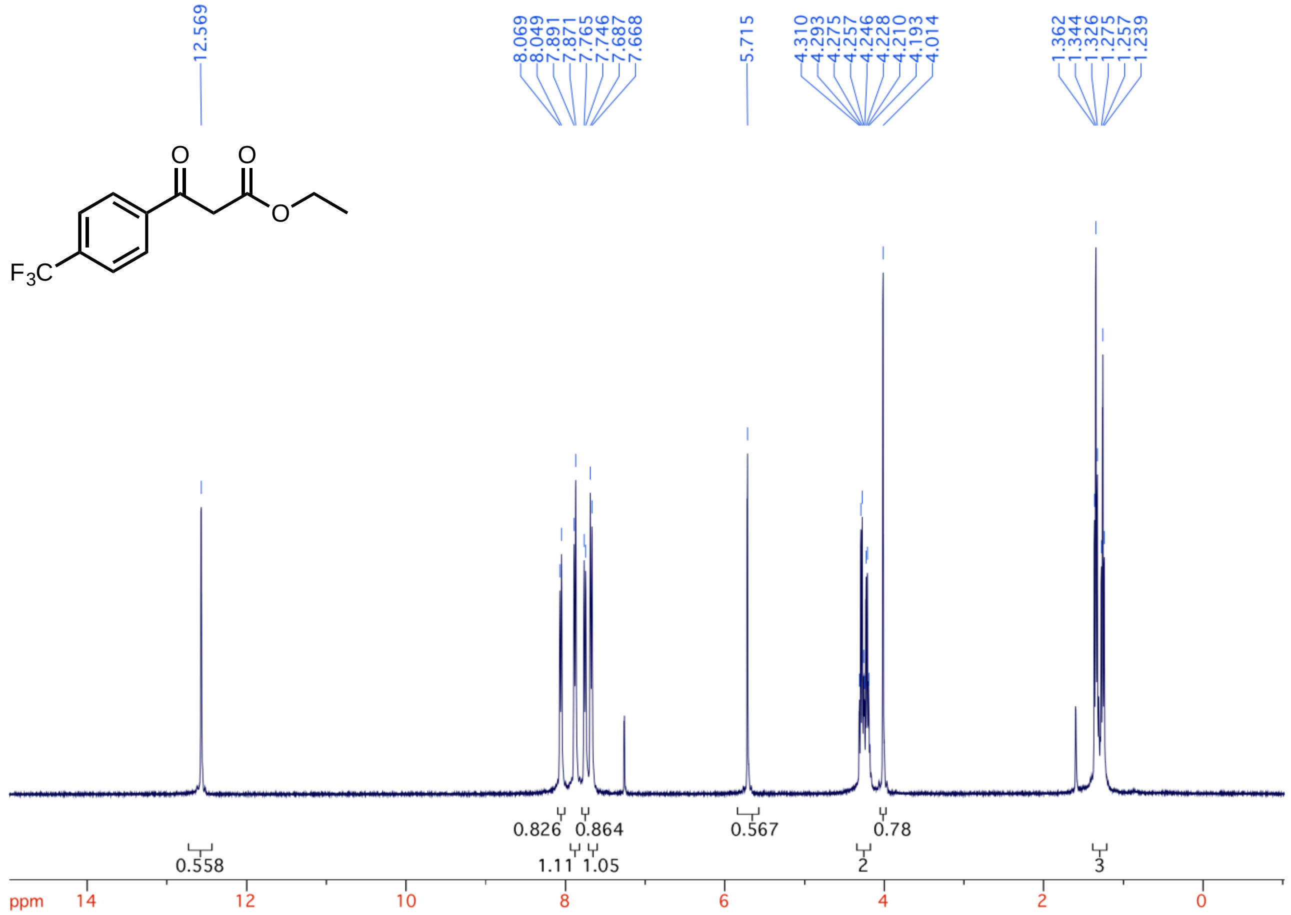

12.569
8.069
8.049
7.891
7.871
7.765
7.746
7.687
7.668
5.715
4.310
4.293
4.275
4.257
4.246
4.228
4.210
4.193
4.014
1.362
1.344
1.326
1.275
1.257
1.239
O
O
O
F3C
0.826
0.864
0.567
0.78
0.558
1.11
1.05
2
3
ppm
14
12
10
8
6
4
2
0

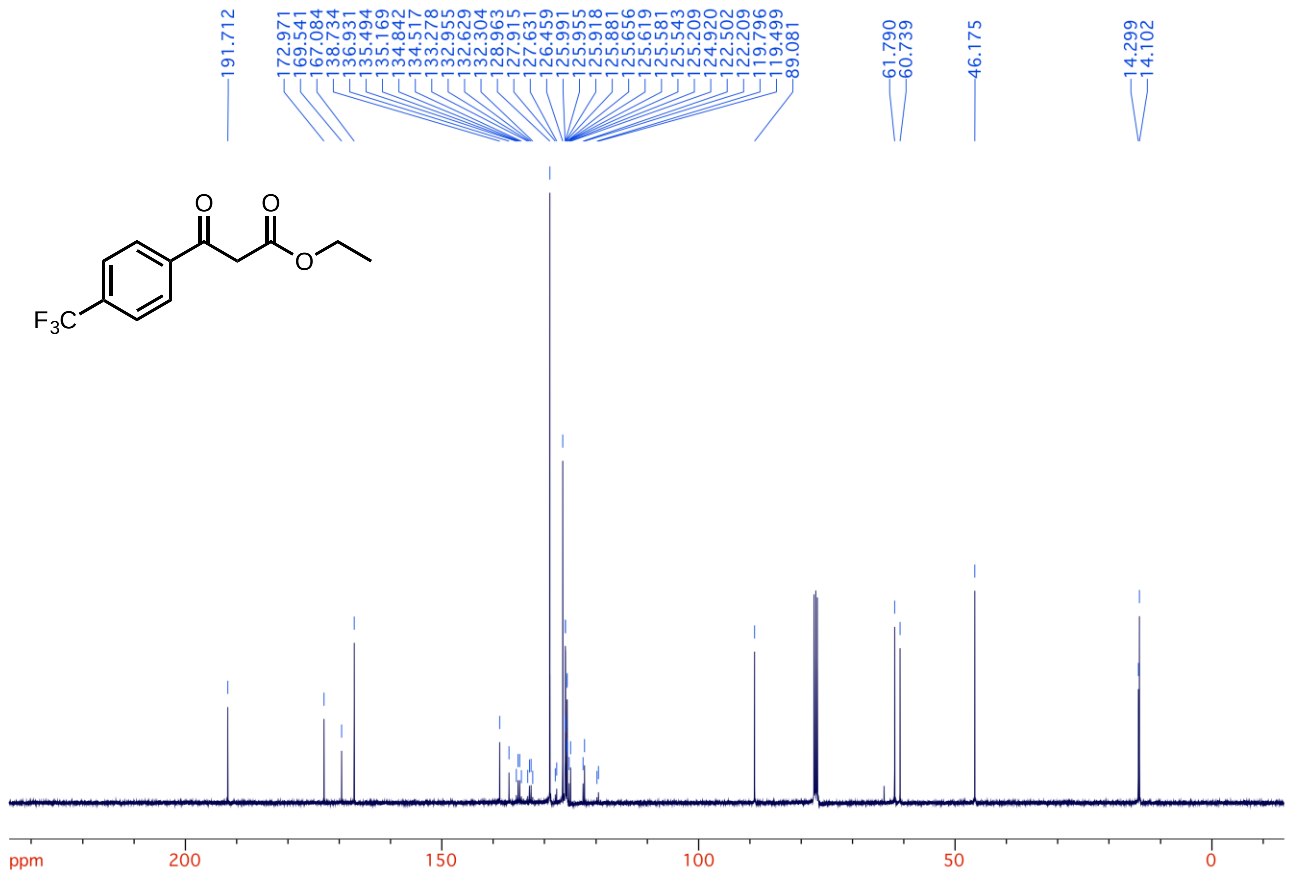

191.712
172.971
169.541
167.084
138.734
136.931
135.494
135.169
134.842
134.517
133.278
132.955
132.629
132.304
128.963
127.915
127.631
126.459
125.991
125.955
125.918
125.881
125.656
125.619
125.581
125.543
125.209
124.920
122.502
122.209
119.796
119.499
89.081
61.790
60.739
46.175
14.299
14.102
O
O
O
F3C
ppm
200
150
100
50
0

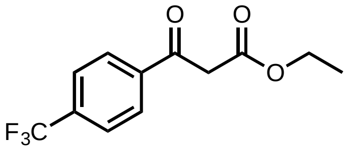
F3C
O
O
O

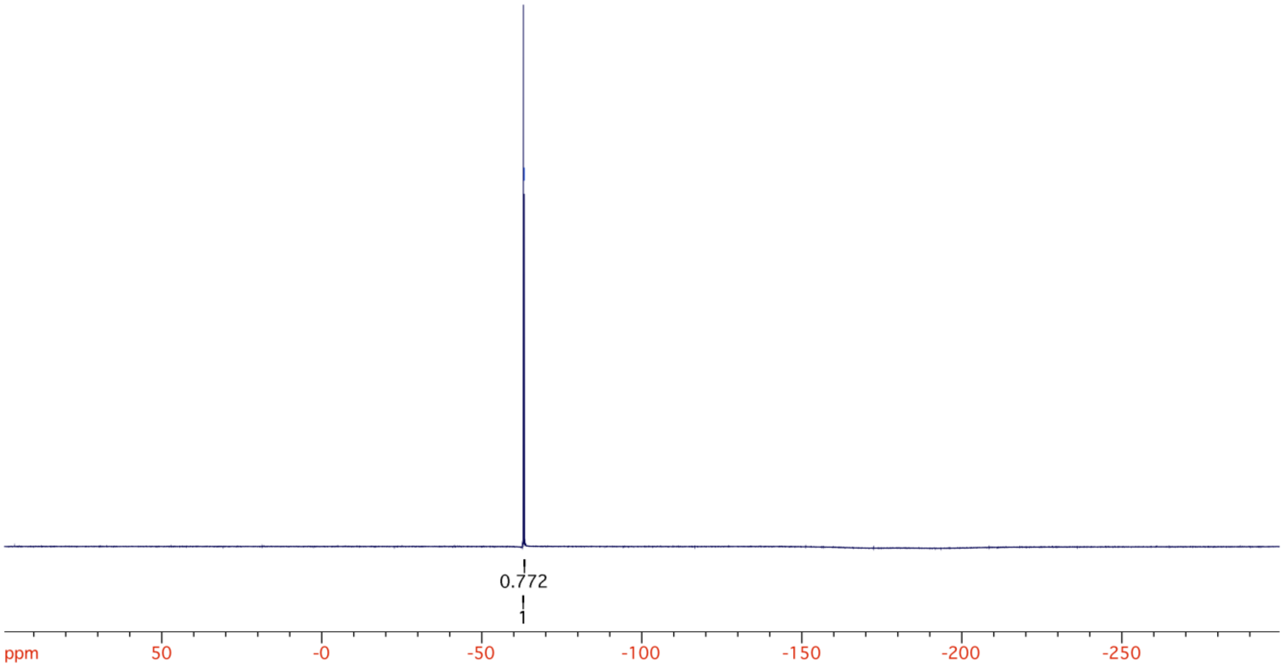
-62.944
-63.221
0.772
ppm
50
-0
-50
-100
-150
-200
-250

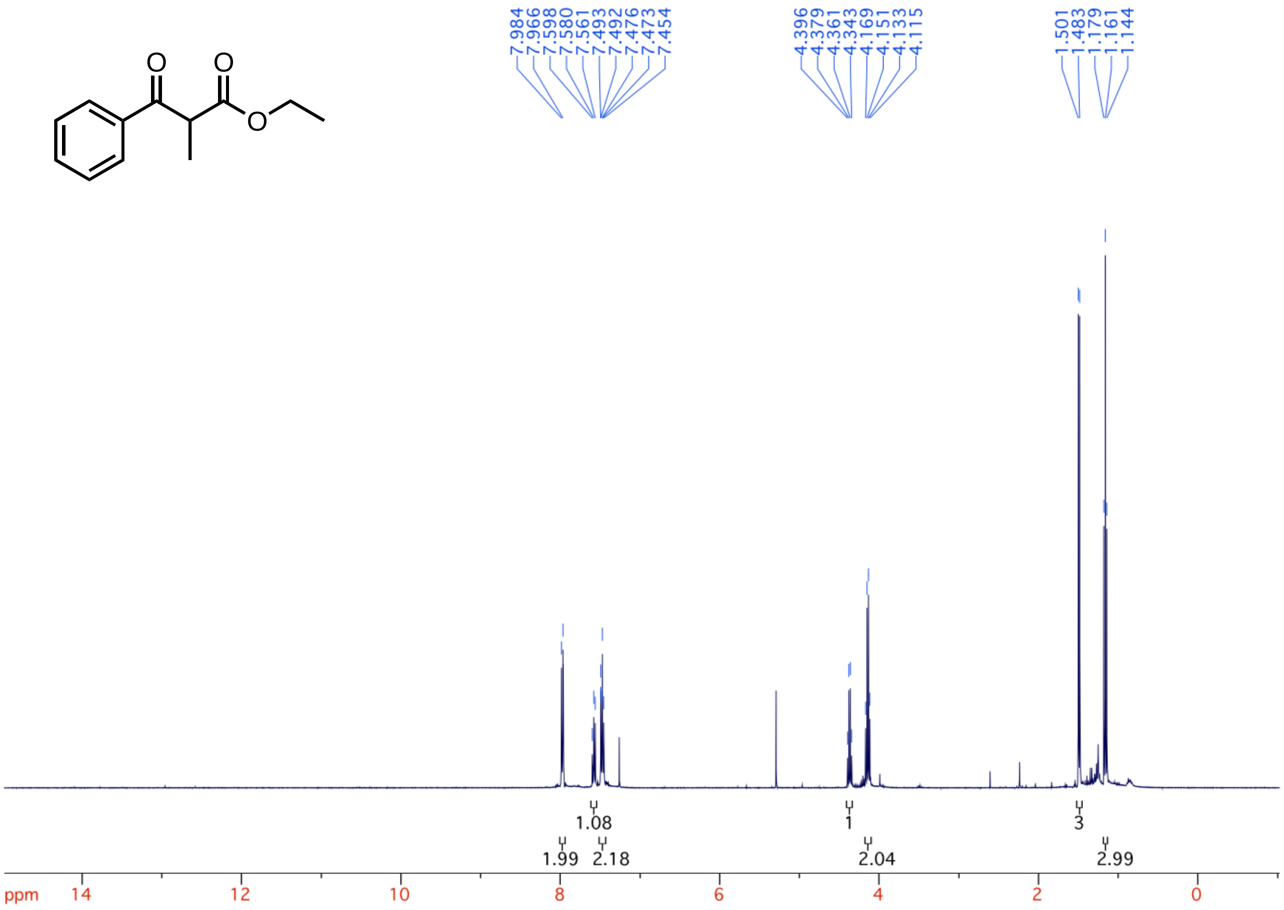
O
O
O
7.984
7.966
7.598
7.580
7.561
7.493
7.492
7.476
7.473
7.454
4.396
4.379
4.361
4.343
4.169
4.151
4.133
4.115
1.501
1.483
1.179
1.161
1.144
1.08
1
3
1.99
2.18
2.04
2.99
ppm
14
12
10
8
6
4
2
0

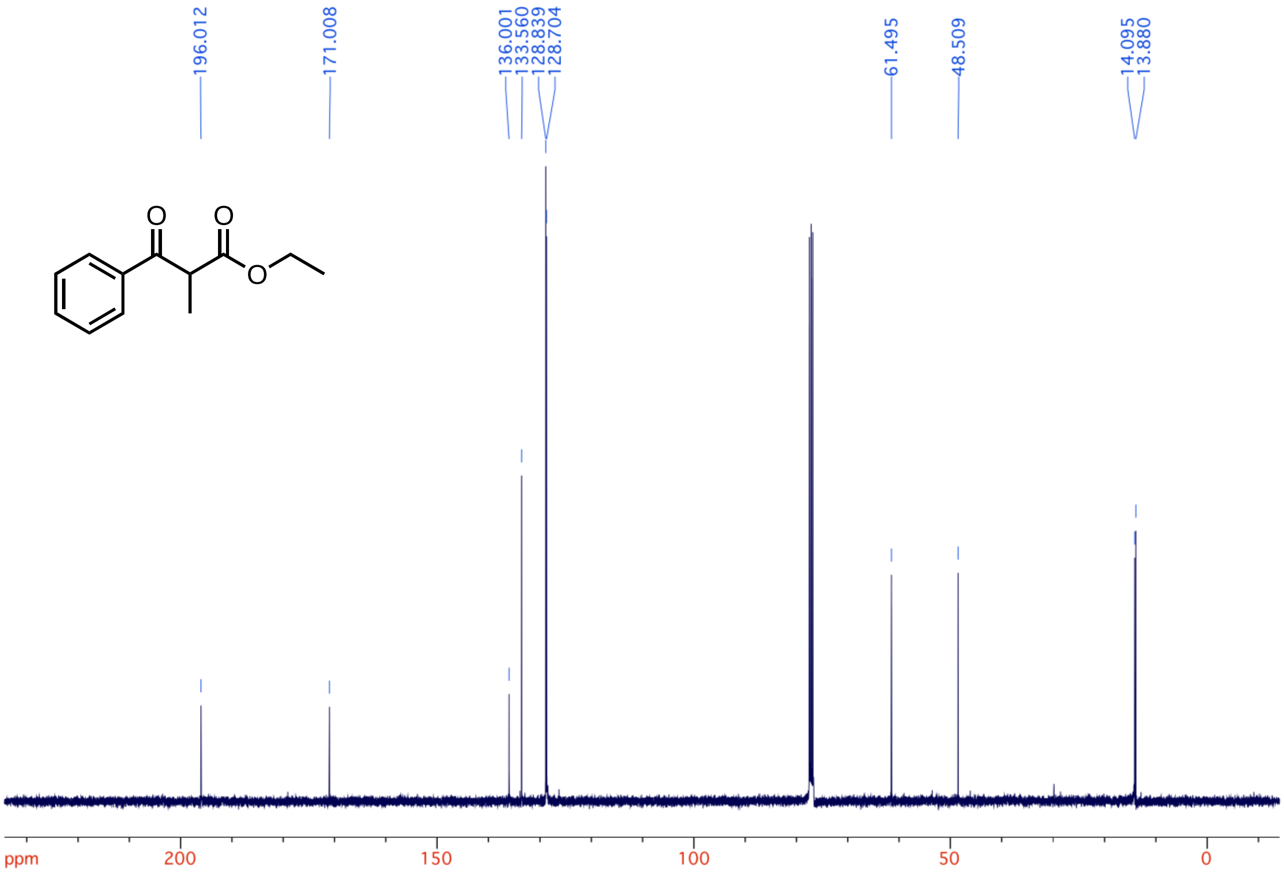
196.012
171.008
136.001
133.560
128.839
128.704
61.495
48.509
14.095
13.880
O
O
O
ppm
200
150
100
50
0

Fluorinated pyrazolones

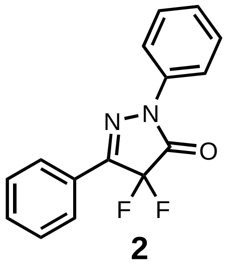

2

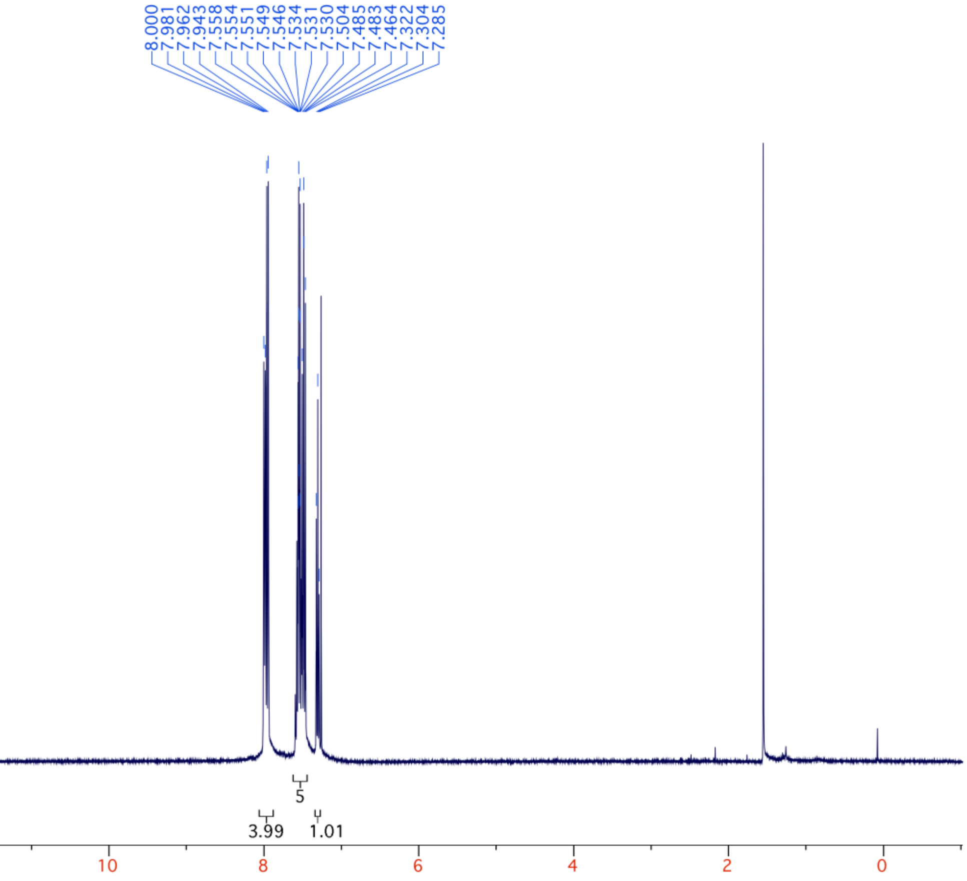

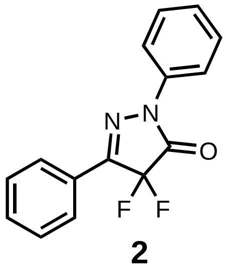

2

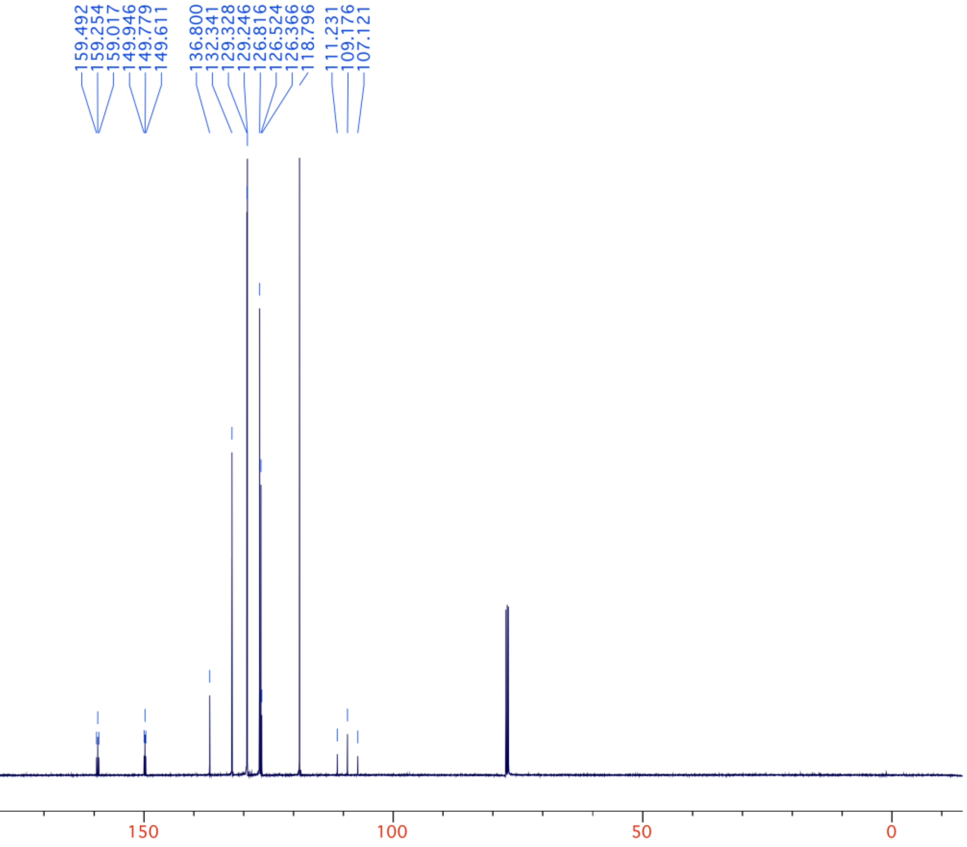

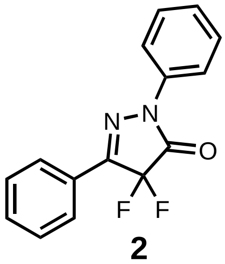

2

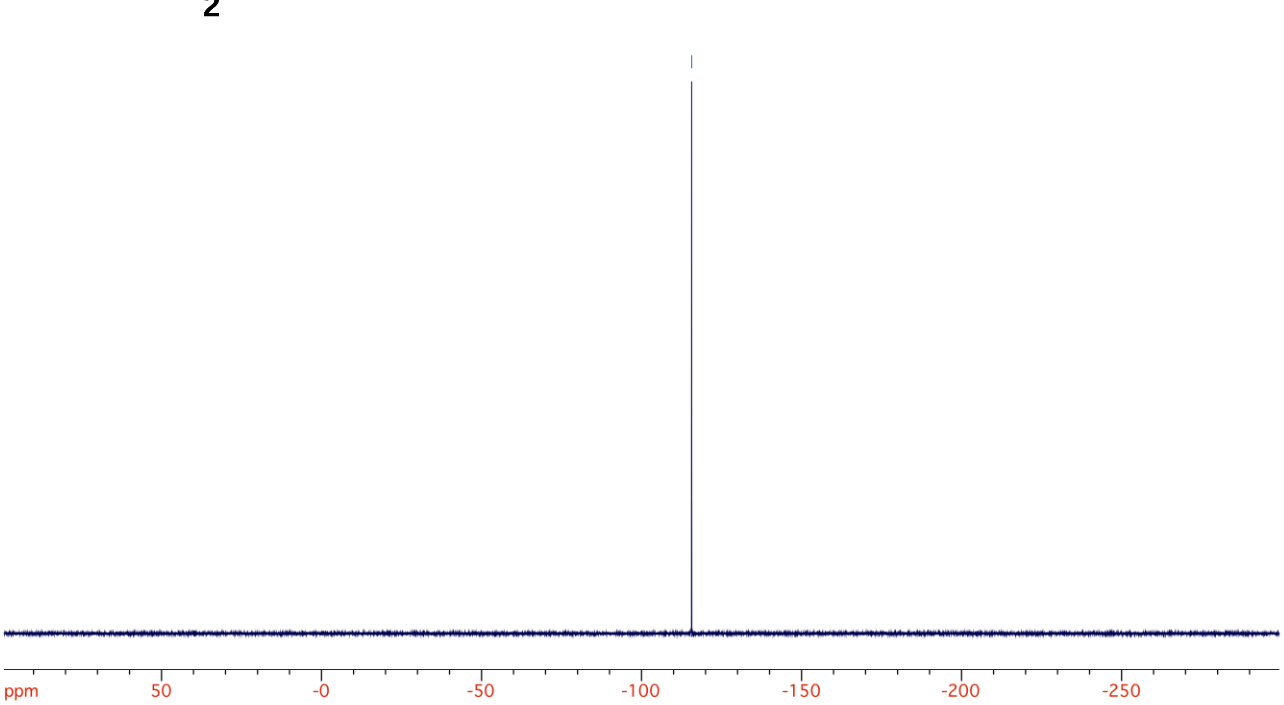

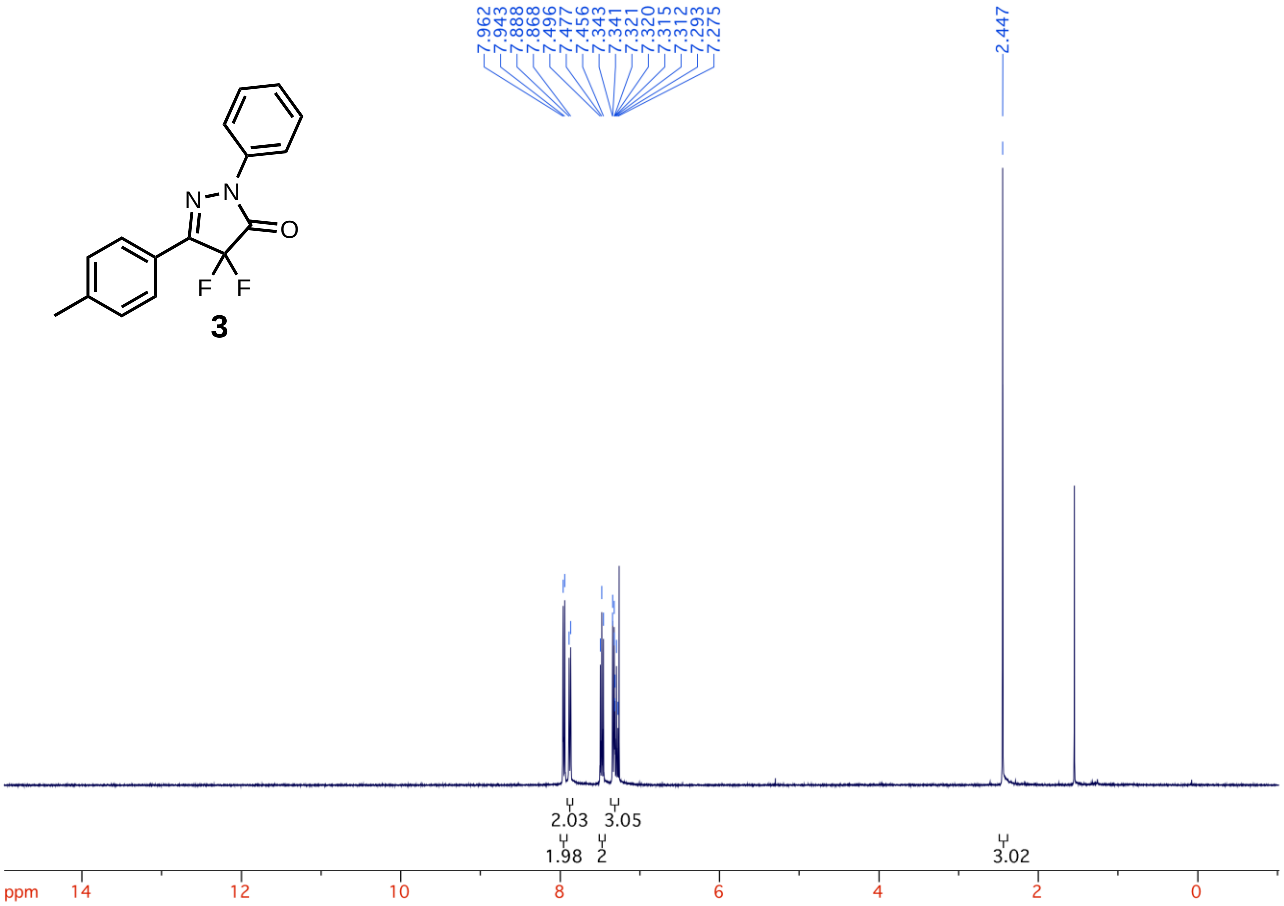
7.962
7.943
7.888
7.868
7.496
7.477
7.456
7.343
7.341
7.321
7.320
7.315
7.312
7.293
7.275
2.447
N
N
O
F
F
3
2.03
3.05
1.98
2
3.02
ppm
14
12
10
8
6
4
2
0

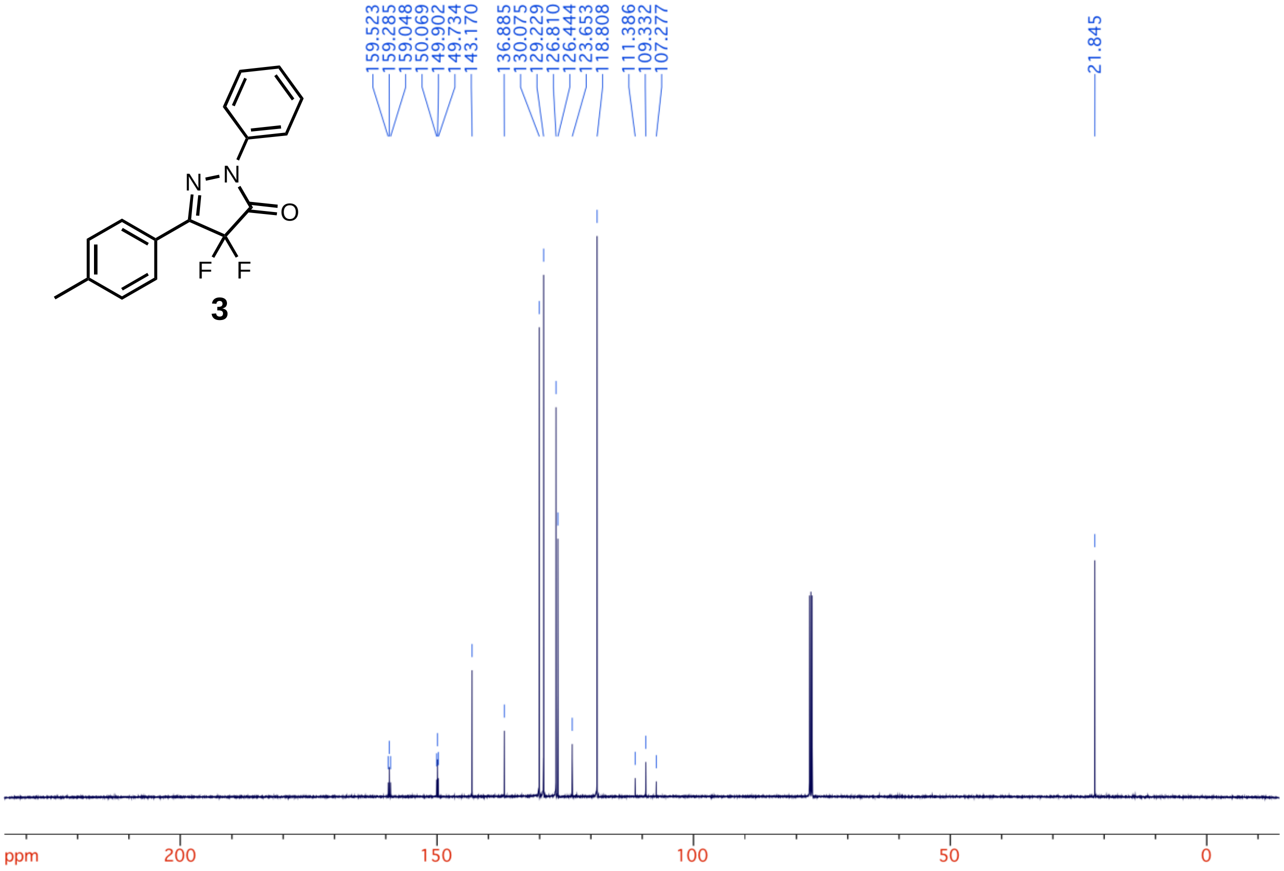
159.523
159.285
159.048
150.069
149.902
149.734
143.170
136.885
130.075
129.229
126.810
126.444
123.653
118.808
111.386
109.332
107.277
21.845
N
N
O
F
F
3
ppm
200
150
100
50
0

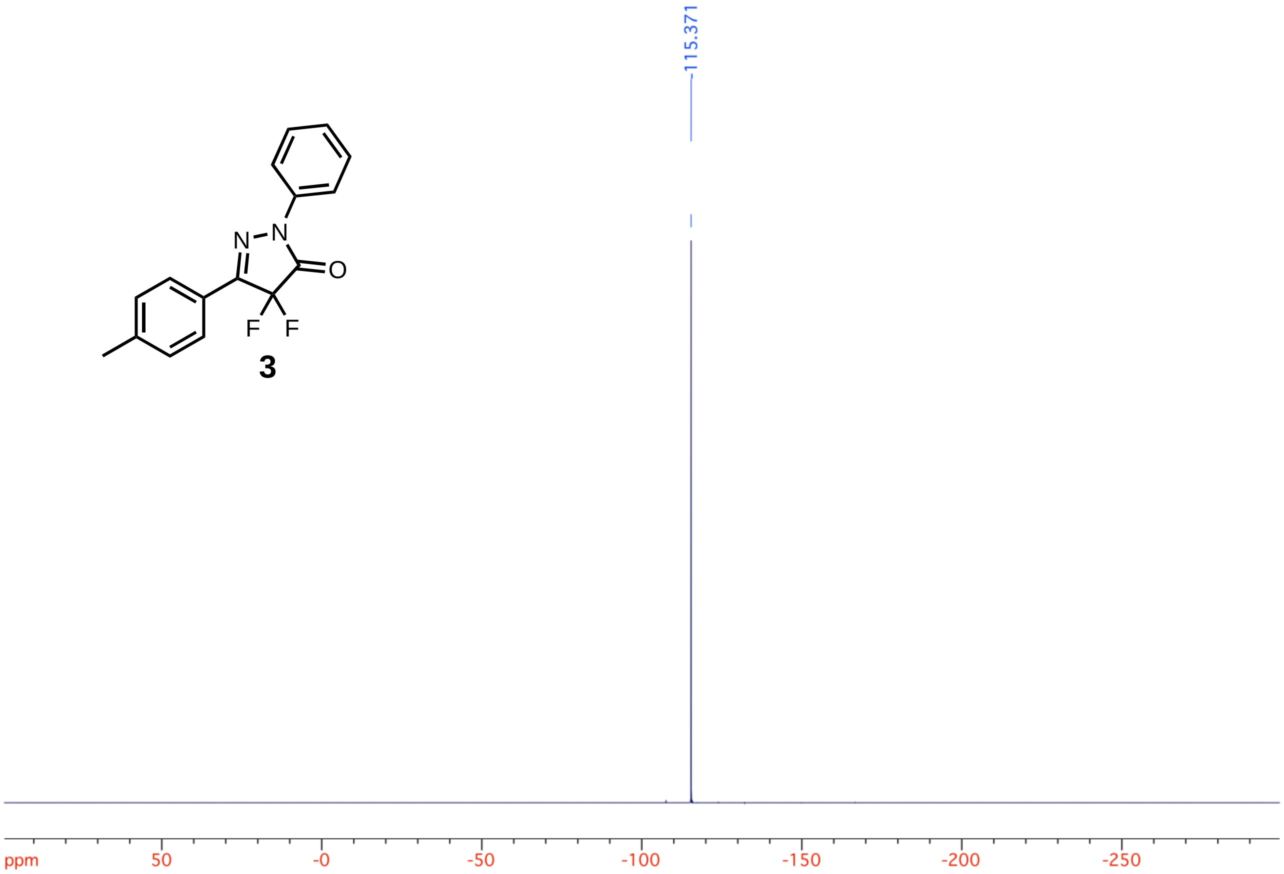

3

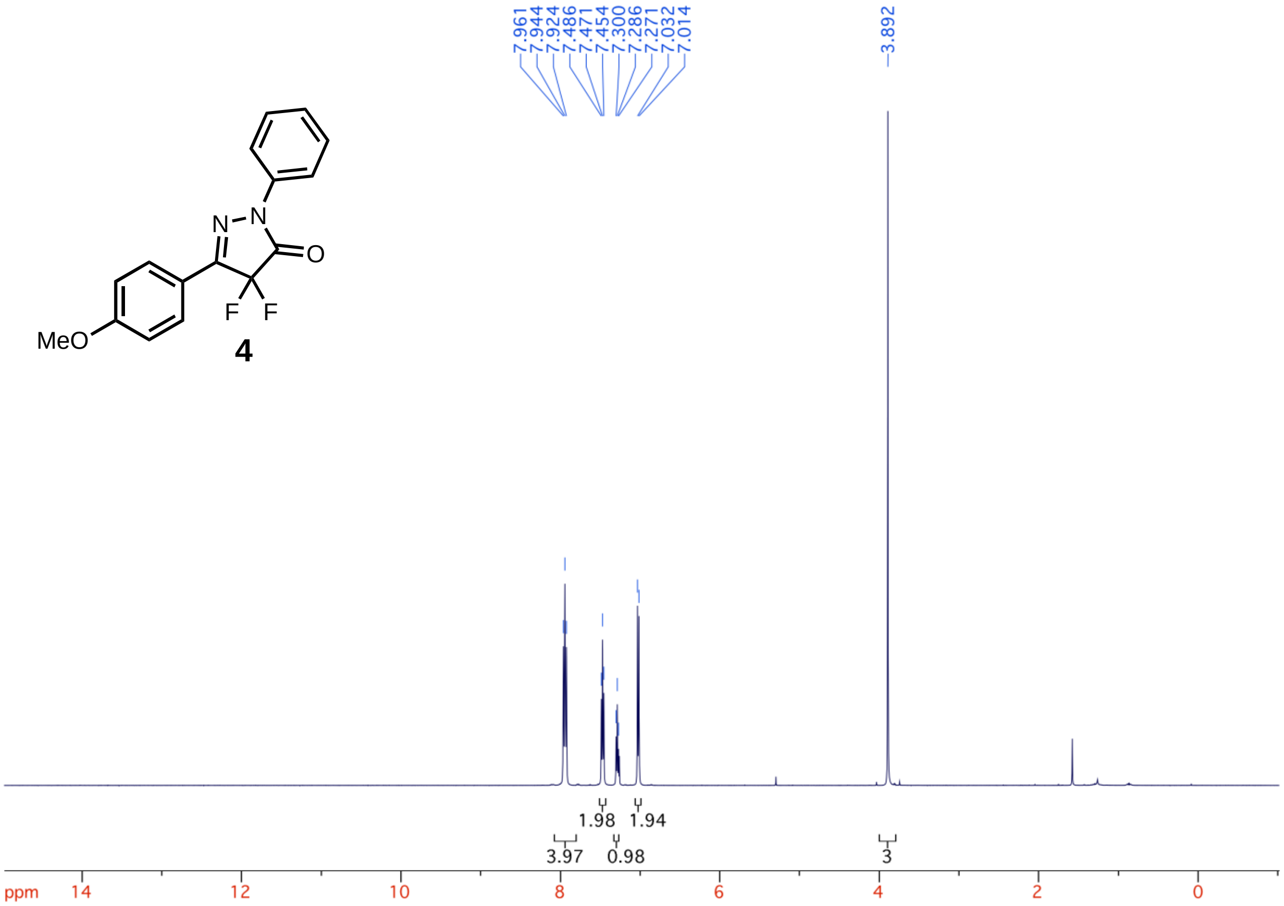
7.961
7.944
7.924
7.486
7.471
7.454
7.300
7.286
7.271
7.032
7.014
3.892
N
N
O
F
F
MeO
4
1.98
1.94
3.97
0.98
3
ppm 14 12 10 8 6 4 2 0

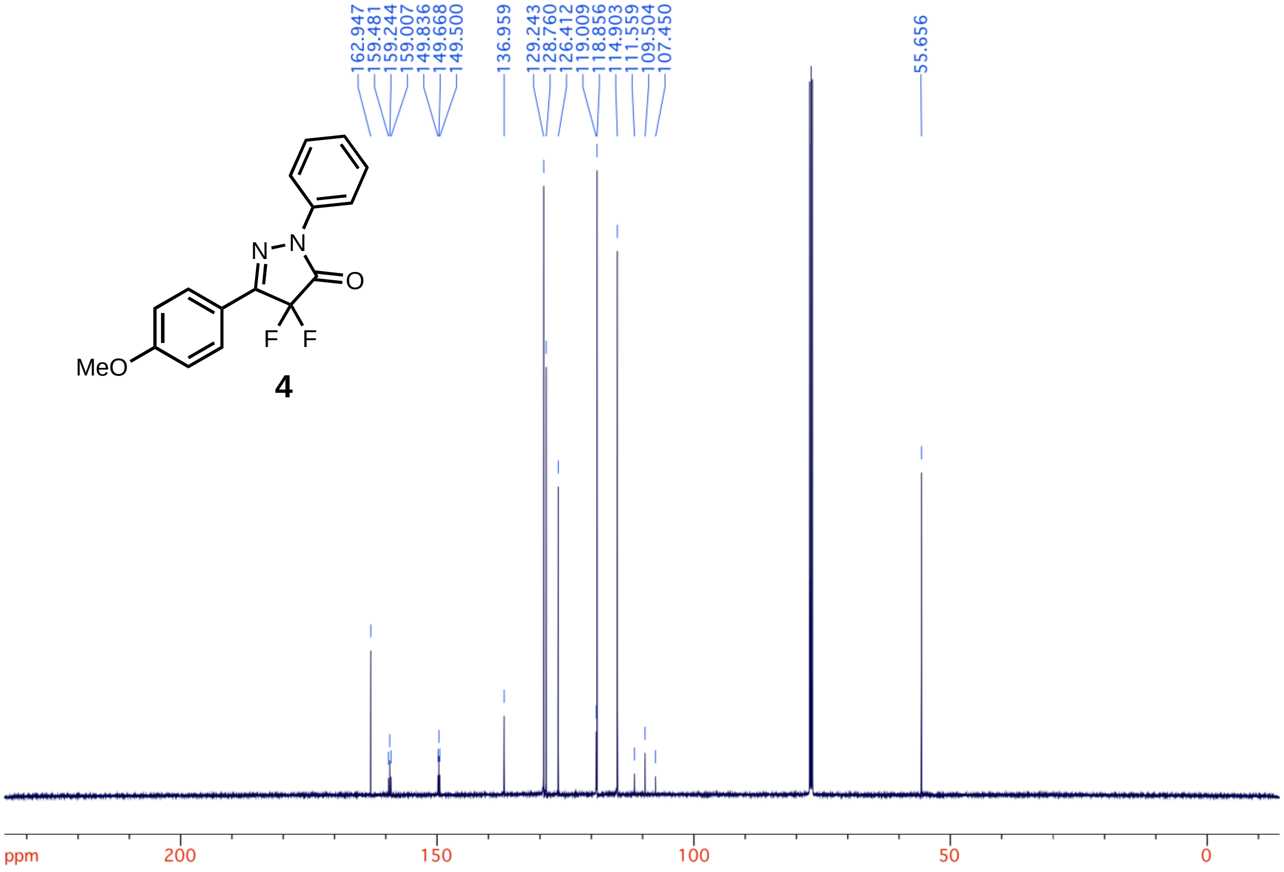
162.947
159.481
159.244
159.007
149.836
149.668
149.500
136.959
129.243
128.760
126.412
119.009
118.856
114.903
111.559
109.504
107.450
55.656
N
N
O
F
F
MeO
4
ppm 200 150 100 50 0

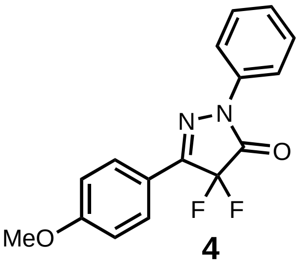

4

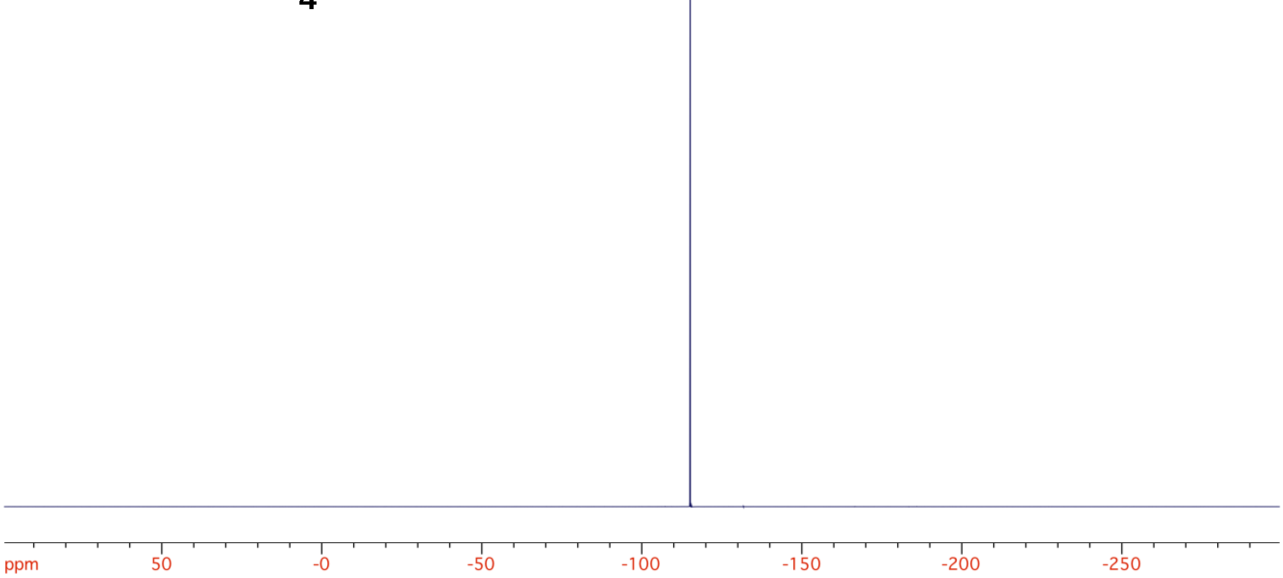

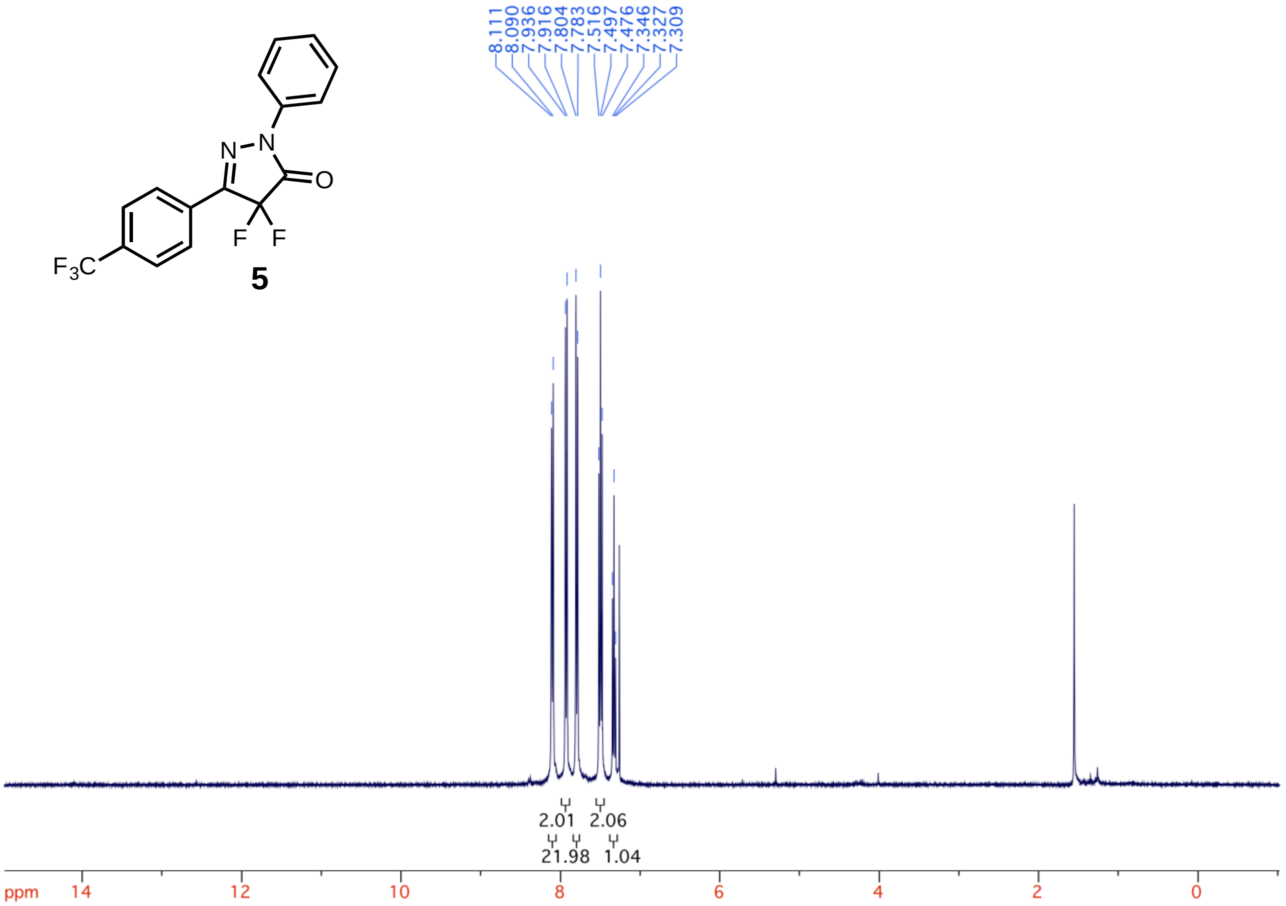
F3C
N
N
O
F F
5
8.111
8.090
7.936
7.916
7.804
7.783
7.516
7.497
7.476
7.346
7.327
7.309
2.01 2.06
21.98 1.04
ppm 14 12 10 8 6 4 2 0

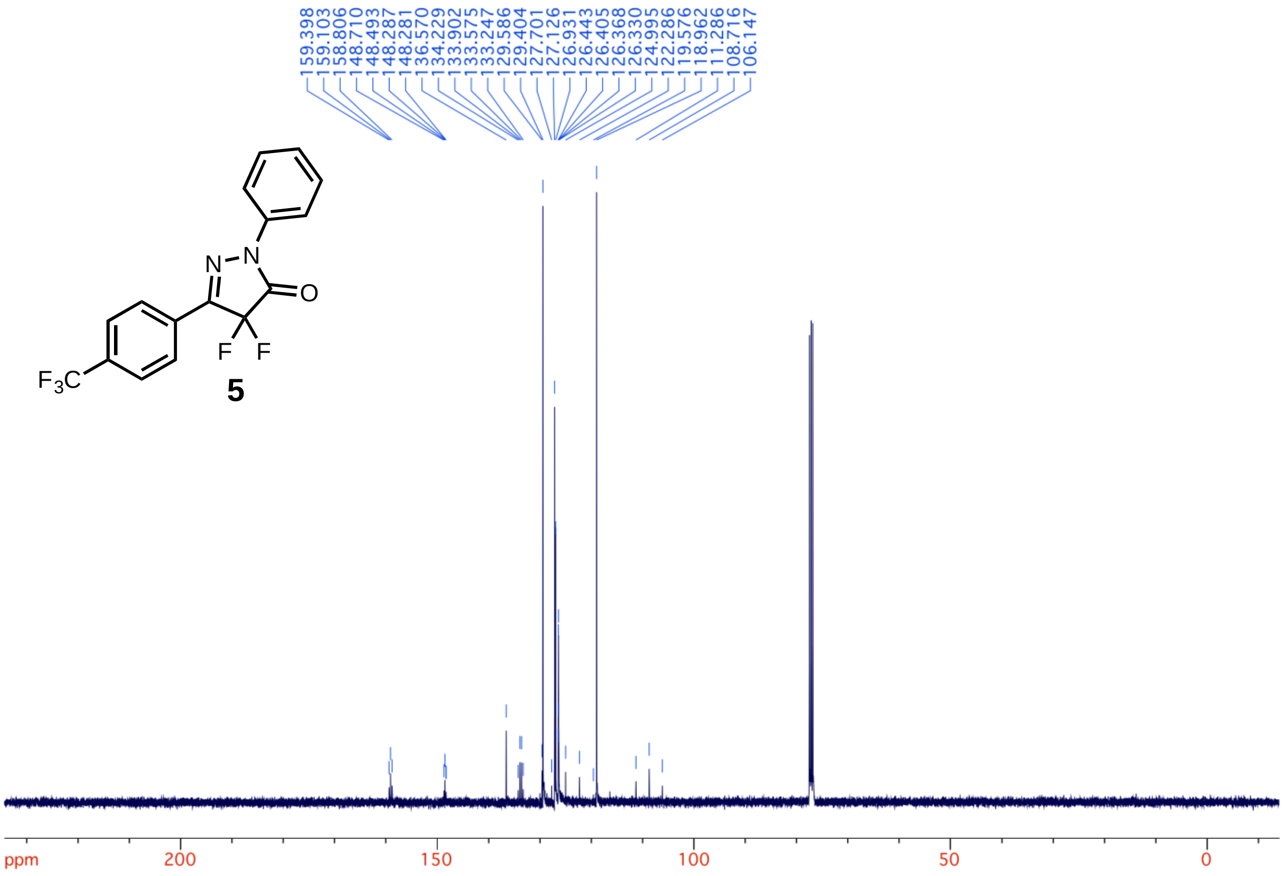
F3C
N
N
O
F F
5
159.398
159.103
158.806
148.710
148.493
148.287
148.281
136.570
134.229
133.902
133.575
133.247
129.586
129.404
127.701
127.126
126.931
126.443
126.405
126.368
126.330
124.995
122.286
119.576
118.962
111.286
108.716
106.147
ppm 200 150 100 50 0

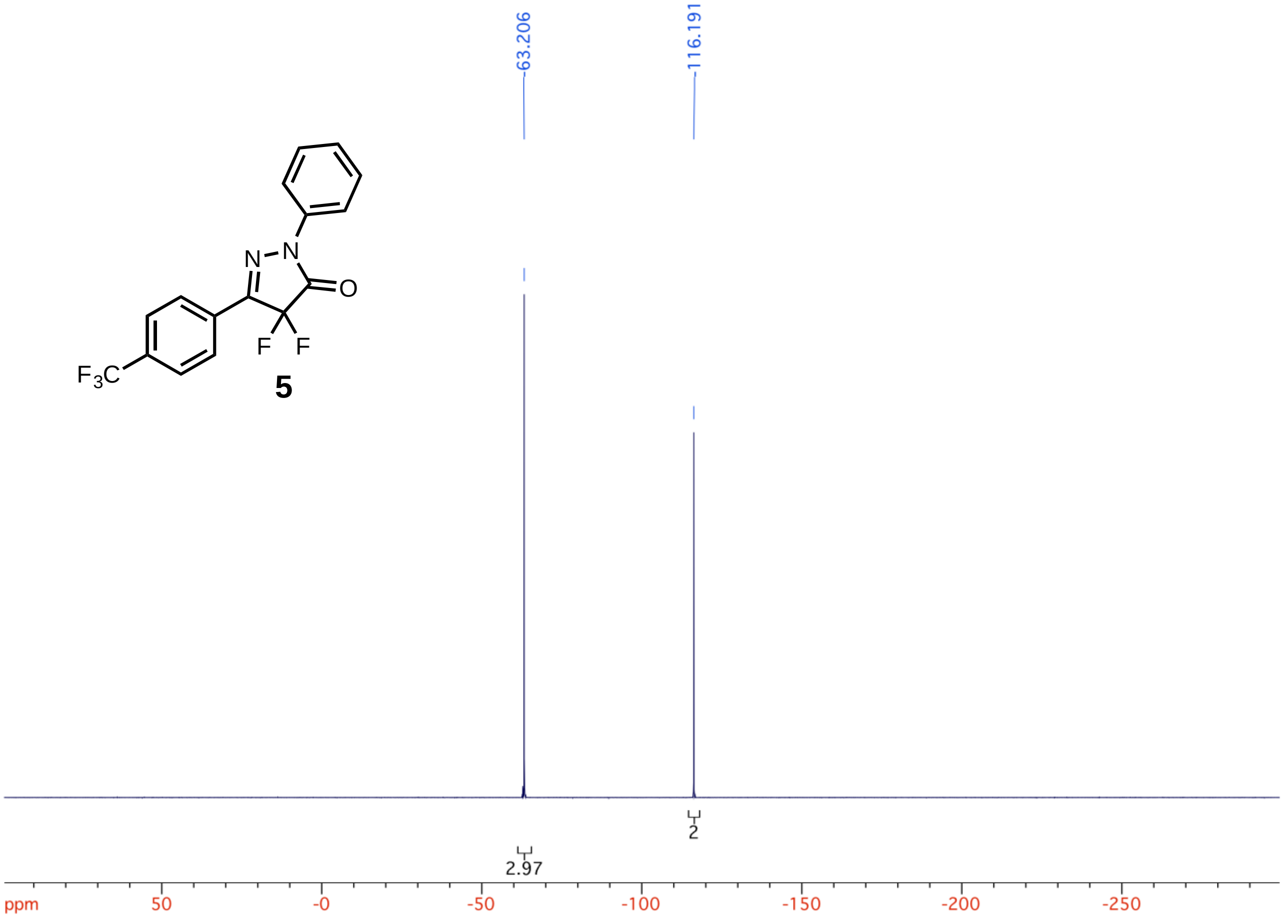

-63.206
-116.191
5
2.97
2
ppm
50
-0
-50
-100
-150
-200
-250

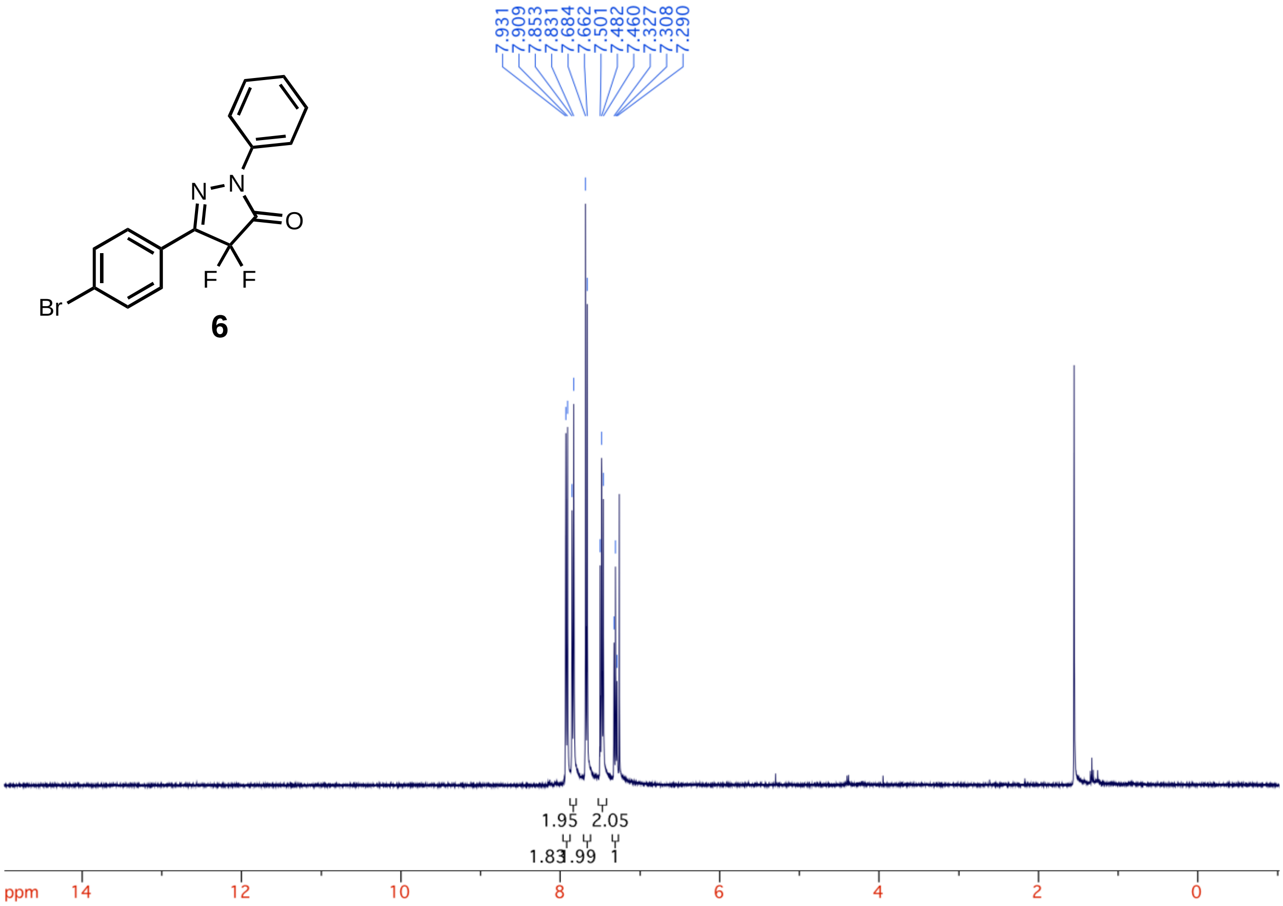
7.931
7.909
7.853
7.831
7.684
7.662
7.501
7.482
7.460
7.327
7.308
7.290
N
N
O
F
F
Br
6
1.95
2.05
1.83
1.99
1
ppm
14
12
10
8
6
4
2
0

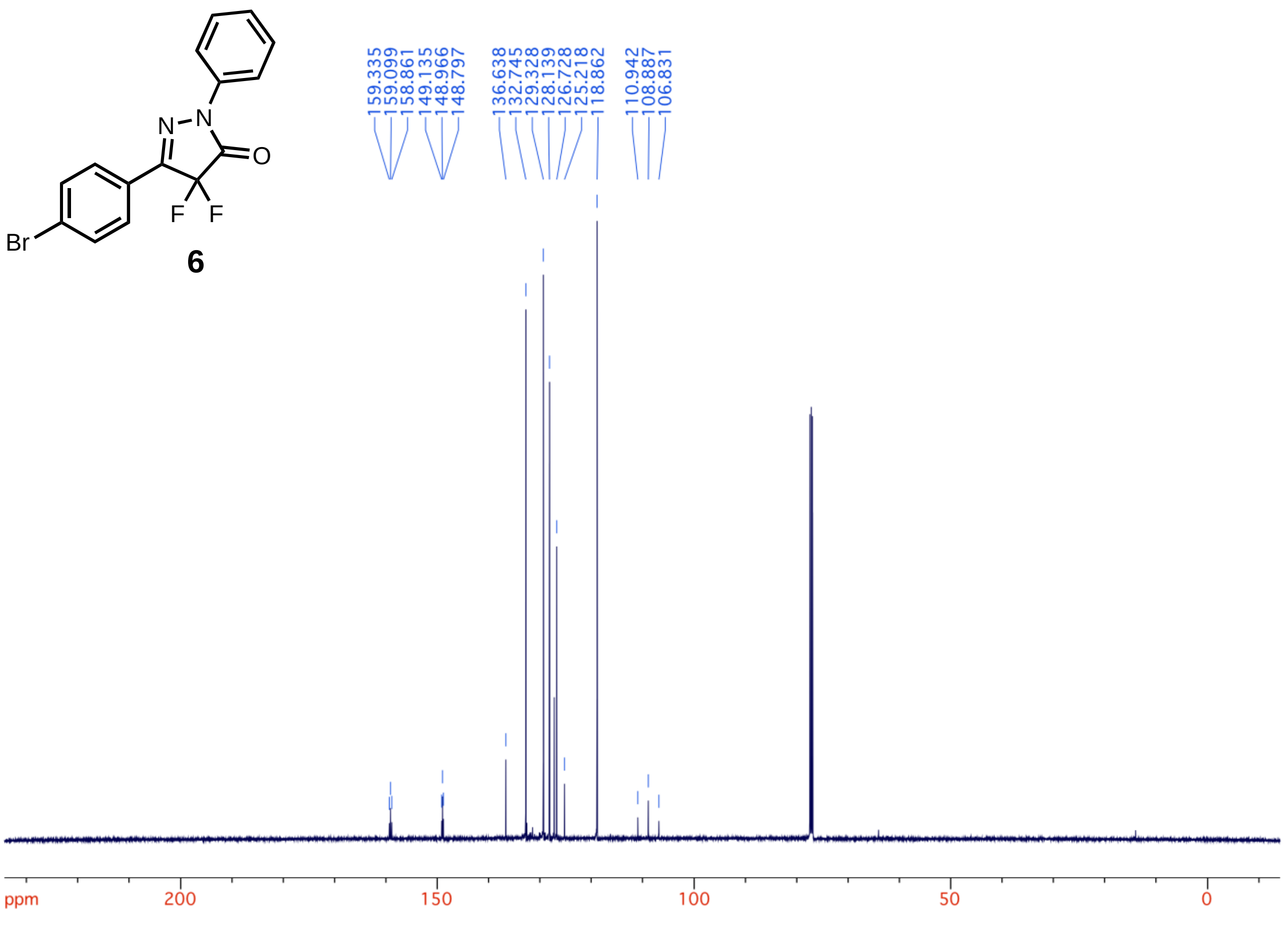
159.335
159.099
158.861
149.135
148.966
148.797
136.638
132.745
129.328
128.139
126.728
125.218
118.862
110.942
108.887
106.831
N
N
O
F
F
Br
6
ppm
200
150
100
50
0

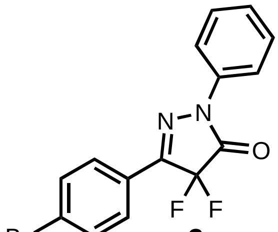

6

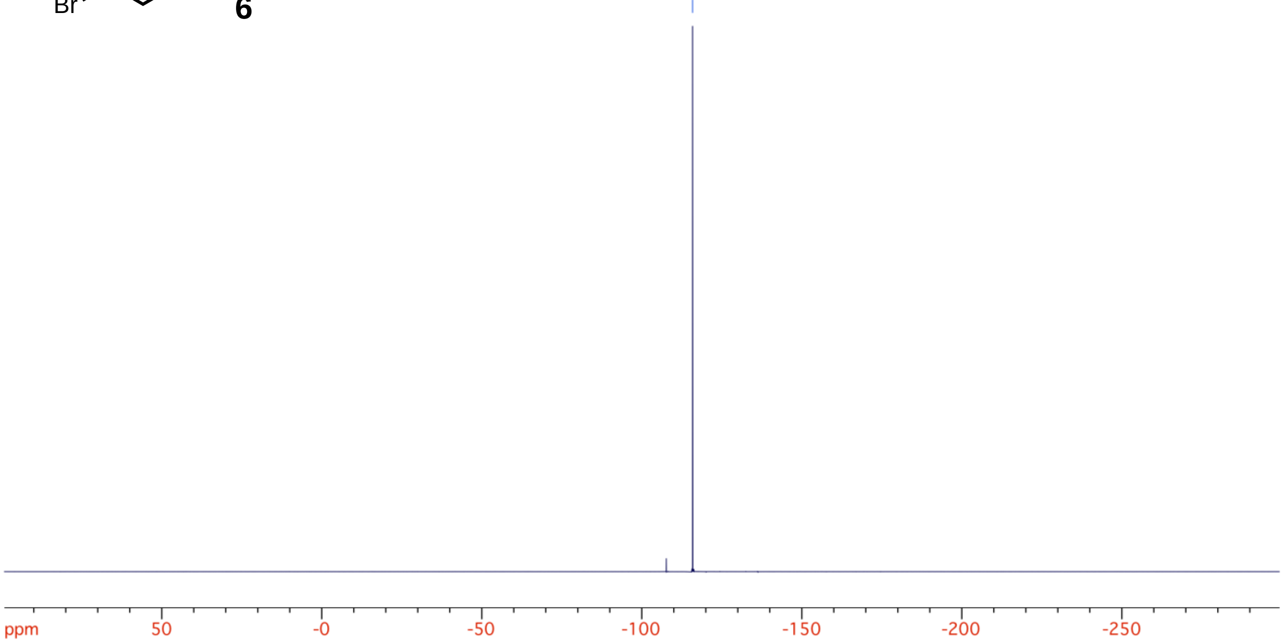

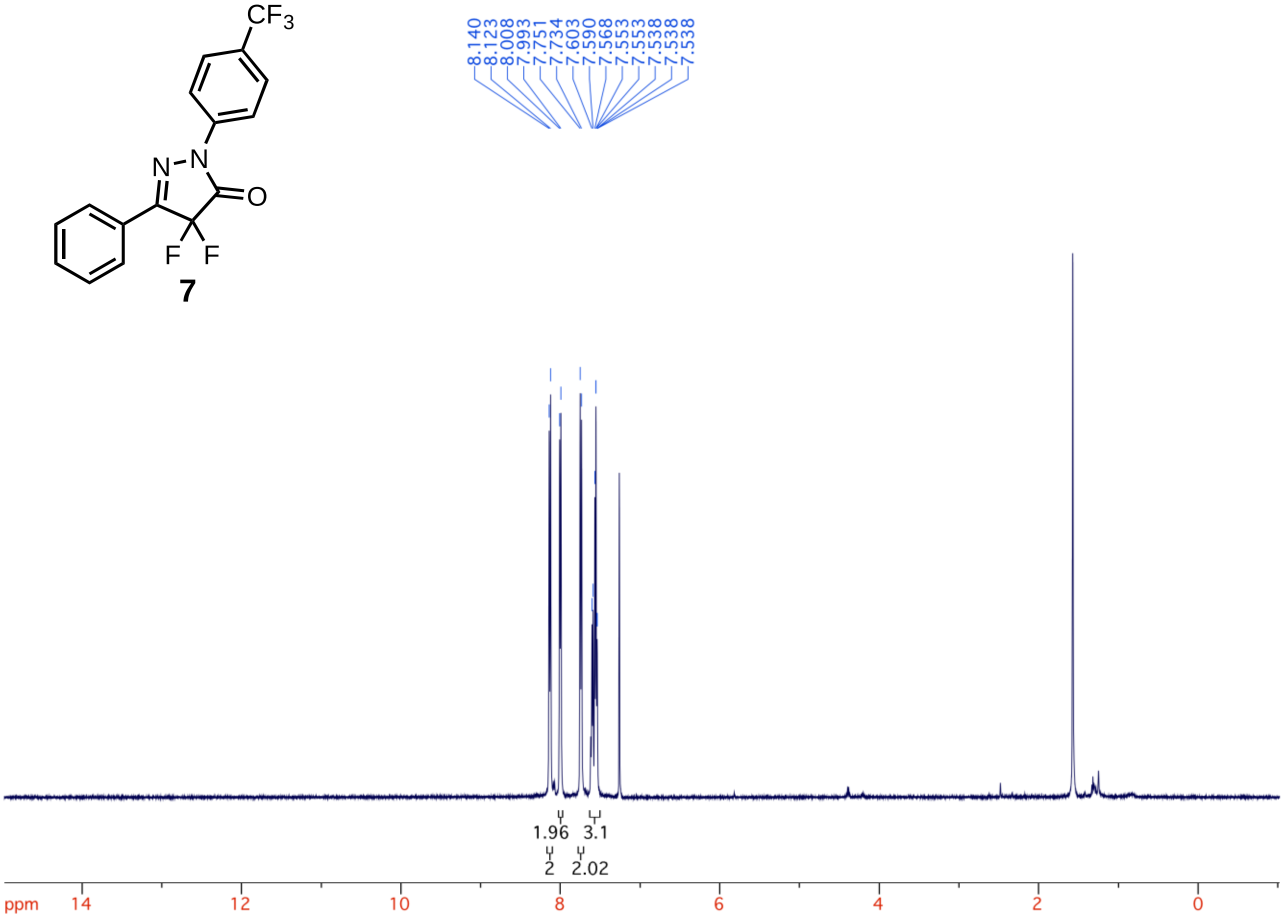
CF3
N
N
O
F F
7
8.140
8.123
8.008
7.993
7.751
7.734
7.603
7.590
7.568
7.553
7.553
7.538
7.538
7.538
1.96
3.1
2
2.02
ppm 14 12 10 8 6 4 2 0

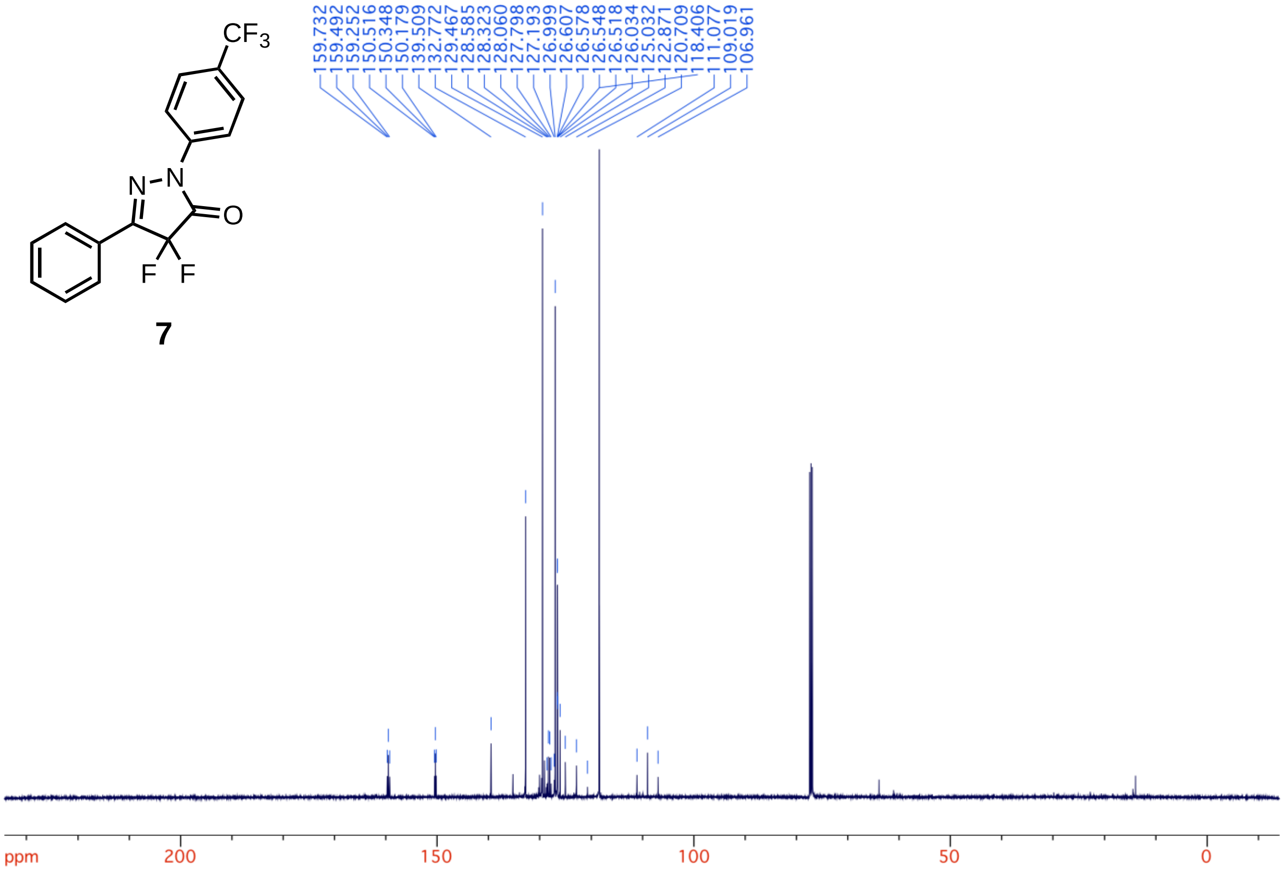
CF3
N
N
O
F F
7
159.732
159.492
159.252
150.516
150.348
150.179
139.509
132.772
129.467
128.585
128.323
128.060
127.798
127.193
126.999
126.607
126.578
126.548
126.518
126.034
125.032
122.871
120.709
118.406
111.077
109.019
106.961
ppm 200 150 100 50 0

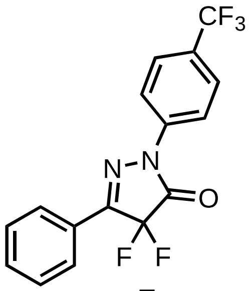

7

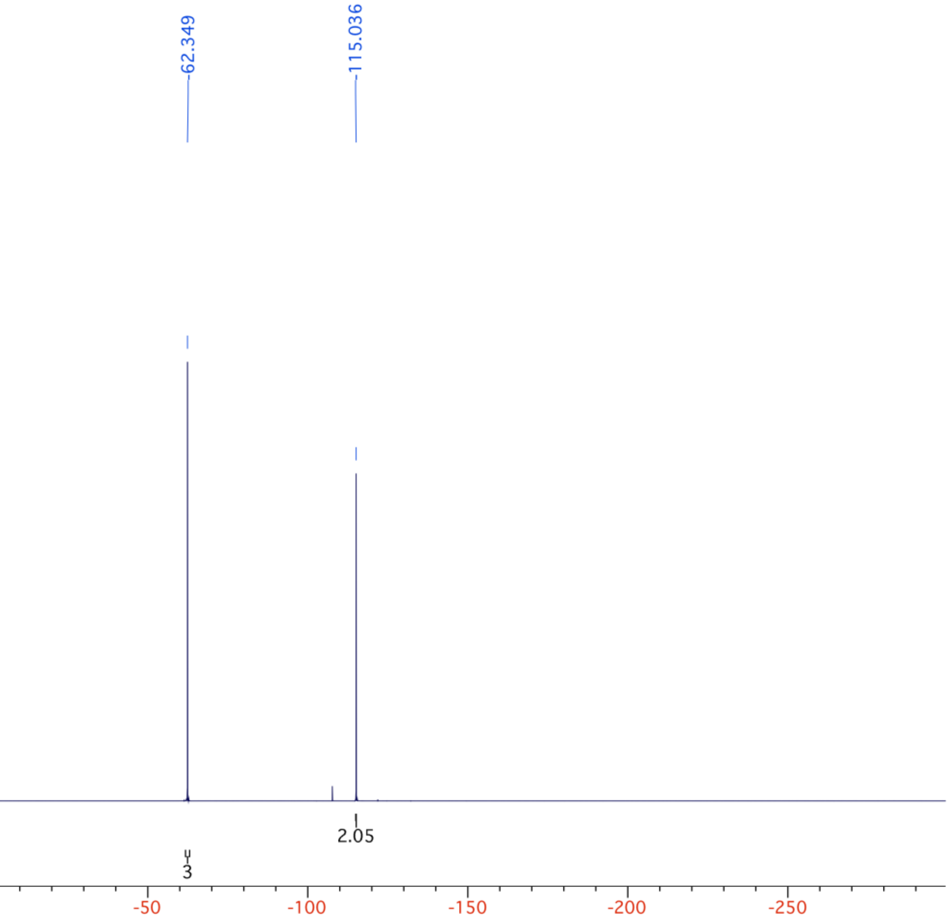

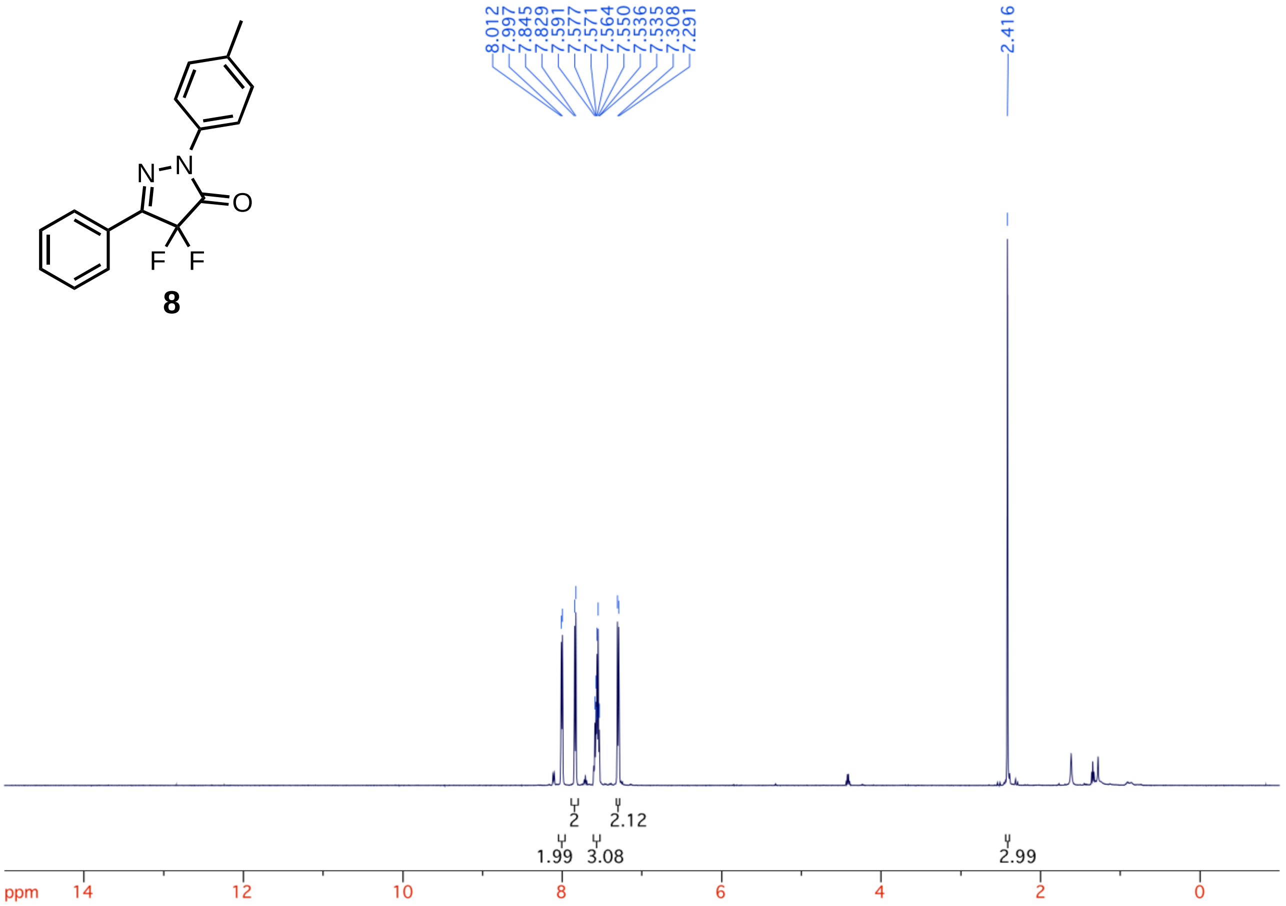
8
8.012
7.997
7.845
7.829
7.591
7.577
7.571
7.564
7.550
7.536
7.535
7.308
7.291
2.416
2
2.12
1.99
3.08
2.99
ppm 14 12 10 8 6 4 2 0

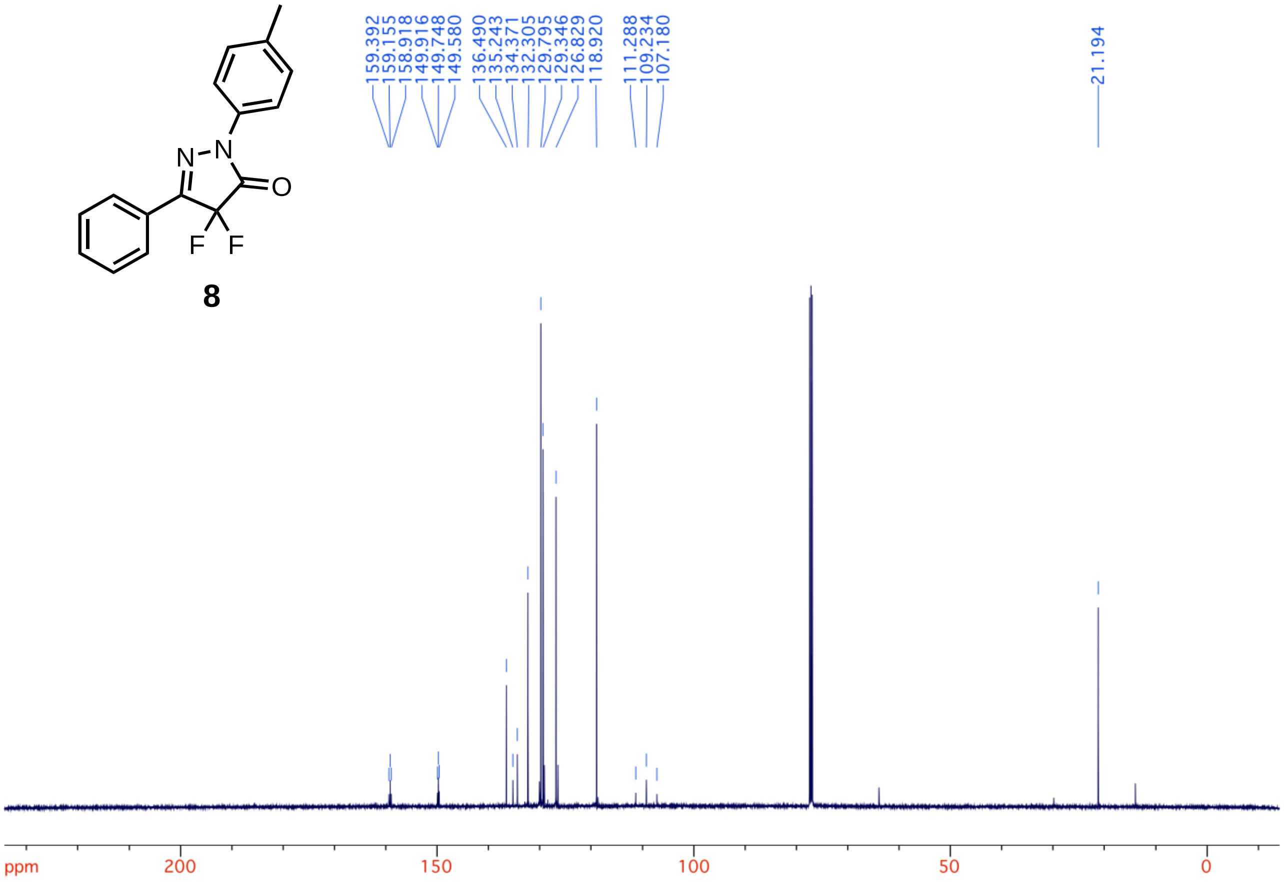
8
159.392
159.155
158.918
149.916
149.748
149.580
136.490
135.243
134.371
132.305
129.795
129.346
126.829
118.920
111.288
109.234
107.180
21.194
ppm 200 150 100 50 0

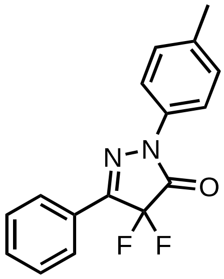

8

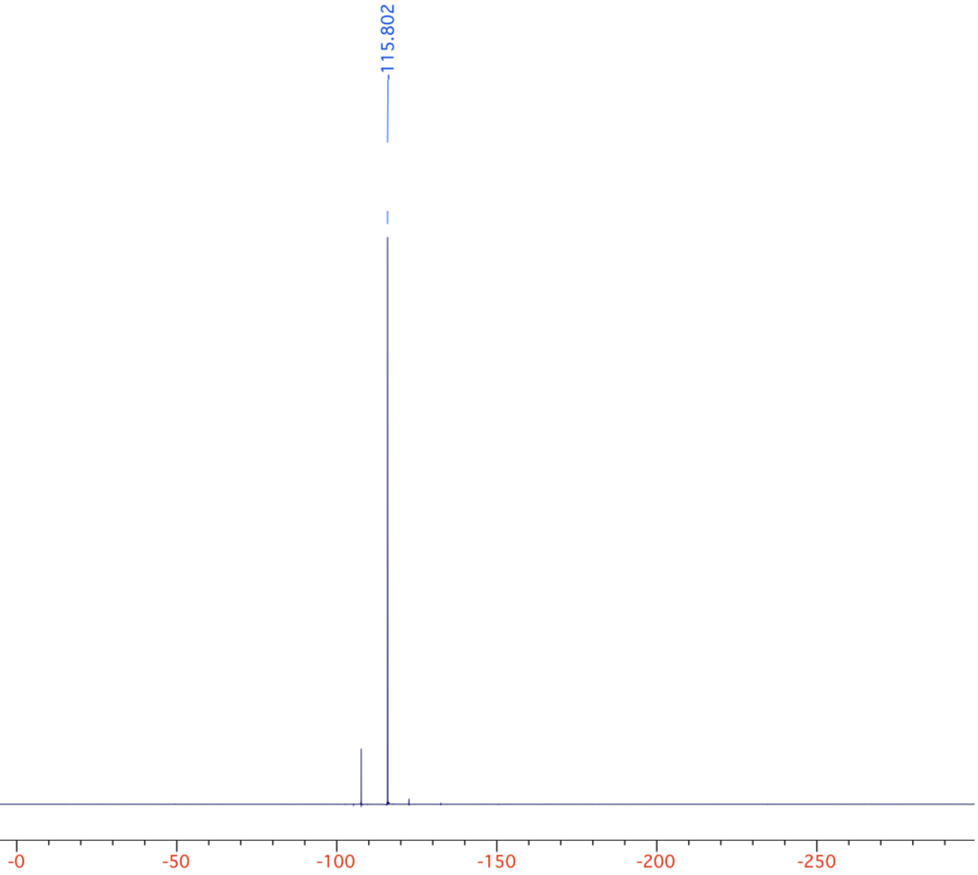

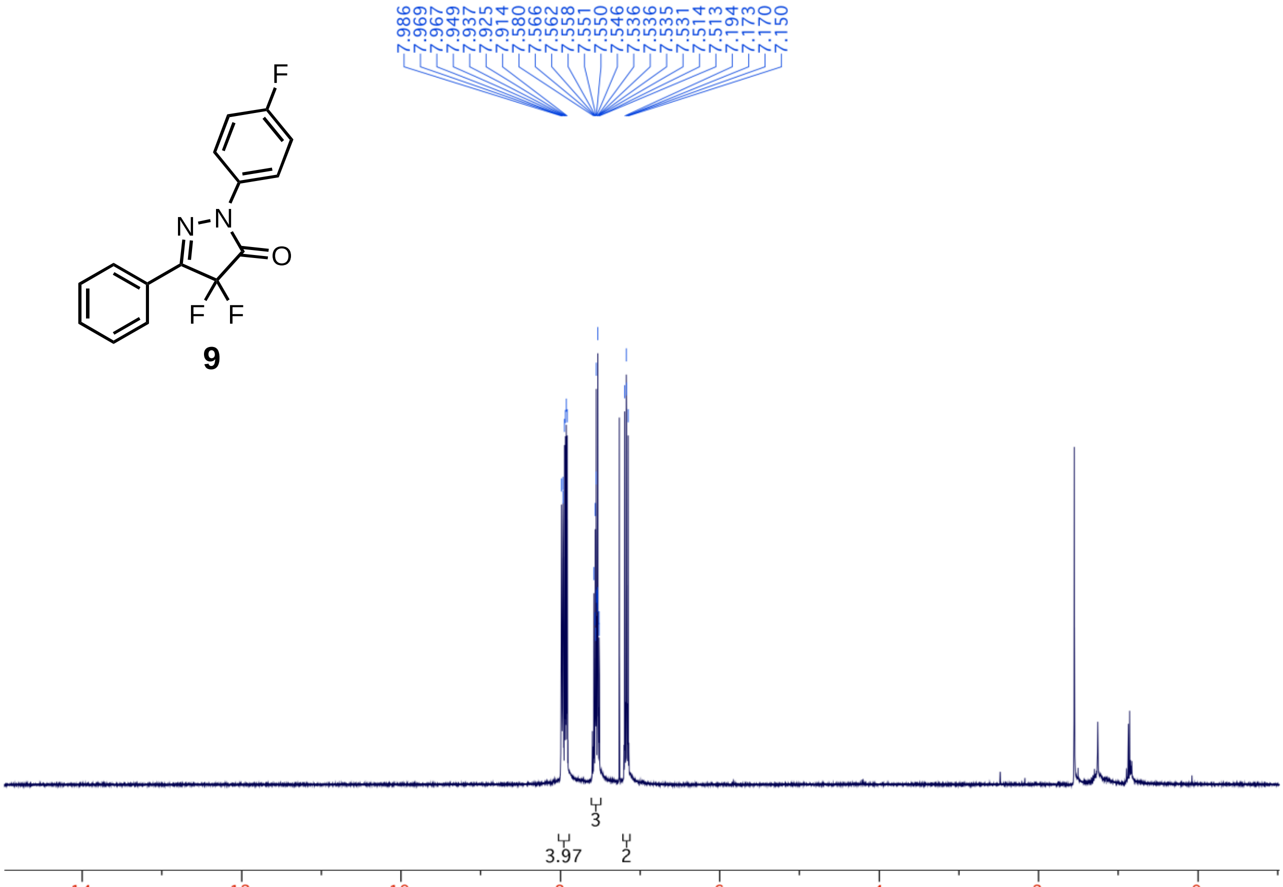
F
N
N
O
F
F
9
7.986
7.969
7.967
7.949
7.937
7.925
7.914
7.580
7.566
7.562
7.558
7.551
7.550
7.546
7.536
7.535
7.531
7.514
7.513
7.194
7.173
7.170
7.150
3
3.97
2
ppm
14
12
10
8
6
4
2
0

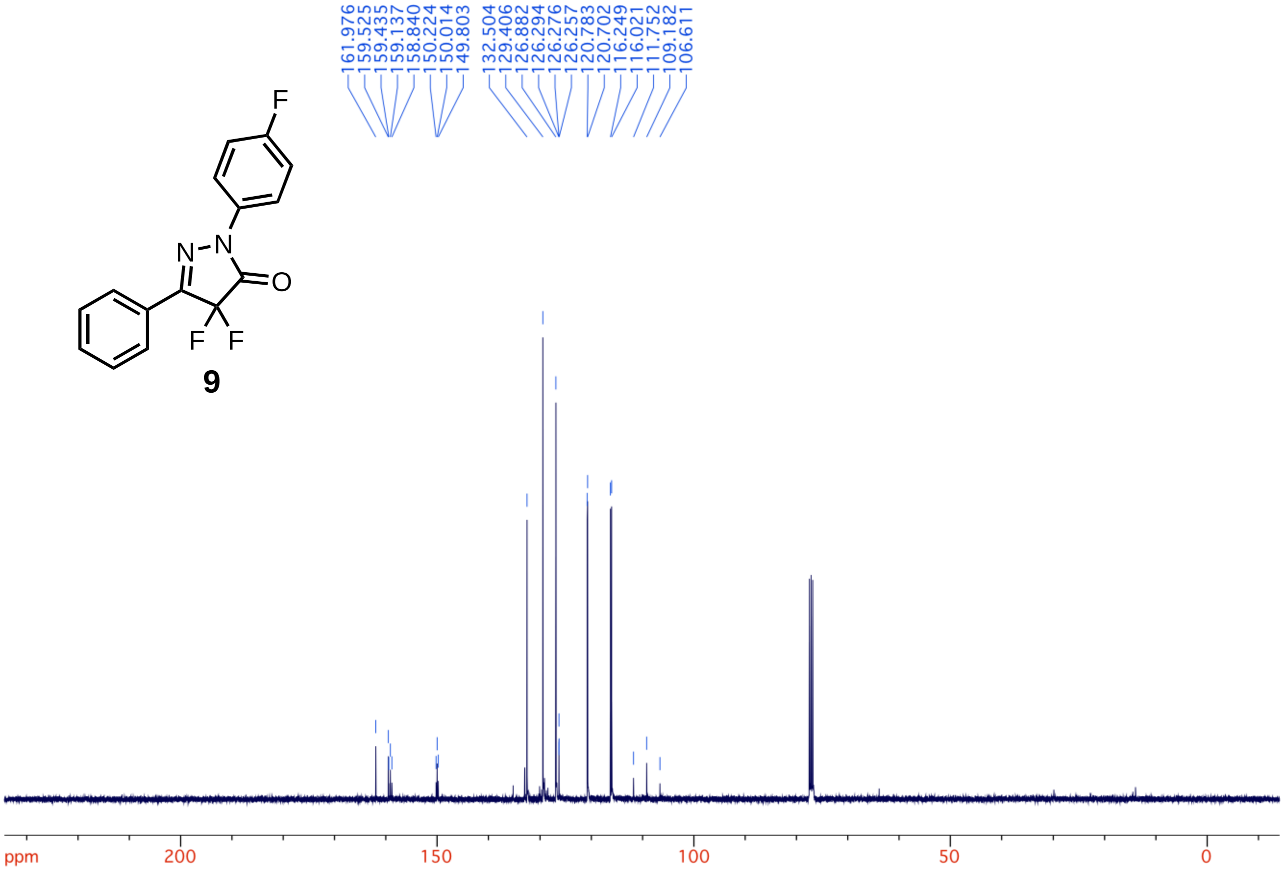
F
N
N
O
F
F
9
161.976
159.525
159.435
159.137
158.840
150.224
150.014
149.803
132.504
129.406
126.882
126.294
126.276
126.257
120.783
120.702
116.249
116.021
111.752
109.182
106.611
ppm
200
150
100
50
0

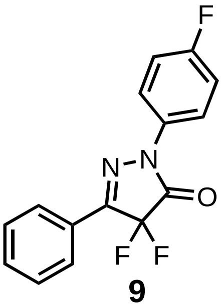

9

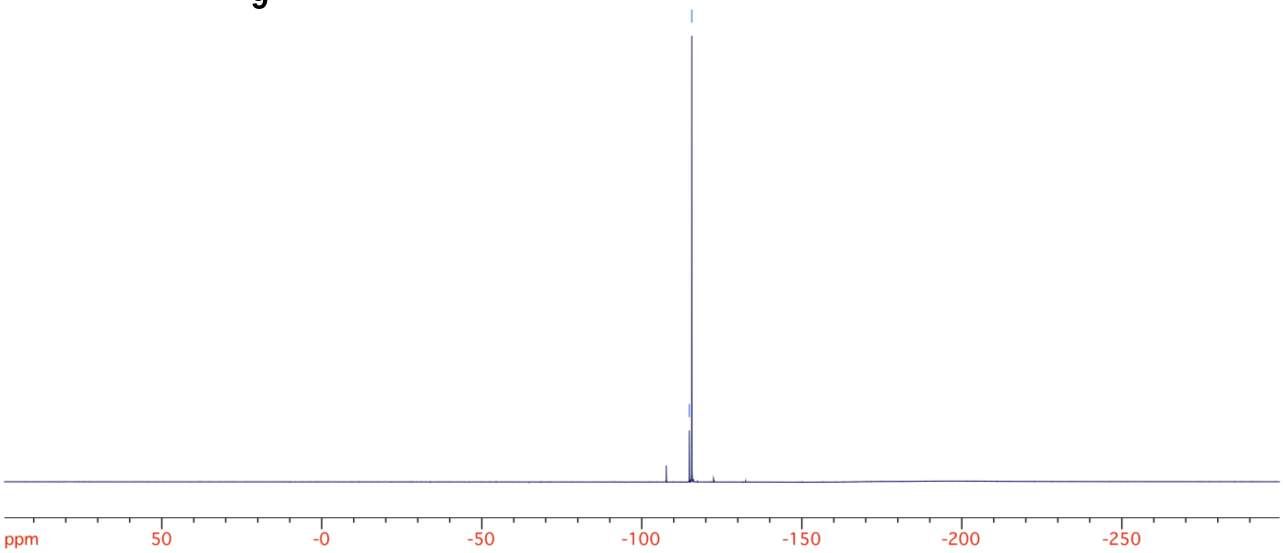

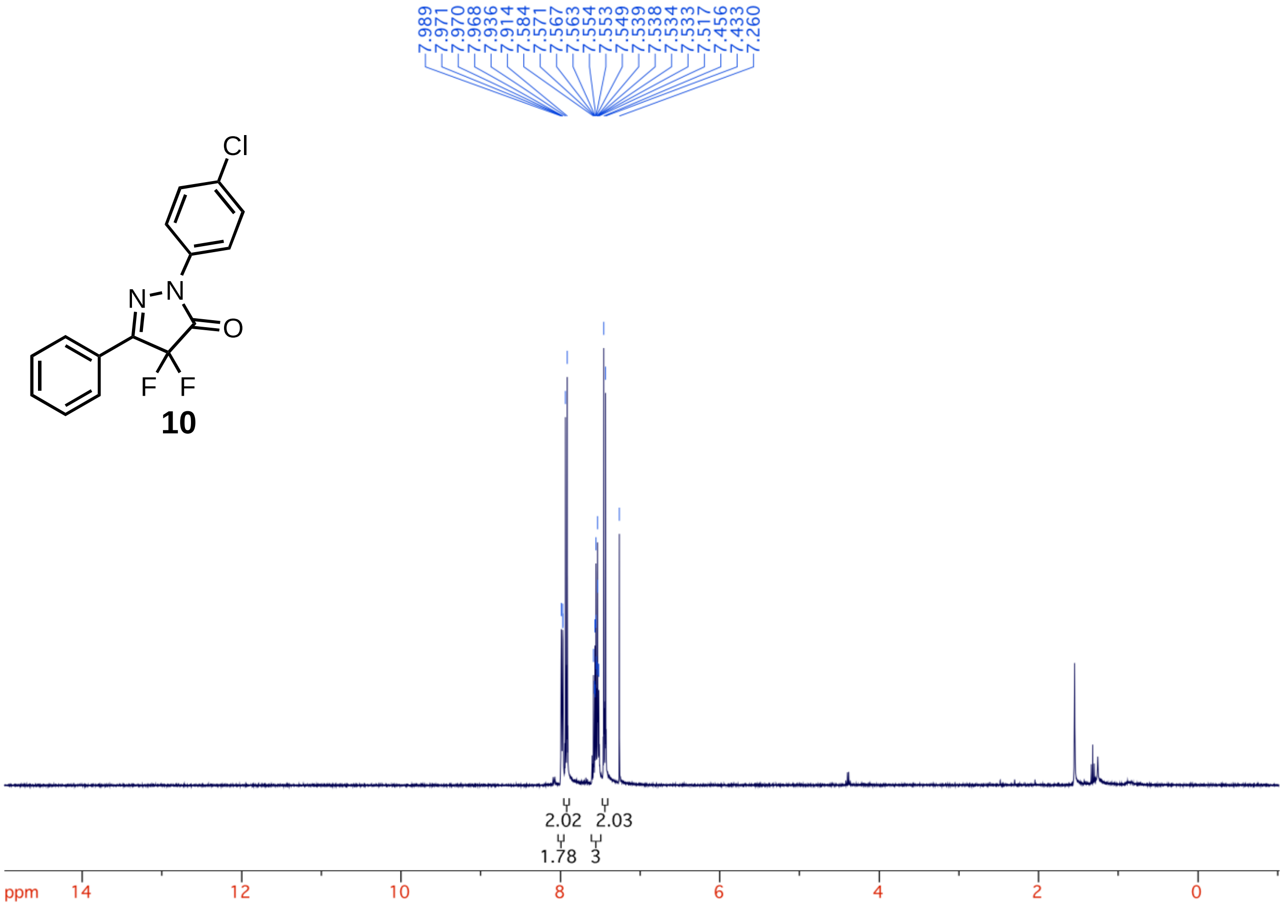
7.989
7.971
7.970
7.968
7.936
7.914
7.584
7.571
7.567
7.563
7.554
7.553
7.549
7.539
7.538
7.534
7.533
7.517
7.456
7.433
7.260
Cl
N
N
O
F
F
10
2.02
2.03
1.78
3
ppm
14
12
10
8
6
4
2
0

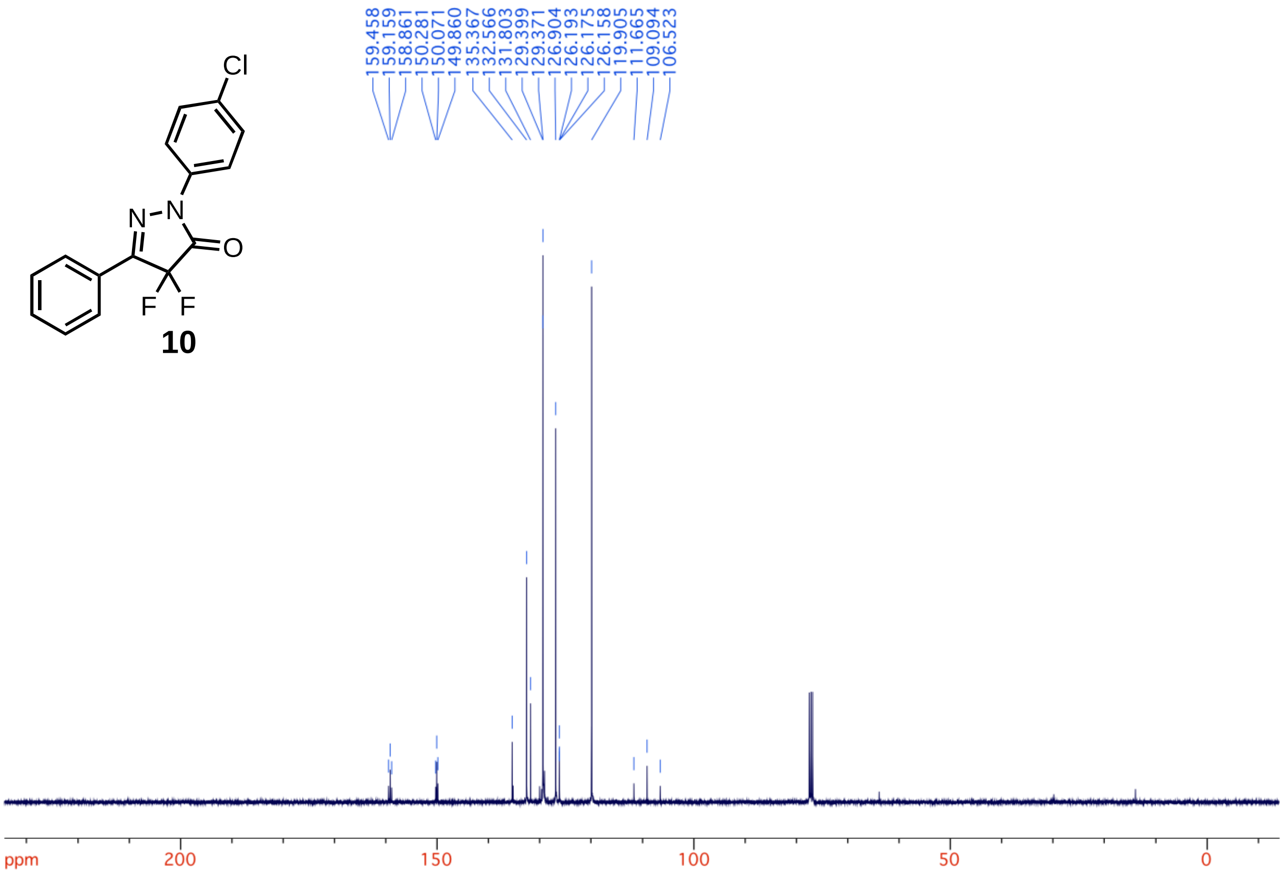
159.458
159.159
158.861
150.281
150.071
149.860
135.367
132.566
131.803
129.399
129.371
126.904
126.193
126.175
126.158
119.905
111.665
109.094
106.523
Cl
N
N
O
F
F
10
ppm
200
150
100
50
0

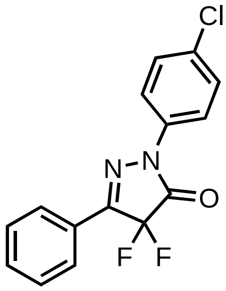

10

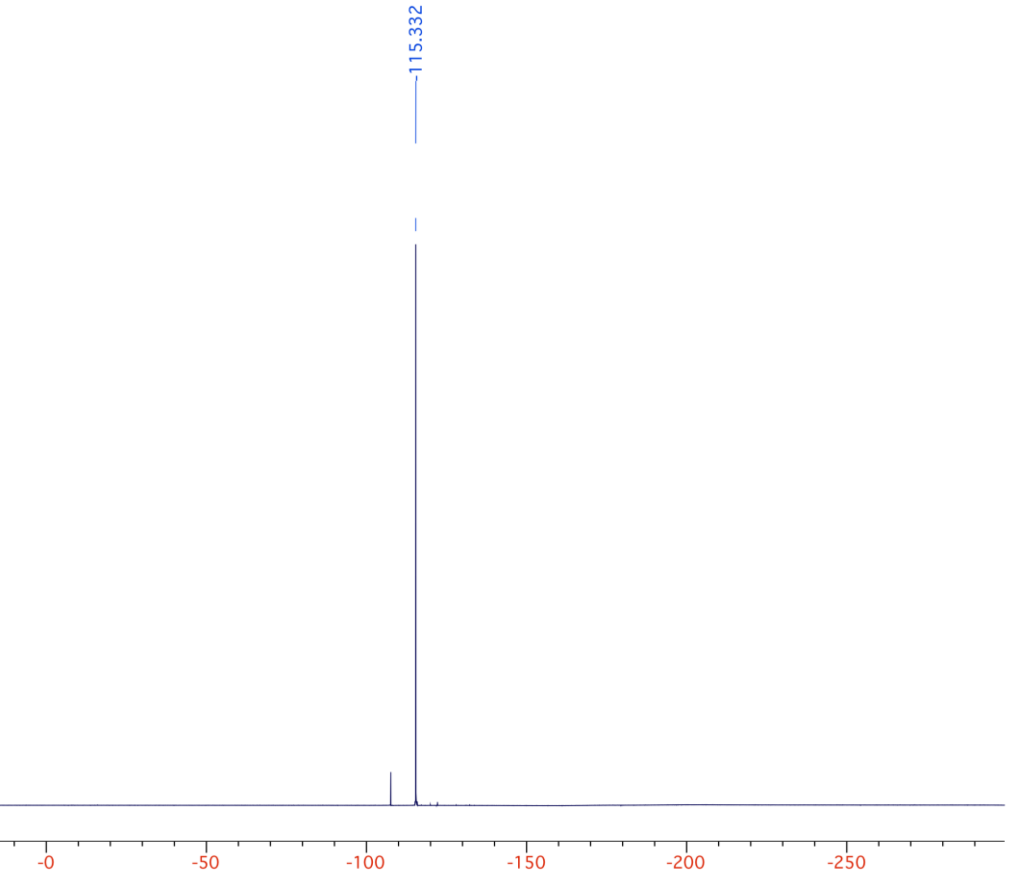

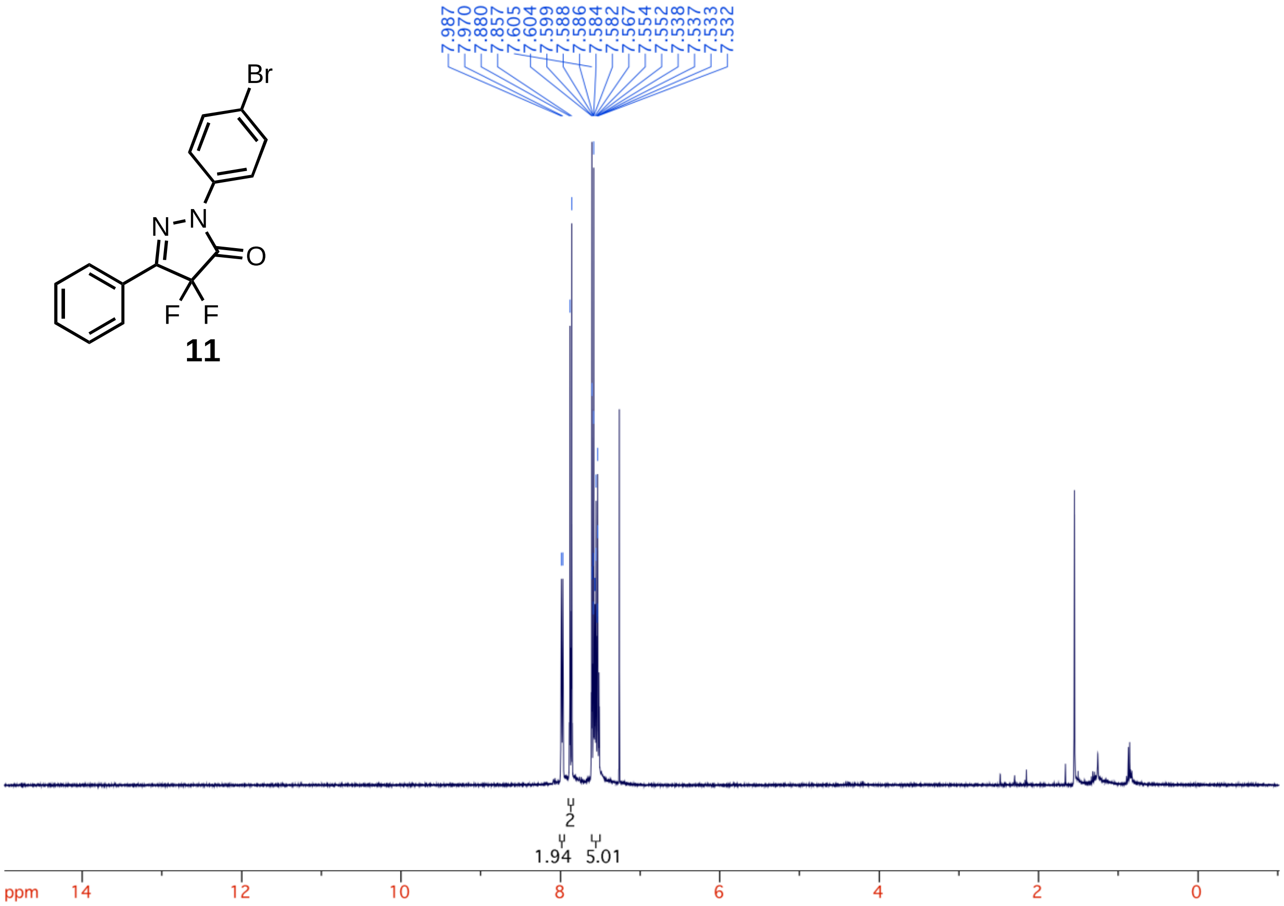
Br
N
N
O
F F
11
7.987
7.970
7.880
7.857
7.605
7.604
7.599
7.588
7.586
7.584
7.582
7.567
7.554
7.552
7.538
7.537
7.533
7.532
2
1.94
5.01
ppm 14 12 10 8 6 4 2 0

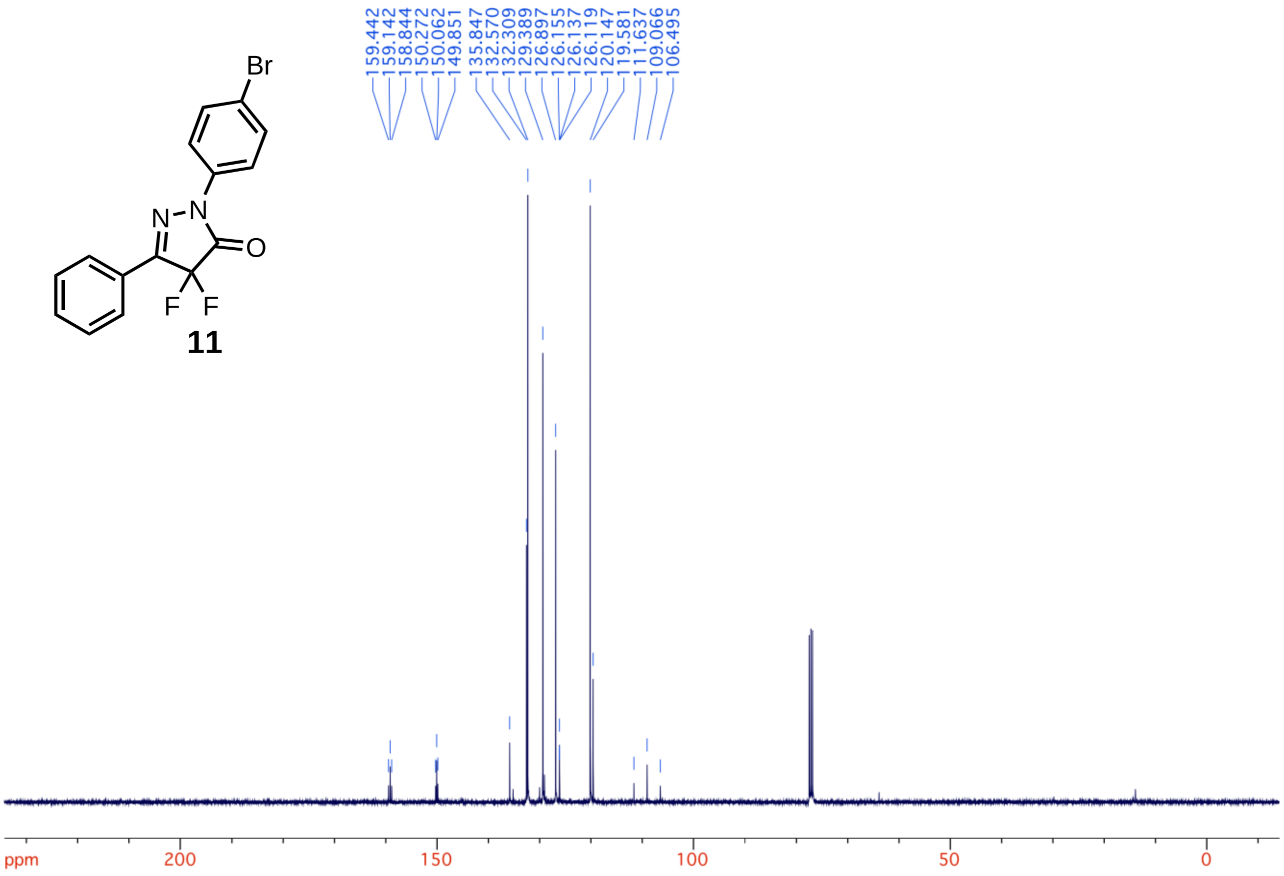
Br
N
N
O
F F
11
159.442
159.142
158.844
150.272
150.062
149.851
135.847
132.570
132.309
129.389
126.897
126.155
126.137
126.119
120.147
119.581
111.637
109.066
106.495
ppm 200 150 100 50 0

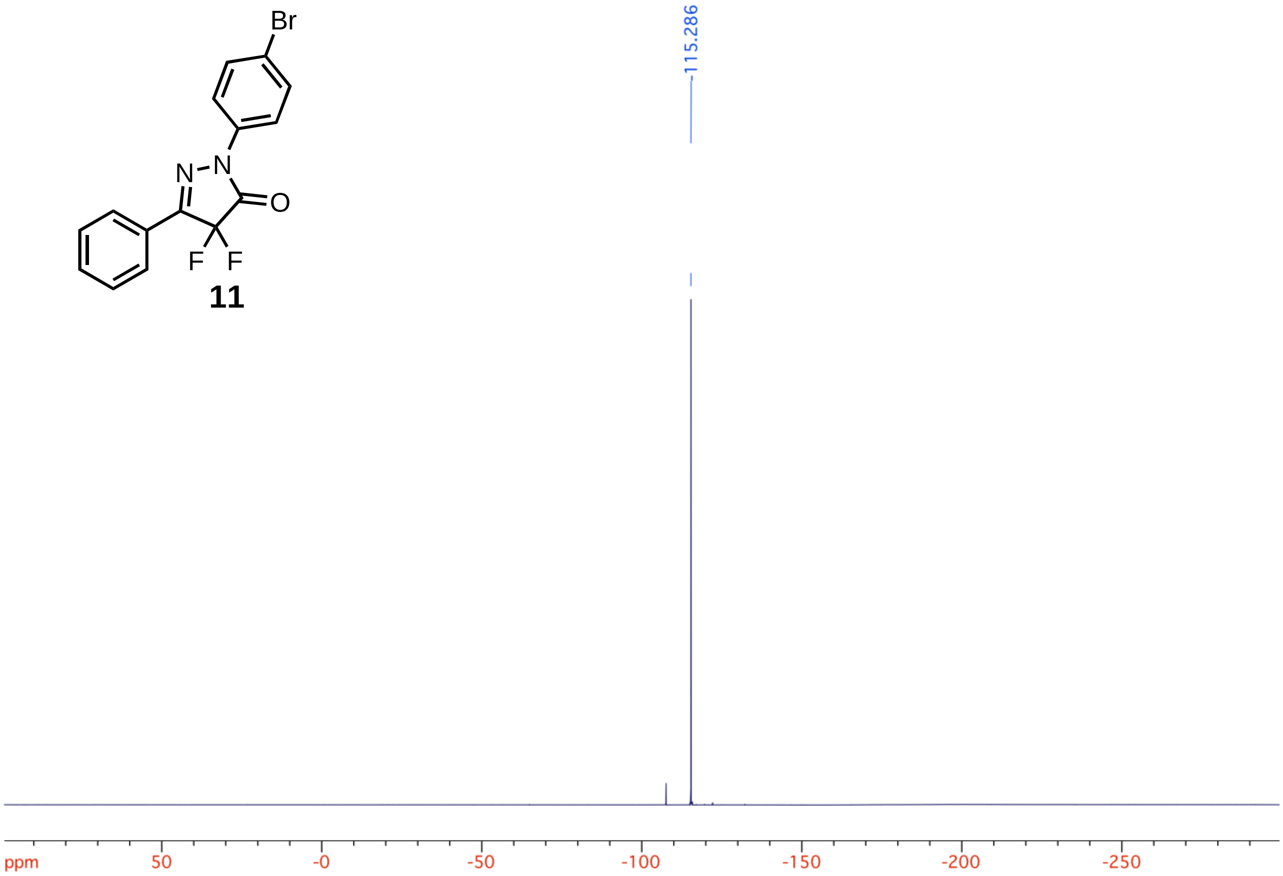

11

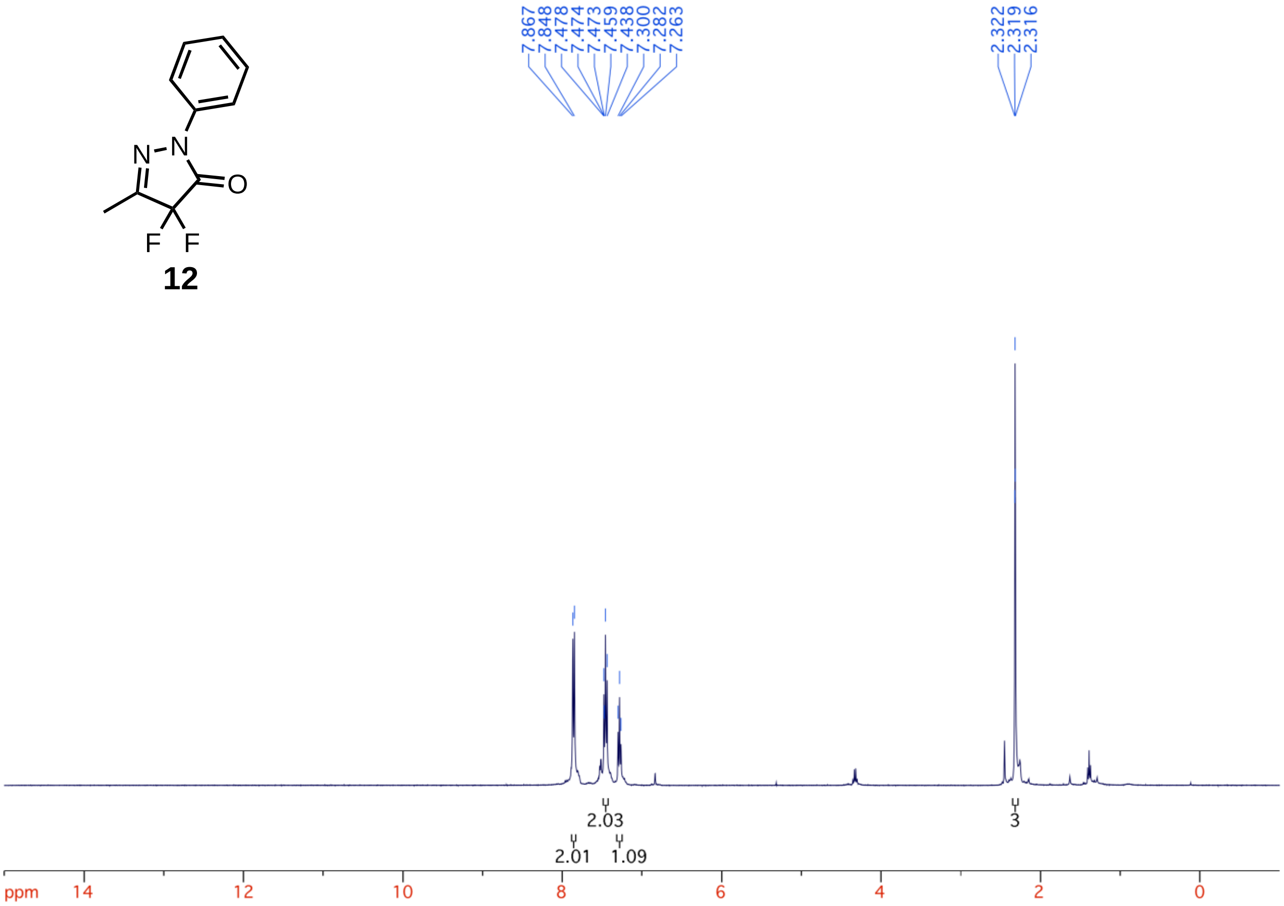
N
N
O
F
F
12
7.867
7.848
7.478
7.474
7.473
7.459
7.438
7.300
7.282
7.263
2.322
2.319
2.316
2.03
3
2.01
1.09
ppm
14
12
10
8
6
4
2
0

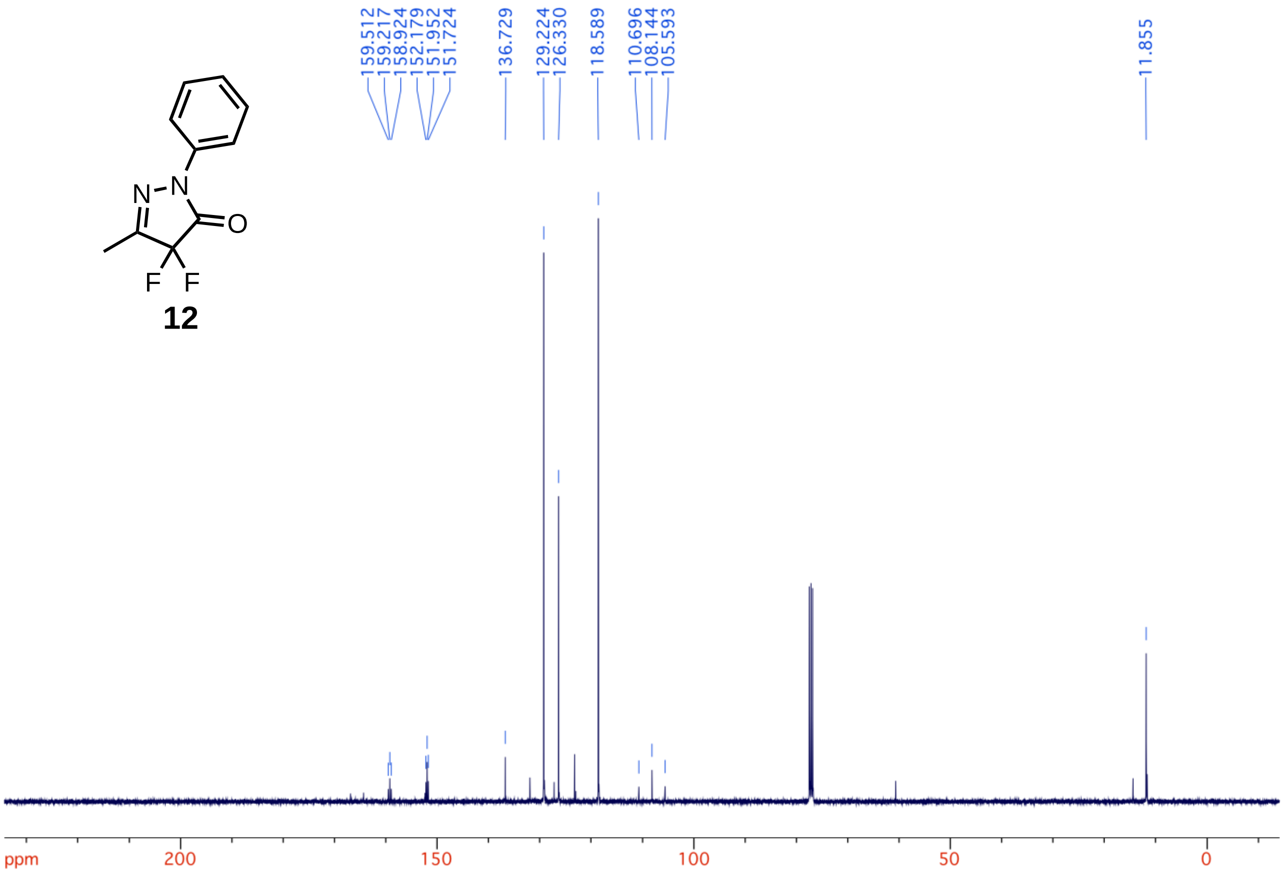
N
N
O
F
F
12
159.512
159.217
158.924
152.179
151.952
151.724
136.729
129.224
126.330
118.589
110.696
108.144
105.593
11.855
ppm
200
150
100
50
0

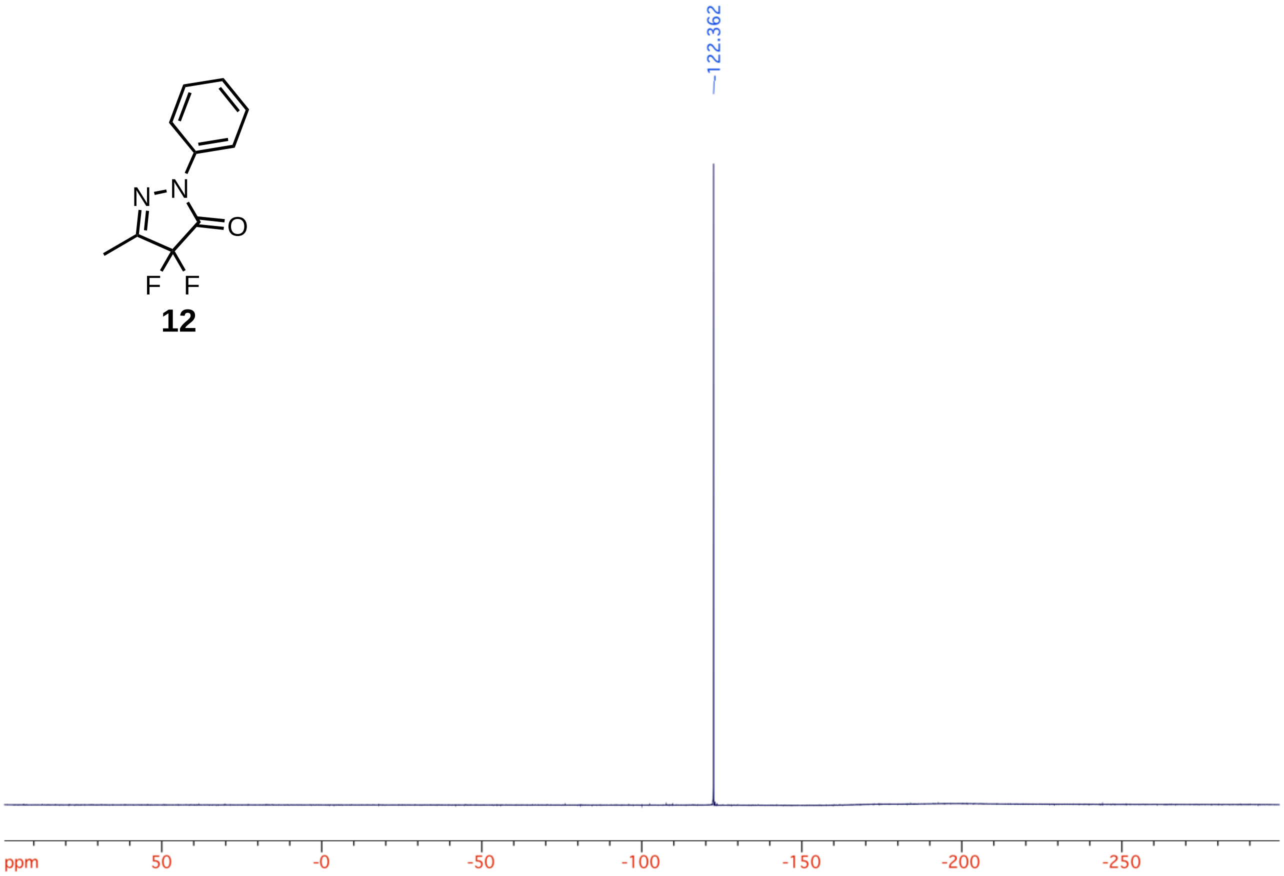

12

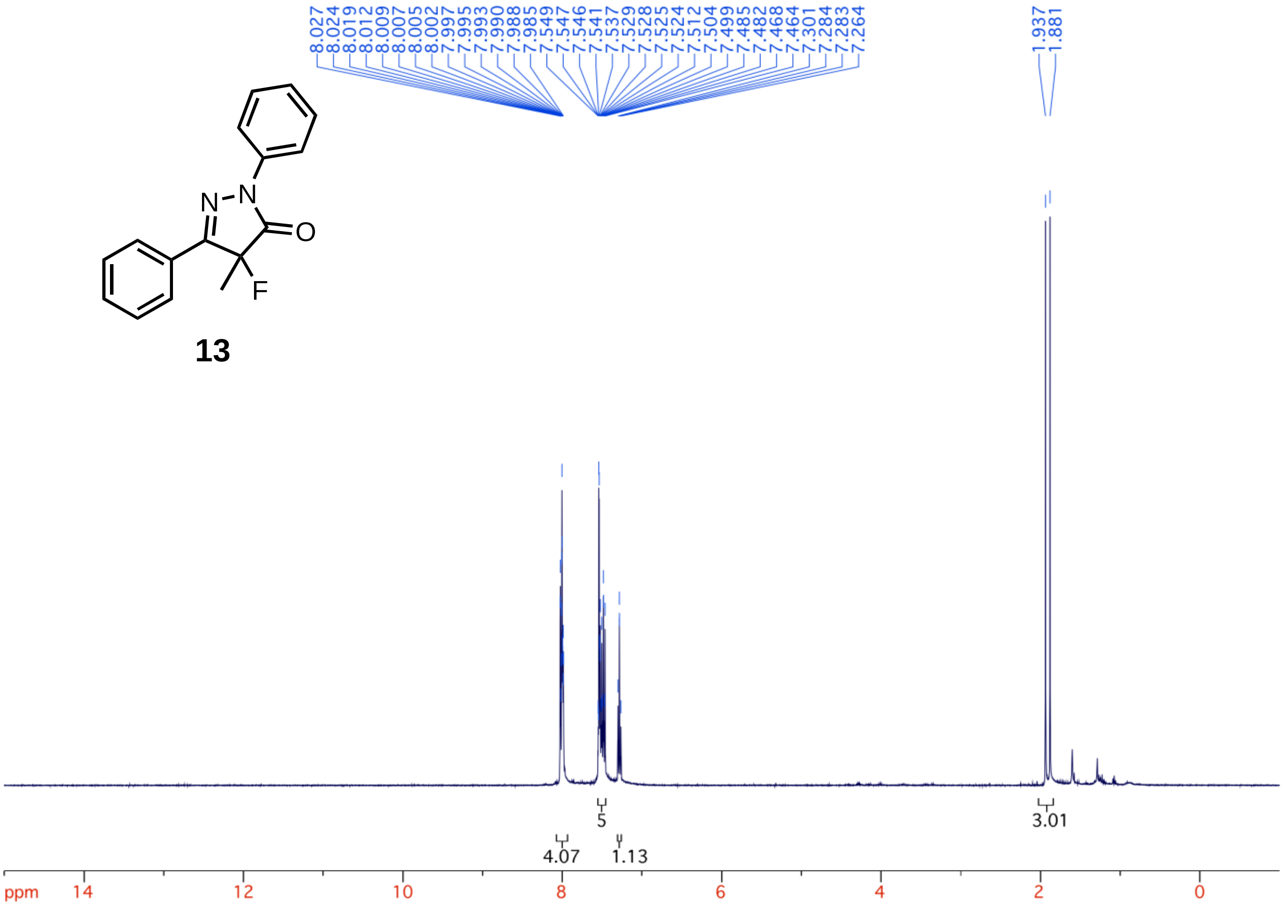
8.027
8.024
8.019
8.012
8.009
8.007
8.005
8.002
7.997
7.995
7.993
7.990
7.988
7.985
7.549
7.546
7.541
7.537
7.529
7.528
7.525
7.524
7.512
7.504
7.499
7.485
7.482
7.468
7.464
7.301
7.284
7.283
7.264
1.937
1.881
N
N
O
F
13
5
3.01
4.07
1.13
ppm
14
12
10
8
6
4
2
0

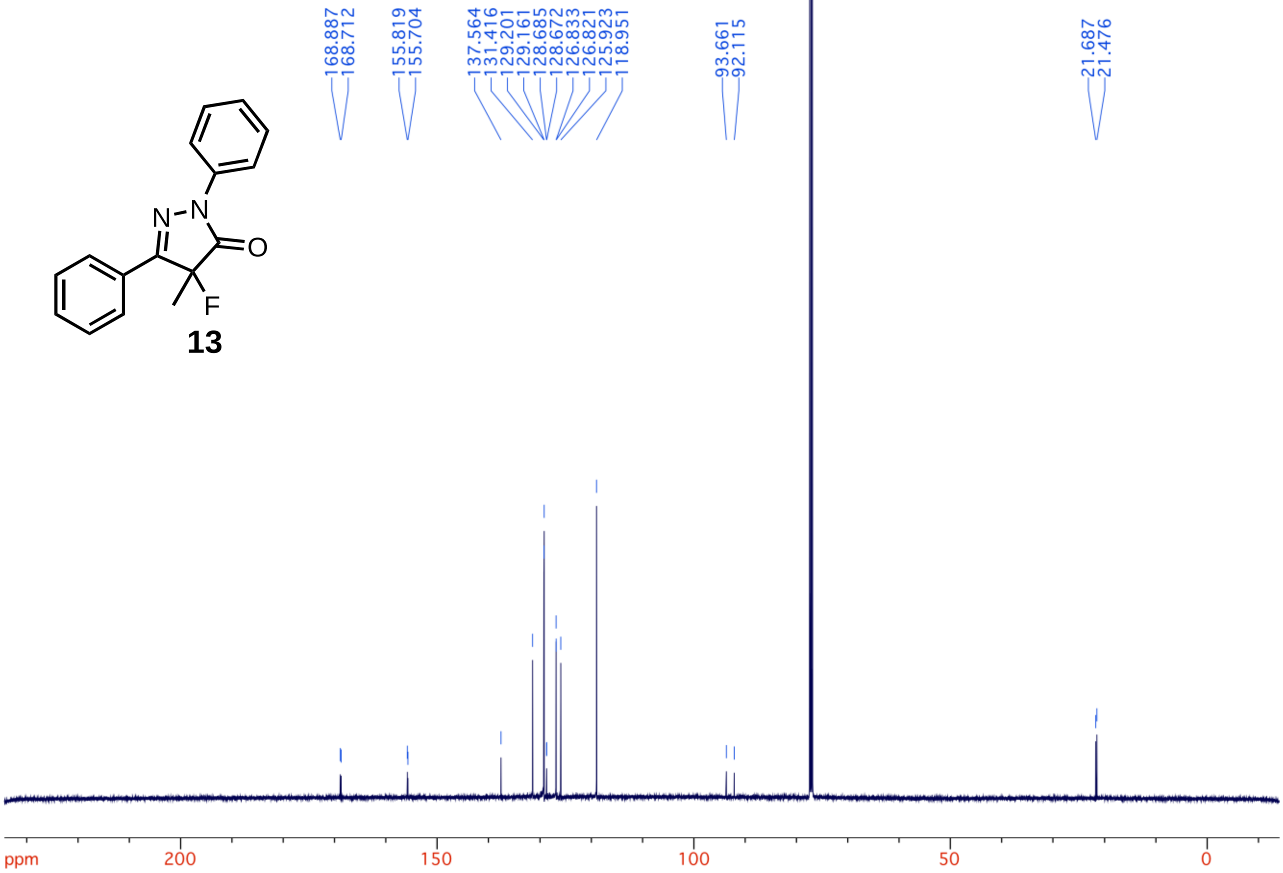
168.887
168.712
155.819
155.704
137.564
131.416
129.201
129.161
128.685
128.672
126.833
126.821
125.923
118.951
93.661
92.115
21.687
21.476
N
N
O
F
13
ppm
200
150
100
50
0

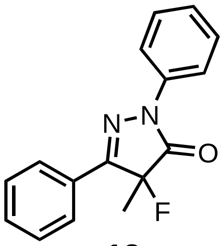

13

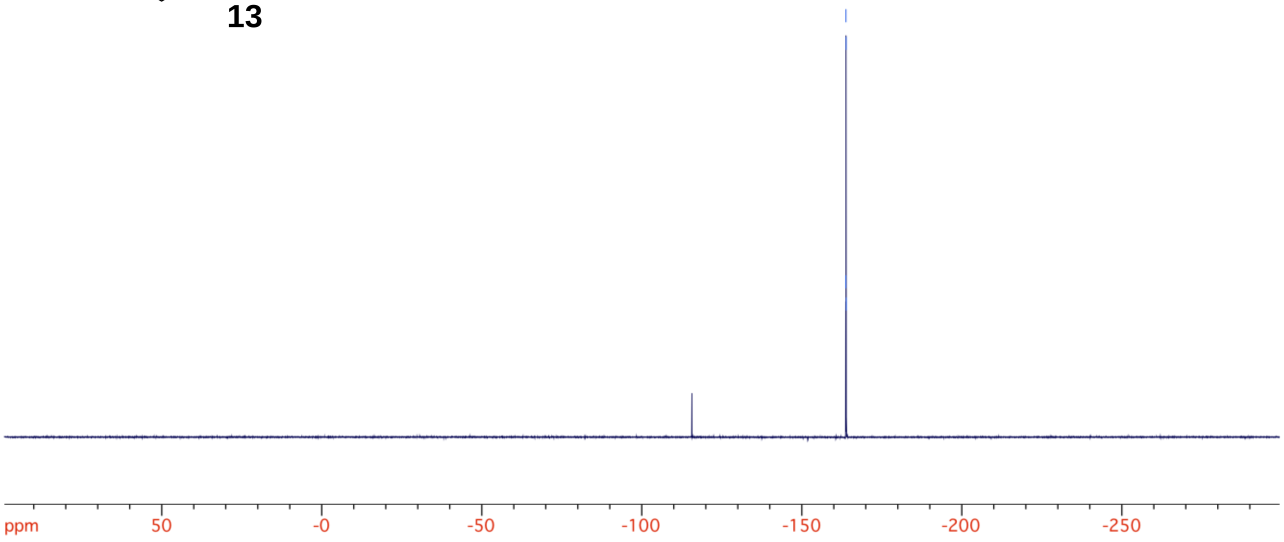

References

1 P. Drouhin and R. J. K. Taylor, *European J. Org. Chem.*, 2015, **2015**, 2333–2336.

2 C. V Galliford and K. a Scheidt, *Chem. Commun. (Camb).*, 2008, 1926–8.

3 S. Hok and N. E. Schore, *J. Org. Chem.*, 2006, **71**, 1736–1738.

4 L. Huck, M. Berton, A. de la Hoz, A. Díaz-Ortiz and J. Alcázar, *Green Chem.*, 2017, **19**, 1420–1424.

5 T. Hellmuth, W. Frey and R. Peters, *Angew. Chem. Int. Ed.*, 2015, **54**, 2788–2791.

6 R. Surmont, G. Verniest and N. De Kimpe, *Org. Lett.*, 2010, **12**, 4648–4651.

7 W. Ying, D. D. DesMarteau, Z.-Q. Xu and M. Witz, *J. Fluor. Chem.*, 2000, **102**, 135–139.
